# Impact of Near‐Positivity Violations on IPTW‐Estimated Marginal Structural Survival Models With Time‐Dependent Confounding

**DOI:** 10.1002/bimj.70093

**Published:** 2025-11-03

**Authors:** Marta Spreafico

**Affiliations:** ^1^ Mathematical Institute Leiden University Leiden The Netherlands; ^2^ Department of Biomedical Data Sciences Leiden University Medical Center Leiden The Netherlands

**Keywords:** inverse probability of treatment weighting, marginal structural models, positivity assumption, simulation studies, survival outcomes

## Abstract

In longitudinal observational studies, marginal structural models (MSMs) are used to analyze the causal effect of an exposure on the (time‐to‐event) outcome of interest, while accounting for exposure‐affected time‐dependent confounding. In the applied literature, inverse probability of treatment weighting (IPTW) has been widely adopted to estimate MSMs. An essential assumption for IPTW‐based MSMs is *positivity*, which requires that, for any combination of measured confounders among individuals, there is a nonzero probability of receiving each treatment strategy. Positivity is crucial for valid causal inference through IPTW‐based MSMs, but is often overlooked compared to confounding bias. Near‐positivity violations, where certain treatments are theoretically possible but rarely observed due to randomness, are common in practical applications, particularly when the sample size is small, and they pose significant challenges for causal inference. This study investigates the impact of near‐positivity violations on estimates from IPTW‐based MSMs in survival analysis. Two algorithms are proposed for simulating longitudinal data from hazard‐MSMs, accommodating near‐positivity violations, a time‐varying binary exposure, and a time‐to‐event outcome. Cases of near‐positivity violations, where remaining unexposed is rare within certain confounder levels, are analyzed across various scenarios and weight truncation (WT) strategies. Through comprehensive simulations, this study shows that even minor near‐positivity violations in longitudinal survival analyses can substantially destabilize IPTW‐based estimators, inflating variance and bias, especially under aggressive WT. This work aims to serve as a critical warning against overlooking the positivity assumption or naively applying WT in causal studies using longitudinal observational data and IPTW.

## 1 Introduction

In longitudinal observational studies, exposure‐affected time‐varying confounding represents a major challenge for estimating the effect of a treatment on the (time‐to‐event) outcome of interest, as standard analyses fail to give consistent estimators (Clare et al. 2019; Daniel et al. 2013). Over the past decades, considerable progress has been made in developing causal inference methods tailored to such complex data. Among these, marginal structural models (MSMs) offer a rigorous modeling approach for estimating and summarizing longitudinal causal effects in the presence of time‐dependent confounding (Daniel et al. 2013; Hernán et al. 2000; Hernán and Robins 2020; Robins et al. 2000; Williamson and Ravani 2017). MSMs are particularly useful when multiple time‐varying treatment regimens are possible, as they help synthesize complex treatment histories into interpretable summaries, making them highly valuable for applied work. MSMs are models for the *potential* or *counterfactual* outcome that individuals would have experienced if they had received a particular treatment or exposure value. This study focuses on counterfactual time‐to‐event outcomes by considering marginal structural hazard models (hazard‐MSM) or a discrete‐time analogue. The parameters of an MSM can be consistently estimated through various methods, including Inverse Probability of Treatment Weighting (IPTW) estimators, G‐computation, or doubly robust methods (Clare et al. 2019; Daniel et al. 2013; Hernán and Robins 2020; Gabriel et al. 2024; Robins et al. 2000; van der Laan and Gruber 2016). Despite being less robust, IPTW‐based MSMs have largely been adopted in the applied literature, especially in epidemiology and medicine, due to their simplicity in both implementation and interpretation (Clare et al. 2019). IPTW‐based MSMs require the correct specification of the exposure model conditional on confounders (i.e., the *weighting model*) and special attention to the identifiability assumptions of consistency, no unmeasured confounding, and positivity (Cole and Frangakis 2009; Cole and Hernán 2008; Hernán and Robins 2020; Williamson and Ravani 2017). This work focuses on the latter, which is often overlooked compared to confounding bias.

Positivity holds if, for any combination of the measured confounders occurring among individuals in the population, there is a nonzero (i.e., positive) probability of receiving every level of the exposure possible under the target treatment strategies to be compared (Cole and Hernán 2008; Hernán and Robins 2020). While less well‐recognized than bias due to incomplete control of confounding, violations of the positivity assumption can increase both the variance and bias of causal effect estimates (Léger et al. 2022; Petersen et al. 2012). Positivity violations can occur in two situations (Y. Zhu et al. 2021). *Strict* (or *theoretical*) violations occur when certain treatment levels are impossible for specific subgroups of subjects. For example, if a certain treatment a is never given to individuals with severe comorbidities, then the causal effect of a cannot be estimated for that subgroup; the analysis should therefore focus only on individuals without severe comorbidities. Even in the absence of structural zeros, *random* zeros may occur by chance due to small sample sizes or highly stratified data by numerous confounders. *Near* (or *practical*) violations refer to situations where the assignment to a specific treatment is always theoretically possible but is not (or rarely) observed in the data due to randomness. Sampling variability may indeed result in subjects having a near‐zero probability of being exposed (or unexposed) for certain combinations of covariate values. These situations are common in practical applications, particularly when the sample size is small, and they pose significant challenges for causal inference. While treatment remains technically possible within a subgroup, its rarity makes reliable estimation difficult, particularly when using IPTW. Empirical studies across various clinical areas have reported evidence of near‐positivity violations, often indicated by extremely large inverse probability weights resulting from very small treatment probabilities in some covariate strata. Examples include studies investigating the effect of methotrexate on mortality in rheumatoid arthritis patients (Fewell et al. 2004), the impact of initiating highly active antiretroviral therapy on changes in HIV‐1 RNA viral load in HIV‐infected individuals (Cole and Hernán 2008), the effect of the anticoagulant warfarin on the risk of gastrointestinal bleeding (Platt et al. 2012), and the impact of metformin on colon cancer recurrence among diabetic patients (Y. Zhu et al. 2021). Such extreme weights can destabilize the analysis and inflate variance. To address these challenges and stabilize the variance of estimates, weight truncation (WT) is often applied in IPTW to down‐weight observations in regions where near‐violations occur (Cole and Hernán 2008; Xiao et al. 2013; Y. Zhu et al. 2021). However, if applied inappropriately, this technique could result in excessive truncation, which may introduce bias into the estimates.

In the literature, research studies on positivity violations have been previously carried out in a pedagogical manner by using real data to illustrate how incorrect inference occurs in estimating MSMs when positivity is violated (Bembom and van der Laan 2007; Cole and Hernán 2008; Mortimer et al. 2005; Rudolph et al. 2022; Y. Zhu et al. 2021; A. Zhu et al. 2023). Findings across different studies agreed that positivity violations have a more severe impact on the IPTW estimator than other causal estimators. However, when using real data, disentangling the effect of positivity violations from other sources of bias is typically not possible: important confounders may be undetected or unmeasured and the fulfillment of the remaining assumptions underlying the IPTW estimator is generally difficult to ascertain. Moreover, real data do not allow us to design scenarios that could be of interest, such as studying performance under different sample sizes. To overcome these limitations, other investigations were conducted more systematically by setting up simulation studies (Léger et al. 2022; Neugebauer and van der Laan 2005; Naimi et al. 2011; Petersen et al. 2012; Wang et al. 2006). Results confirmed that under positivity violations IPTW estimator performs worse than other methods, becoming very unstable and exhibiting high variability. However, these studies were limited to assessing the causal effect of a treatment assigned either at a single time point or twice.

This study investigates the impact of near‐positivity violations on the performance of IPTW‐estimated MSMs in longitudinal survival contexts with time‐varying confounding using a simulation‐based approach. No systematic simulation studies currently exist in this framework, largely due to the challenges of simulating longitudinal survival data under conditions of both exposure‐affected time‐varying confounding and near‐positivity violations. To address this gap, two algorithms are proposed for simulating longitudinal data from hazard‐MSMs, accommodating near‐positivity violations, a time‐varying binary exposure, and a survival outcome. These methods build on the works of Havercroft and Didelez (2012) and Keogh et al. (2021). Two simulation studies analyzing cases of near‐positivity violations, where remaining unexposed is rare within certain levels of confounders, through various scenarios and WT strategies are performed.

This study aims to highlight the critical importance of carefully considering the positivity assumption in causal studies that utilize longitudinal observational data and IPTW. The final purpose is to warn against the risks of underestimating the assumption's significance or uncritically applying WT methods. This is fundamental given that the past several decades have seen an exponential growth in causal inference approaches and their applications to observational data (Hammerton and Munafò 2021; Mitra et al. 2022; Olier et al. 2023), including emerging areas such as target trial emulations (Hernán and Robins 2016; Hernán 2021), prediction modeling under hypothetical interventions (Keogh and van Geloven 2024; Lin et al. 2021; van Geloven et al. 2020), or causal machine learning (Feuerriegel et al. 2024; Moccia et al. 2024).

This study is organized as follows: Section 2 briefly recalls the notation, MSMs for survival outcomes, and IPTW. Section 3 explains the proposed mechanism to enforce positivity violations in algorithms to simulate longitudinal data from MSMs. Sections 4 and 5 present the two simulation studies. Sections 6 and 7 finally provide a set of practical recommendations and discuss the findings. Statistical analyses were performed in the R software environment (R Core Team 2023). Source code is available at https://github.com/mspreafico/PosViolMSM. A vignette illustrating how to use the developed code and algorithms in practice is provided in the Supporting Information.

## 2 MSMs for Potential Survival Outcomes

### 2.1 Notation

Let us consider a set of i=1,⋯,n subjects and a set of regular visits k=0,1,⋯,K performed at times $q_{0}<q_{1}<\cdots <q_{K}$ (assumed to be the same for everybody). Each subject undergoes each visit up until event time $T_{i}^{*}=\min\left(C_{i},T_{i}\right)$, that is, the earlier of the time of the actual event of interest $T_{i}$ and the administrative censoring time $C_{i}$. At each visit k, if $T_{i}\geq q_{k}$, a binary treatment status $A_{i,k}\in \left\{0,1\right\}$ (unexposed vs. exposed; control vs. treatment) and a set of time‐dependent covariates $L_{i,k}$ are observed. A bar over a time‐dependent variable indicates the history, that is, ${\bar{A}}_{i,k}=\left(A_{i,0},\cdots ,A_{i,k}\right)$ and ${\bar{L}}_{i,k}=\left(L_{i,0},\cdots ,L_{i,k}\right)$. Finally, the binary failure indicator process is denoted by $Y_{i,k+1}$, where $Y_{i,k+1}=1$ if subject i has failed (e.g., died) in period $\left(q_{k};q_{k+1}\right]$, that is, $q_{k}<T_{i}\leq q_{k+1}$, or $Y_{i,k+1}=0$ otherwise.

### 2.2 MSMs for Counterfactual Hazard Rates

Marginal structural hazards models (hazard‐MSMs) are a class of causal models that focus on *counterfactual* time‐to‐event variables (Hernán and Robins 2020; Hernán et al. 2000; Robins et al. 2000). These variables represent the time at which an event would have been observed had a patient been administered a specific exposure strategy $\bar{a}=\left(a_{0},a_{1},\cdots ,a_{K}\right)$ with $a_{k}\in \left\{0,1\right\}$ for all k. Vector $\bar{a}$ might differ from the actual treatment received ${\bar{A}}_{i}={\bar{A}}_{i,K}=\left(A_{i,0},\cdots ,A_{i,K}\right)$. The *counterfactual event time* that would be observed in a subject under complete exposure history $\bar{a}$ is denoted by $T^{\bar{a}}$. Hazard‐MSMs hence model the counterfactual hazard rate:

$$\lambda^{\bar{a}}\left(t\right)=\lim_{\Delta t\to 0}\frac{P(t\leq T^{\bar{a}}<t+\Delta t|T^{\bar{a}}\geq t)}{\Delta t}.$$

In case of discrete‐time hazard of failure, a *marginal structural logistic regression model* (*logit‐MSM*) can be assumed to model the counterfactual probability of failure in a single interval $\left(q_{k},q_{k+1}\right]$, given survival up to $q_{k}$. The *logit‐MSM* for the counterfactual hazard at visit k is defined as follows:

(1) $$\lambda_{k}^{\bar{a}}=\mathrm{Pr}\left(Y_{k+1}^{\bar{a}}=1|Y_{k}^{\bar{a}}=0\right)={\text{logit}}^{-1}\left[{\tilde{\gamma }}_{0}+g\left({\tilde{\gamma }}_{A};{\bar{a}}_{k}\right)\right],$$

where $\bar{a}$ is the complete treatment strategy, $Y_{k}^{\bar{a}}$ is the counterfactual event indicator at visit k, g(·) is a function (to be specified) of the treatment strategy history up to visit k (denoted by ${\bar{a}}_{k}$), and $\left({\tilde{\gamma }}_{0},{\tilde{\gamma }}_{A}\right)$ is the vector of log odds ratios, with ${\tilde{\gamma }}_{0}$ as the intercept. Depending on the desired information provided in g(·), the hazard at visit k can thus assume different forms (see Appendix A.1).

In the context of continuous‐time hazard, a *marginal structural Aalen's additive hazard model* (*Aalen‐MSM*) can be assumed to model the counterfactual hazard at time t given treatment history $\bar{a}$:

(2) $$\lambda^{\bar{a}}\left(t\right)={\tilde{\alpha }}_{0}\left(t\right)+g\left({\tilde{\alpha }}_{A}\left(t\right);{\bar{a}}_{\left\lfloor t\right\rfloor }\right)$$

where ${\tilde{\alpha }}_{0}\left(t\right)$ is the baseline hazard at time t, ${\bar{a}}_{\left\lfloor t\right\rfloor }$ denotes treatment pattern up to the most recent visit prior to time t (i.e., $\left\lfloor t\right\rfloor =\max_{k\leq t}k$), g(·) is a function (to be specified) of treatment pattern ${\bar{a}}_{\left\lfloor t\right\rfloor }$, and ${\tilde{\alpha }}_{A}\left(t\right)$ is the vector of coefficients at time t. Depending on the desired information provided in g(·), the hazard at time t can thus assume different forms (see Appendix A.1).

#### 2.2.1 Hazard‐Based Estimands and Marginal Survival Probabilities

The logit‐MSM (1) estimates log odds ratios ${\tilde{\gamma }}_{A}$ and the Aalen‐MSM (2) the cumulative regression coefficients $\int_{0}^{t}{\tilde{\alpha }}_{A}\left(s\right)ds$. Since hazard‐based estimands may not have a straightforward interpretation, estimates from the MSMs are typically transformed into estimates for an interpretable causal estimand (Didelez and Stensrud 2022; Hernán 2010; Keogh et al. 2021; Martinussen et al. 2020). One example is comparing the marginal survival probabilities at time t, that is, $S^{\bar{a}}\left(t\right)=\mathrm{Pr}\left(T^{\bar{a}}>t\right)$, for *always treated* $\bar{a}=1=\left(1,\cdots ,1\right)$ (i.e., sustained use of the treatment) versus *never treated* $\bar{a}=0=\left(0,\cdots ,0\right)$ (i.e., sustained nonuse of the treatment), or evaluating the marginal risk difference between them. The marginal survival probability at time t under treatment history $\bar{a}$ can be computed based on the different hazard forms:

i. for the logit‐MSMs in (1) is given by
(3) $$S^{\bar{a}}\left(t\right)=\prod_{k\leq t}\left(1-\lambda_{k}^{\bar{a}}\right)=\prod_{k\leq t}\left(1-{\text{logit}}^{-1}\left[{\tilde{\gamma }}_{0}+g\left({\tilde{\gamma }}_{A};{\bar{a}}_{k}\right)\right]\right);$$
ii. for the Aalen‐MSM in (2) is given by
(4) $$\begin{array}{ccc} S^{\bar{a}}\left(t\right) & = & \exp\left(-\int_{0}^{t}{\tilde{\alpha }}_{0}\left(s\right)\;ds-\int_{0}^{1}g\left({\tilde{\alpha }}_{A}\left(s\right);a_{0}\right)\;ds\right.\\ & & -\left.\int_{1}^{2}g\left({\tilde{\alpha }}_{A}\left(s\right);{\bar{a}}_{1}\right)\;ds-\cdots -\int_{\left\lfloor t\right\rfloor }^{t}g\left({\tilde{\alpha }}_{A}\left(s\right);{\bar{a}}_{\left\lfloor t\right\rfloor }\right)\;ds\right). \end{array}$$

### 2.3 Inverse Probability of Treatment Weighting

In the presence of confounders, and assuming there are no unmeasured confounders, MSMs can be estimated from the observed data by applying a technique called IPTW (Hernán and Robins 2020). IPTW involves weighting the contribution of each subject i by the inverse of the probability of receiving their actual exposure level given their confounding covariates. This process creates a pseudo‐population where the effects of time‐dependent confounding are balanced, so association in hazard regression models is causation (Hernán and Robins 2020). To optimize the variance estimation, stabilized (or standardized) weights are usually preferred (Hernán and Robins 2020; Hernán et al. 2000; Léger et al. 2022; Robins et al. 2000). The stabilized weight for subject i at time t is defined as

(5) $$sw_{i}\left(t\right)=\prod_{k=0}^{\left\lfloor t\right\rfloor }\frac{\mathrm{Pr}\left(A_{i,k}|{\bar{A}}_{i,k-1},T_{i}\geq q_{k}\right)}{\mathrm{Pr}\left(A_{i,k}|{\bar{A}}_{i,k-1},{\bar{L}}_{i,k},T_{i}\geq q_{k}\right)},$$

where $\left\lfloor t\right\rfloor =\max_{k\leq t}k$ is the largest visit‐time prior to t, and $A_{-1}$ is defined to be 0. These weights are well‐defined only when the denominator probabilities are strictly greater than zero, that is, for each visit k, if $\mathrm{Pr}\left({\bar{A}}_{i,k-1}={\bar{a}}_{k-1},{\bar{L}}_{i,k}={\bar{l}}_{k},T_{i}\geq q_{k}\right)\neq 0$, then

$$\begin{array}{ccc} & & \mathrm{Pr}\left(A_{i,k}=a|{\bar{L}}_{i,k}={\bar{l}}_{k},{\bar{A}}_{i,k-1}={\bar{a}}_{k-1},T_{i}\geq q_{k}\right)>0\\ & & \;\;\text{for all}\;a\in \{0,1\}. \end{array}$$

This means that, at each visit k, there is a nonzero (i.e., positive) probability of receiving every level of exposure $A_{i,k}$ for every combination of values of exposure and covariate histories ${\bar{A}}_{i,k-1}$ and ${\bar{L}}_{i,k}$ that occur among at‐risk individuals ($T_{i}\geq q_{k}$) in the population, which is what the positivity assumption guarantees (Cole and Hernán 2008; Hernán and Robins 2020).

Even when standardized, weights $sw_{i}\left(t\right)$ can largely inflate for a subject i concerned by near‐positivity violation: when the denominator probabilities are very close to zero, weights become extremely large. In such cases, the common approach is to consider truncated stabilized weights ${\tilde{sw}}_{i}\left(t\right)$ obtained by truncating the lowest and the highest estimations by the first and 99th (1–99) percentiles, or alternatively by narrower truncations, such as the 2.5–97.5, 5–95, or 10–90 percentiles (Cole and Hernán 2008; Xiao et al. 2013; Y. Zhu et al. 2021).

### 2.4 Simulating Longitudinal Survival Data From Marginal Structural Hazard Models

Even when positivity holds, simulating longitudinal data when the model of interest is a model for potential outcomes, as for MSMs, is generally not straightforward (Evans and Didelez 2024). The main challenges consist of (i) replicating the complex dynamics of time‐varying confounding; (ii) generating data in such a way that the model of interest is correctly specified; and (iii) in case of survival or other non‐collapsible models (Didelez and Stensrud 2022; Robinson and Jewell 1991), reconciling the MSM with the conditional model used in Monte Carlo studies to generate the data. For these reasons, only a few methods for simulating data from hazard‐MSMs have been published in the literature (Evans and Didelez 2024; Havercroft and Didelez 2012; Keogh et al. 2021; Seaman and Keogh 2024; Young et al. 2010; Young and Tchetgen Tchetgen 2014; Xiao et al. 2010). These methods impose restrictions on the data‐generating mechanisms to address the issues mentioned, allowing for accurate simulation of longitudinal data from prespecified hazard‐MSMs.

**FIGURE 1:**
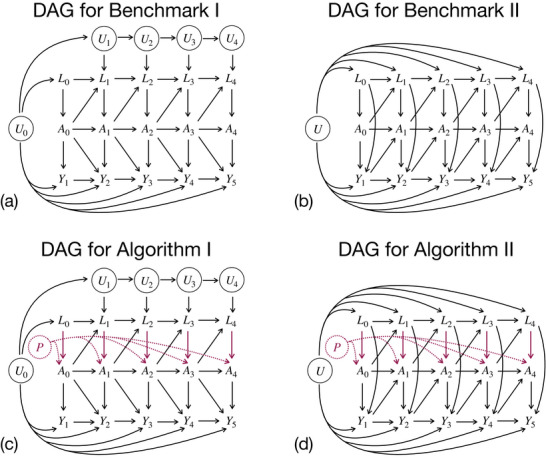
Top: Directed acyclic graphs (DAGs) illustrating the temporal causal relationships between variables in the data‐generating mechanisms proposed by (a) Havercroft and Didelez (2012) and (b) Keogh et al. (2021), that is, Benchmarks I and II. Bottom: DAGs illustrating the temporal causal relationships between variables in the proposed data‐generating mechanisms, that is, (c) Algorithm 2 and (d) Algorithm 3.

Among these, the approaches by Havercroft and Didelez (2012) and Keogh et al. (2021) present similar structures of the temporal causal relationships between variables. The directed acyclic graphs (DAGs) in the top panels of Figure 1 display for both cases the assumed data structure and inform which variables, measured at which time points, are confounders of the association between treatment at a given time point and the outcome. Both DAGs are illustrated in discrete time for a follow‐up with k=0,⋯,K visits. Variables are assumed to be constant between visits. By imagining splitting the time intervals between successive visits into smaller and smaller intervals, both structures approach the continuous‐time setting. For the current study, these two approaches are used as “truth” benchmarks for cases where longitudinal data generation from the desired hazard‐MSM has already been demonstrated and the positivity assumption is valid.

## 3 Simulating Longitudinal Survival Data With Random Positivity Violations

To impose near‐positivity violations within a data‐generating mechanism, certain treatment levels (e.g., exposure or non‐exposure in binary treatments) may become unobservable (though theoretically possible) for specific subgroups defined by confounders, due to randomness. Suppose the interest is in the subgroup of subjects presenting a poor health condition. Near‐positivity violations occur when the probability of remaining unexposed (or being exposed), given a poor health condition, is very close to zero (or approaches one). This happens when remaining unexposed to treatment at visit k is rarely observable for subjects in the poor health subgroup.

Let us define the subgroup of subjects presenting a poor health condition at visit k as determined by a range of values $I_{\tau }$ of the confounder $L_{i,k}$. Violations occurring by chance can be introduced in a data‐generating mechanism by considering (i) a latent individual propensity $P_{i}$ to exposure given a poor health condition, and (ii) an exposure cutoff π∈[0,1]. For each subject i, a random uniform variable $P_{i}$ is generated on the interval [0,1] and treatment assignment may be modified according to the exposure cutoff π. At each visit k, subjects in poor health with $P_{i}<\pi$ have a positive probability of either receiving exposure or remaining unexposed; in contrast, subjects in poor health with $P_{i}\geq \pi$ are deterministically assigned to exposure. This results in

(6) $$\begin{array}{c} \mathrm{Pr}\left(A_{i,k}=1|L_{i,k}\in I_{\tau },{\bar{L}}_{i,k-1},{\bar{A}}_{i,k-1},T_{i}\geq q_{k}\right)\\ =\left\{\begin{array}{cc} 1 & \;\text{if}\;P_{i}\geq \pi \\ p_{i,k}^{A}\in \left(0,1\right) & \;\text{if}\;P_{i}<\pi , \end{array}\right. \end{array}$$

when $\mathrm{Pr}\left({\bar{A}}_{i,k-1},{\bar{L}}_{i,k-1},L_{i,k}\in I_{\tau },T_{i}\geq q_{k}\right)\neq 0$. This leads to near‐violations of the positivity assumption because, within the poor health subgroup defined by individuals i with $L_{i,k}\in I_{\tau }$, both exposure and unexposure are theoretically possible. However, non‐exposure may be rarely observed due to the randomness in drawing the individual propensities for exposure, creating situations where noncompliance with positivity occurs more frequently than expected, especially for low values of π.

The parameter π represents the expected proportion of subjects for whom both exposure and non‐exposure are observable within each subgroup defined by the measured confounder, reflecting the *expected positivity support proportion*. Specifically,

- when π=1, all subjects have a nondeterministic probability $p_{i,k}^{A}$ of receiving treatment, ensuring that the positivity assumption is satisfied;
- when π=0, all subjects in poor health are deterministically assigned to receive treatment, representing a strict violation of the positivity assumption within the poor health group;
- when 0<π<1, approximately (1−π)×100% of subjects are expected to be deterministically assigned to exposure when they are in poor health. If unexposure is rarely observed within the remaining π proportion of subjects, the positivity assumption may be nearly violated within the poor health group due to limited support for the unexposed condition.

In other words, (1−π) can be interpreted as the expected proportion of subjects who deterministically contribute to violations of the positivity assumption within the poor health subgroup, while the remaining π proportion may contribute to near‐violations if the unexposed condition is rarely observed among them. Therefore, for a fixed $I_{\tau }$, the higher the cutoff π, the less severe the violation.

Given an algorithm to simulate longitudinal survival data from MSMs in the presence of time‐varying confounding, near‐positivity violations can be incorporated by using the pseudocode structure in panel Algorithm 1. The main advantage of imposing near‐positivity violations in an existing approach, where longitudinal data generation from the desired hazard‐MSM has already been confirmed, is the ability to directly examine the impact on IPTW estimators solely attributable to positivity violations, rather than other sources of bias. In this way, the original data‐generating mechanism can be considered as the “truth” or benchmark case where the positivity assumption holds.

ALGORITHM 1 **General pseudocode for random positivity violation.**

**Table:** 

Initialize parameters: $\left(I_{\tau },\pi ,\cdots \right)$
**for** i=1,⋯,n **do**
⋯
$P_{i}∼U\left(0,1\right)$ ▹ Draw the individual propensity
**for** k=0,⋯,K **do**
$L_{i,k}$ is assigned based on the generating algorithm
**if** $P_{i}\geq \pi$ and $L_{i,k}\in I_{\tau }$ **then**
exposure is assigned deterministically: $A_{i,k}$ = 1
**else**
exposure $A_{i,k}$ is assigned stochastically with $p_{i,k}^{A}\in \left(0,1\right)$ based on the generating algorithm
**end if**
⋯
**end for**
**end for**

## 4 Simulation Study I

### 4.1 Data Generation

The first algorithm proposed in this work is based on the data‐generating mechanism introduced by Havercroft and Didelez (2012) to simulate from a discrete‐time logit‐MSM. This mechanism is now briefly introduced and then extended by imposing positivity violations.

#### 4.1.1 Benchmark I in a Nutshell

Building upon the DAG in Figure 1a, Havercroft and Didelez (2012) proposed an algorithm to emulate longitudinal data from the Swiss HIV Cohort Study (Sterne et al. 2005). The authors considered a discrete‐time setting where visit times correspond to visit numbers, that is, $q_{k}=k$ for all k=0,⋯,K. The time‐dependent binary treatment process $A_{i,k}$ represents exposure to the highly active antiretroviral therapy (HAART) versus no treatment (unexposure). Once HAART has started for a subject i, it continues until failure or the end of the follow‐up period. The only measured time‐dependent confounder $L_{i,k}$ is the nonnegative CD4 cell count, measured in cells/μL. Variable $U_{i,k}$ represents the individual general latent health process, indicating a poor individual health status at visit k for values close to 0, or good health conditions for values close to 1. The latent process $U_{i,k}$ and the survival process $Y_{i,k+1}\in \left\{0,1\right\}$ are updated at each time point k, whereas CD4 cell count $L_{i,k}\geq 0$ and HAART exposure $A_{i,k}\in \left\{0,1\right\}$ are updated every κth time point, for a chosen κ, named checkup visits. Specifically, $U_{i,k}$, $L_{i,k}$, and $A_{i,k}$ are generated based on the latent general health process at visit k=0, $U_{i,0}$, which is also transformed to obtain $Y_{i,k+1}$, using the desired MSM survival function, once the actual treatment history is known.

Despite there being no direct arrow from $L_{i,k}$ to $Y_{i,k+1}$, the DAG (Figure 1a) exhibits time‐dependent confounding due to $U_{i,0}$ being a common ancestor of ${\bar{A}}_{i}$ via ${\bar{L}}_{i}$ and ${\bar{Y}}_{i}$. Moreover, $A_{i,k}$ is independent from ${\bar{U}}_{i,k}$ given $\left({\bar{L}}_{i,k},{\bar{A}}_{i,k-1}\right)$ and the vector ${\bar{L}}_{i}$ is sufficient to adjust for confounding. Based on this mechanism, the authors proposed an algorithm to correctly simulate data from the following discrete‐time logit‐MSM:

(7) $$\begin{array}{cc} \lambda_{k}^{\bar{a}} & ={\text{logit}}^{-1}\left[{\tilde{\gamma }}_{0}+{\tilde{\gamma }}_{A1}\cdot \left\{\left(1-a_{k}\right)k+a_{k}k^{*}\right\}\right.\\ & \;+\left.{\tilde{\gamma }}_{A2}\cdot a_{k}+{\tilde{\gamma }}_{A3}\cdot a_{k}\left(k-k^{*}\right)\right]\\ & ={\text{logit}}^{-1}\left[{\tilde{\gamma }}_{0}+{\tilde{\gamma }}_{A1}\cdot d_{1k}+{\tilde{\gamma }}_{A2}\cdot a_{k}+{\tilde{\gamma }}_{A3}\cdot d_{3k}\right], \end{array}$$

where $a_{k}$ is the binary treatment strategy at time k, $k^{*}$ is the treatment initiation time, $d_{1k}=\min\left\{k,k^{*}\right\}$ and $d_{3k}=\max\left\{k-k^{*},0\right\}$ represent the time elapsed before and after treatment initiation, respectively. Note that $g\left({\tilde{\gamma }}_{A};{\bar{a}}_{k}\right)={\tilde{\gamma }}_{A1}\cdot d_{1k}+{\tilde{\gamma }}_{A2}\cdot a_{k}+{\tilde{\gamma }}_{A3}\cdot d_{3k}$ in (7), reflecting that the hazard‐MSM depends on a summary of the treatment history rather than only on the current treatment. In particular, Havercroft and Didelez proved that the parameters $\left({\tilde{\gamma }}_{0},{\tilde{\gamma }}_{A1},{\tilde{\gamma }}_{A2},{\tilde{\gamma }}_{A3}\right)$ in the desired logit‐MSM (7) are collapsible with the conditional distribution parameters $\left(\gamma_{0},\gamma_{A1},\gamma_{A2},\gamma_{A3}\right)$ in the following conditional logit model:

(8) $$\begin{array}{ccc} \lambda_{i,k} & = & {\text{logit}}^{-1}\left[\gamma_{0}+\gamma_{A1}\cdot \left\{\left(1-A_{i,k}\right)k+A_{i,k}K_{i}^{*}\right\}\right.\\ & & +\left.\gamma_{A2}\cdot A_{i,k}+\gamma_{A3}\cdot A_{i,k}\left(k-K_{i}^{*}\right)\right], \end{array}$$

where $K_{i}^{*}$ is the individual treatment initiation time and $\lambda_{i,k}$ represents the individual probability of failure in the interval k<t≤k+1, conditional on survival up to visit k.

#### 4.1.2 Algorithm 2: Imposing Random Positivity Violations in Benchmark I

As illustrated in the DAG in Figure 1c, the first proposed algorithm to account for potential near‐positivity violations builds upon Benchmark I (Figure 1a) by incorporating two additional components.

i. First, a poor health subgroup identified by $I_{\tau }$ and acting on the purple path $L_{i,k}\to A_{i,k}$ must be defined. Since a CD4 count below 500 cells/μL indicates the patients' immune system may be weakened, making them susceptible to developing serious infections from viruses, bacteria, or fungi that typically do not cause problems in healthy individuals, it is reasonable to assume that subjects in a poor health condition at visit k are identified by $L_{i,k}<\tau$, where τ∈[0;500] cells/μL. This is equivalent to a nonnegative CD4 range of the form $I_{\tau }=\left[0,\tau \right)$, where the upper threshold τ has to be defined according to the simulation scenario. The higher the upper threshold τ, the wider $I_{\tau }$ and the more severe the violations.
ii. Then, the latent individual propensity for exposure $P_{i}∼U\left(0,1\right)$ directly acts on $A_{i,k}$. Subjects in poor health condition with propensity $P_{i}$ above the *exposure cutoff* π (to be defined according to the simulation scenario) are forced to start the treatment.

The procedure proposed below extends the algorithm by Havercroft and Didelez (2012) by incorporating the possibility of near‐positivity violations. For details regarding the chosen parameter values, please refer to their primary work.

Procedure
For each subject i=1,⋯,n, the simulation procedure with K discrete time points and checkups every κth visit is as follows:
  1. Generate the individual propensity to exposure: $P_{i}∼U\left(0,1\right)$.
  2. Generate the general latent health status at baseline: $U_{i,0}∼U\left(0,1\right)$.
  3. Generate the baseline CD4 as a transformation of $U_{i,0}$ by the inverse cumulative distribution function of Γ(3,154) distribution plus an error $\varepsilon_{i,0}∼N\left(0,20\right)$: $L_{i,0}=F_{\Gamma (3,154)}^{-1}\left(U_{i,0}\right)+\varepsilon_{i,0}$.
  4. If $P_{i}\geq \pi$ and $L_{i,0}<\tau$, the subject starts HAART and $A_{i,0}=1$. Otherwise, draw treatment decision $A_{i,0}∼Be\left(p_{i,0}^{A}\right)$ where $p_{i,0}^{A}={\text{logit}}^{-1}\left[-0.405-0.00405\cdot (L_{i,0}-500)\right]$. If $A_{i,0}=1$, set the treatment initiation time $K_{i}^{*}$ to 0.
  5. Compute the conditional individual hazard $\lambda_{i,0}$ for k=0 using (8). If $\lambda_{i,0}\geq U_{i,0}$, death has occurred in the interval (0,1] and set $Y_{i,1}=1$. Otherwise, the subject survived and set $Y_{i,1}=0$.
  For k=1,⋯,K, if the individual is still at risk:
  6. Draw $U_{i,k}=\min\left\{1,\max\left\{0,U_{i,k-1}+N\left(0,0.05\right)\right\}\right\}$ as a perturbation of $U_{i,k-1}$ restricted to [0,1].
  7. If k is not a checkup visit, CD4 cell counts are not updated and $L_{i,k}=L_{i,k-1}$. Otherwise, update the count as $L_{i,k}=\max\left\{0,L_{i,k-1}+150\cdot A_{i,k-1}+\varepsilon_{i,k}\right\}$, where the addition of 150 indicates the positive effect of exposure to HAART on CD4 count, and $\varepsilon_{i,k}∼N\left(100\left(U_{i,k}-2\right),50\right)$ is a Gaussian drift term implying that the worse is the general health condition $U_{i,k}$ (i.e., value closer to 0), the stronger the negative drift in CD4.
  8. If k is not a checkup visit, treatment is not updated and $A_{i,k}=A_{i,k-1}$. Otherwise, assign exposure
    a. *deterministically*: if $P_{i}\geq \pi$ and $L_{i,k}<\tau$ or if treatment has started at previous checkup ($A_{i,k-\kappa }=1$), patient i is exposed to HAART and $A_{i,k}=1$;
    b. *stochastically*: otherwise, draw treatment decision $A_{i,k}∼Be\left(p_{i,k}^{A}\right)$, where
$$\begin{array}{ccc} p_{i,k}^{A} & = & {\text{logit}}^{-1}\left[-0.405+0.0205\cdot k\right.\\ & & -\left.0.00405\cdot (L_{i,k}-500)\right]. \end{array}$$
      As in Benchmark I, the conditional distribution parameters have been set to calibrate the logistic function such that $\mathrm{Pr}(A_{•,0}=1|L_{•,0}=500)=0.4$, $\mathrm{Pr}(A_{•,0}=1|L_{•,0}=400)=0.5$, and $\mathrm{Pr}(A_{•,10}=1|L_{•,10}=500)=0.45$, where “•” represents a placeholder indicating that the statements apply to all possible values of the first subscript.
    If the subject starts the treatment at visit/time k, set the treatment initiation time $K_{i}^{*}$ equal to k.
  9. Compute $\lambda_{i,k}$, that is, the individual probability of failure in the interval (k;k+1] conditional on survival up to visit k, using (8). If $S_{i}\left(t\right)=\prod_{j=0}^{k}\left(1-\lambda_{i,j}\right)\leq 1-U_{i,0}$, the death has occurred in the interval (k;k+1] and $Y_{i,k+1}=1$. Otherwise, the subject remains at risk and $Y_{i,k+1}=0$.

The related pseudocode is provided in Appendix A.2. Note that when π=1 the positivity assumption always holds and this procedure corresponds to the data‐generating mechanism of Benchmark I. An example of a dataset simulated using Algorithm 2 is available in the vignette provided as Supporting Information.

### 4.2 Simulation Study Using Algorithm 2

#### 4.2.1 Methods and Estimands

Investigations are performed in several scenarios by considering different sample sizes (n= 50, 100, 250, 500, 1000), exposure cutoff values (π= 0.05, 0.1, 0.3, 0.5, 0.8, 1), WT strategies (NoWT, 1–99, 5–95, 10–90), and poor health subgroups $I_{\tau }=\left[0;\tau \right)$ with varying upper thresholds (τ= 0, 100, 200, 300, 400, 500 measured in cells/μL). The other parameters are set to be identical to those used by Havercroft and Didelez (2012) to consider their results as a benchmark for this analysis. Specifically, K=40 time points with checkups every (κ=5)th visit are considered, and the desired conditional distribution parameters in Equation (8) are $\left(\gamma_{0},\gamma_{A1},\gamma_{A2},\gamma_{A3}\right)=\left(-3,0.05,-1.5,0.1\right)$. In this way, the true values of the parameters in logit‐MSM (7) are $\left({\tilde{\gamma }}_{0}^{*},{\tilde{\gamma }}_{A1}^{*},{\tilde{\gamma }}_{A2}^{*},{\tilde{\gamma }}_{A3}^{*}\right)=\left(-3,0.05,-1.5,0.1\right)$.

For each scenario, B=1000 simulated datasets are generated. The logit‐MSM (7) is fitted to each simulated dataset through IPTW estimation using (truncated) stabilized weights. Weight components at each checkup visit (k=0,κ,2κ,⋯) are estimated by logistic regression models for the probability of treatment initiation, with numerator and denominator in (5) defined, respectively, as

$$\begin{array}{cc} & \mathrm{Pr}\left(A_{i,k}=1|{\bar{A}}_{i,k-1}=\bar{0},Y_{i,k-1}=0\right)={\text{logit}}^{-1}\left[\theta_{0}+\theta_{1}\cdot k\right]\;\text{and}\;\\ & \mathrm{Pr}\left(A_{i,k}=1|{\bar{A}}_{i,k-1}=\bar{0},{\bar{L}}_{i,k},Y_{i,k-1}=0\right)\\ & \;={\text{logit}}^{-1}\left[\theta_{0}+\theta_{1}\cdot k+\theta_{2}\cdot \left(L_{i,k}-500\right)\right]. \end{array}$$

In this way, since π≠0, the denominator model is correctly specified according to the data‐generating mechanism (Bryan 2004; Havercroft and Didelez 2012).

The estimands of interest are the regression coefficients $\left({\tilde{\gamma }}_{0},{\tilde{\gamma }}_{A}\right)$ and the marginal survival probabilities in Equation (3) for the *always treated* versus *never treated* regimens, where $g\left({\tilde{\gamma }}_{A};{\bar{a}}_{k}\right)={\tilde{\gamma }}_{A1}\cdot d_{1k}+{\tilde{\gamma }}_{A2}\cdot a_{k}+{\tilde{\gamma }}_{A3}\cdot d_{3k}$.

Section 4.2.2 presents the results across all scenarios. For each regression coefficient, estimated bias, empirical standard error (empSE), and root mean squared error (RMSE) (Morris et al. 2019) are considered as performance measures. Marginal survival curves are presented graphically by showing the mean estimated curves across the B=1000 repetitions. Note that simulation settings with π=1 and NoWT are equivalent to Benchmark I, regardless of τ (positivity always holds when π=1). In such cases, the analyses are based on correctly specified logit‐MSMs and correctly specified models for the weights, so the resulting estimates are expected to be approximately unbiased.

Section 4.2.3 presents the results of three specific scenarios with (n,π,τ)=(500,0.1,400) and different WT strategies (NoWT; WT 1–99; WT 5–95). For each scenario, the log‐transformed within‐dataset summary measures (i.e., mean, maximum, and minimum) of the estimated standardized IPTW weights over repetitions are shown along with the corresponding estimation errors for the regression coefficients and relative performance measures. Estimated marginal survival curves for each simulated dataset are presented graphically, along with the mean estimated curve across repetitions.

#### 4.2.2 Results Across all Scenarios

**FIGURE 2:**
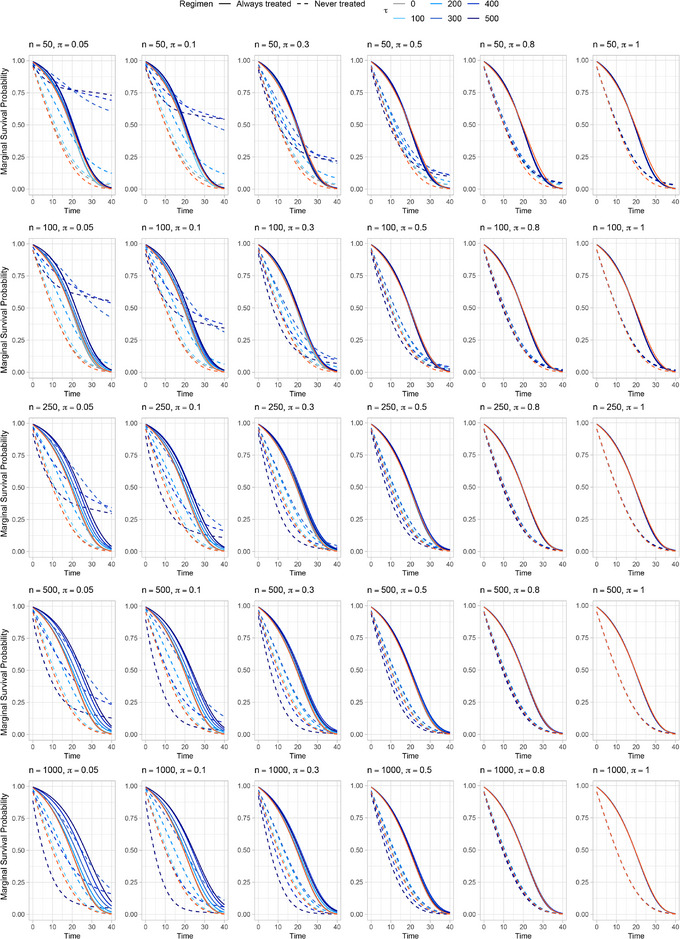
Marginal survival probability curves averaged across all the B=1000 repetitions for different settings without weight truncation (NoWT) of simulation study I. Each row refers to a different sample size n=50,100,250,500,1000. Each column refers to a different exposure cutoff π=0.05,0.1,0.3,0.5,0.8,1. Dashed lines refer to the *never treated* regimen, while solid ones refer to the *always treated* regimen. Curves are colored according to different values of rule‐threshold τ. True marginal survival curves are shown in orange.

Figure 2 shows the mean marginal survival curves, along with the true ones (in orange), in each simulated scenario without WT. Each line refers to a different τ value; the darker the line color, the more severe the violation (i.e., the bigger τ). Each row refers to a different sample size (n=50,100,250,500,1000), and each column refers to a different exposure cutoff (π=0.05,0.1,0.3,0.5,0.8,1). Similar behaviors are observed across different sample sizes as π varies: major deviations from true curves occur for small values of π, especially for the *never treated* group (dashed lines), while all scenarios eventually converged to the true values when no positivity violations are present (π=1). Contrary to expectations based on the true survival curves, for more severe violations (π=0.05,0.1; τ=300,400,500), the *never treated* group (dashed lines) exhibits notably better estimated marginal survival curves than the *always treated* group (solid lines), particularly in scenarios with small sample sizes.

This finding is supported by the magnitude and direction of the bias in the estimated regression coefficients $\left({\hat{\tilde{\gamma }}}_{0},{\hat{\tilde{\gamma }}}_{A1},{\hat{\tilde{\gamma }}}_{A2},{\hat{\tilde{\gamma }}}_{A3}\right)$, whose performance metrics (bias, empSE, RMSE) are reported in Supporting Information S1. Smaller sample sizes result in poorer performance across all regression coefficients. While increasing the sample size reduces bias and variability caused by finite sample limitations, estimation errors resulting from violations still persist, particularly for low values of π. Across all scenarios, as the severity of violations increases (i.e., the bigger τ and the lower π), the absolute bias, empSE, and RMSE grow substantially, particularly for ${\tilde{\gamma }}_{0}$ and ${\tilde{\gamma }}_{A2}$ (i.e., the intercept and the parameter most directly related to the effect of exposure). This outcome reflects how large weights resulting from near‐violations increase variability and reduce precision in IPTW‐based estimates (see Section 4.2.3 for further insights on this aspect). Consequently, since curves for *never treated* depend on $\left({\hat{\tilde{\gamma }}}_{0},{\hat{\tilde{\gamma }}}_{A1}\right)$, they generally show a greater deviation compared to those of *always treated*, which depend on $\left({\hat{\tilde{\gamma }}}_{0},{\hat{\tilde{\gamma }}}_{A2},{\hat{\tilde{\gamma }}}_{A3}\right)$—see Equation (1). Indeed, in the *always treated* the negative (positive) bias of ${\hat{\tilde{\gamma }}}_{0}$ can be balanced by the positive (negative) one of ${\hat{\tilde{\gamma }}}_{A2}$. This is not true for the *never treated*, so the deviation notably increases as near‐positivity violations become more concrete (i.e., as π decreases).

**FIGURE 3:**
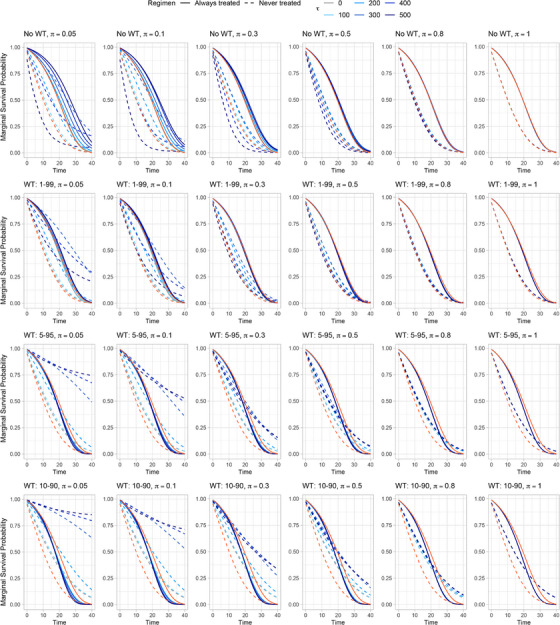
Marginal survival probability curves averaged across all the B=1000 repetitions for different settings with sample size n=1000 of simulation study I. Each row refers to a different weight truncation (WT) strategy: NoWT, 1–99, 5–95, 10‐99. Each column refers to a different exposure cutoff π=0.05,0.1,0.3,0.5,0.8,1. Dashed lines refer to the *never treated* regimen, while solid ones refer to the *always treated* regimen. Curves are colored according to different values of rule‐threshold τ. True marginal survival curves are shown in orange.

The paradox deviation of better survival for *never* treated becomes even more pronounced when any WT strategy is applied, as illustrated in Figure 3, where each row corresponds to a different WT strategy with a sample size of n=1000. The estimated mean curves are very close to the true ones for an expected positivity support proportion of 80% under NoWT, or even for 50% under 1–99 WT. However, under a more aggressive WT (5–95 or 10–90), the estimated curves deviate from the true ones, even at high exposure cutoffs. This pattern is again supported by the performance metrics of the estimated regression coefficients (see Supporting Information S1). Adopting a WT strategy does reduce variability and slightly decreases bias by truncating extreme weights, particularly for larger values of τ. However, further narrowing the truncation range (e.g., from WT 1–99 to WT 5–95 or 10–90) does not improve performance and may, in fact, degrade it. The following section offers a more in‐depth example illustrating this aspect.

#### 4.2.3 Focused Examination of WT in Selected Scenarios

To more closely examine the impact of applying or not applying WT in the presence of near‐violations, the results of three specific scenarios are presented below. Each scenario is defined by a sample size of n=500, an exposure cutoff of π=0.1, the poor health subgroup $I_{400}=\left[0;400\right)$, and one of three WT strategies: NoWT, WT 1–99, or WT 5–95.

**FIGURE 4:**
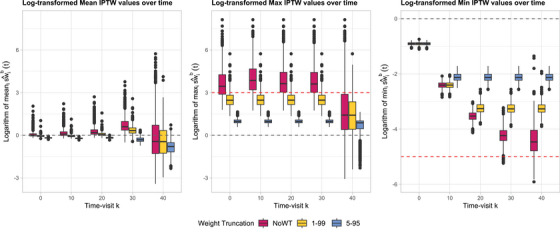
Boxplots of the logarithm of the within‐dataset mean (left panel), maximum (center panel), and minimum (right panel) of the estimated standardized IPTW weights over time, ${\hat{sw}}_{i}^{b}\left(t\right)$, computed across individuals in each dataset (b=1,⋯,1000) simulated using Algorithm 2 with a sample size of n=500, an exposure cutoff of π=0.1, the poor health subgroup $I_{400}=\left[0;400\right)$, and different WT strategies (magenta: NoWT; yellow: WT 1–99; blue: WT 5–95).

Figure 4 shows the boxplots of the logarithm of the within‐dataset mean (left panel), maximum (center panel), and minimum (right panel) of the estimated standardized IPTW weights, ${\hat{sw}}_{i}^{b}\left(t\right)$, computed across individuals (i=1,⋯,500) in each simulated dataset (b=1,⋯,1000). Each color refers to a different WT strategy (magenta: NoWT; yellow: WT 1–99; blue: WT 5–95). Under NoWT, several patterns indicate potential issues with weight stability. Deviations of log(mean) from 0 suggest shifts in the distribution of estimated stabilized weights across simulated datasets, driven by sampling variability, model sensitivity, or changes in covariate distributions over time, particularly as subjects die and exit the risk set. An observable increase in the range of log(mean) over time further indicates growing instability, likely due to deteriorating model performance in later periods. Values of log(max) greater than 3 reflect the presence of very large weights (e.g., ≥ 20), indicating limited covariate overlap and violations of the positivity assumption. The range of log(max) also increases over time, reflecting the greater influence of extreme weights as fewer subjects remain under observation. Although log(min) remains mostly above −5, suggesting that excessively small weights (e.g., <0.01) are rare, some variability is still observed, especially at later time points. This signals sensitivity to sparse covariate patterns or near‐deterministic treatment assignments among a shrinking subset of the population. These issues are most pronounced in the NoWT scenario (magenta), where extreme weights are not controlled. In contrast, truncation strategies such as WT 1–99 (yellow) or WT 5–95 (blue) effectively reduce the presence of extreme outliers, improving stability over time. However, high log(max) values under WT 1–99 still suggest potential positivity violations, while increasingly negative log(mean) under WT 5–95 indicates that some subgroups of the population have very low probabilities of receiving certain treatments, even after truncation.

**FIGURE 5:**
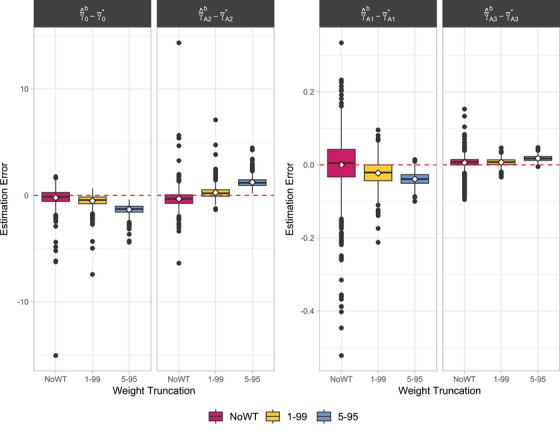
Boxplots of the estimation errors of the regression coefficients across the datasets simulated using Algorithm 2 with (n,π,τ)=(500,0.1,400) and three WT strategies (magenta: NoWT; yellow: WT 1–99; blue: WT 5–95). Each column refers to a different coefficient (${\tilde{\gamma }}_{0}$: first column; ${\tilde{\gamma }}_{A1}$: third column; ${\tilde{\gamma }}_{A2}$: second column; ${\tilde{\gamma }}_{A3}$: fourth column). The white diamonds represent the bias across repetitions. Note that the ranges of the *y*‐axes differ between panels.

Boxplots of the estimation errors of the regression coefficients across the simulated datasets for each scenario are shown in Figure 5. Mean estimated coefficients and relative performance measures in terms of bias, empSE, and RMSE are shown in Table 1. Results reflect how large weights resulting from near‐violations increase variability and reduce precision in IPTW‐based estimates. The error magnitude is notably higher for the intercept ${\tilde{\gamma }}_{0}$ and the coefficient most directly related to the effect of exposure ${\tilde{\gamma }}_{A2}$—see Equation (7)—compared to other coefficients, indicating that these parameters are more challenging to estimate accurately. The WT 1–99 strategy (yellow) effectively reduces the variability of the estimates, suggesting that this approach may stabilize the estimation process and improve precision compared to NoWT (magenta). In contrast, further narrowing to WT 5–95 (blue) introduces a clear bias in the estimates. This suggests that more aggressive WT strategies systematically shift the estimates away from the true values, compromising accuracy despite any gains in stability.

**TABLE 1:** Mean, bias, empirical standard error (empSE), and root mean squared error (RMSE) of the coefficient estimates for three different settings of simulation study I with sample size n=500, exposure cutoff of π=0.1, poor health subgroup $I_{400}=\left[0;400\right)$, and WT strategies ∈{NoWT, 1--99, 5--95}.

True coefficient	Weight Truncation	Mean	Bias	empSE	RMSE
${\tilde{\gamma }}_{0}^{*}=-3$	NoWT	−3.216	−0.216	0.881	0.907
	1–99	−3.520	−0.520	0.580	0.778
	5–95	−4.327	−1.327	0.463	1.405
${\tilde{\gamma }}_{A1}^{*}=0.05$	NoWT	0.050	−0.000	0.076	0.076
	1–99	0.028	−0.022	0.034	0.040
	5–95	0.011	−0.039	0.018	0.043
${\tilde{\gamma }}_{A2}^{*}=-1.5$	NoWT	−1.817	−0.317	0.919	0.972
	1–99	−1.235	0.265	0.581	0.638
	5–95	−0.269	1.231	0.466	1.316
${\tilde{\gamma }}_{A3}^{*}=0.1$	NoWT	0.106	0.006	0.019	0.020
	1–99	0.107	0.007	0.010	0.012
	5–95	0.118	0.018	0.008	0.020

**FIGURE 6:**
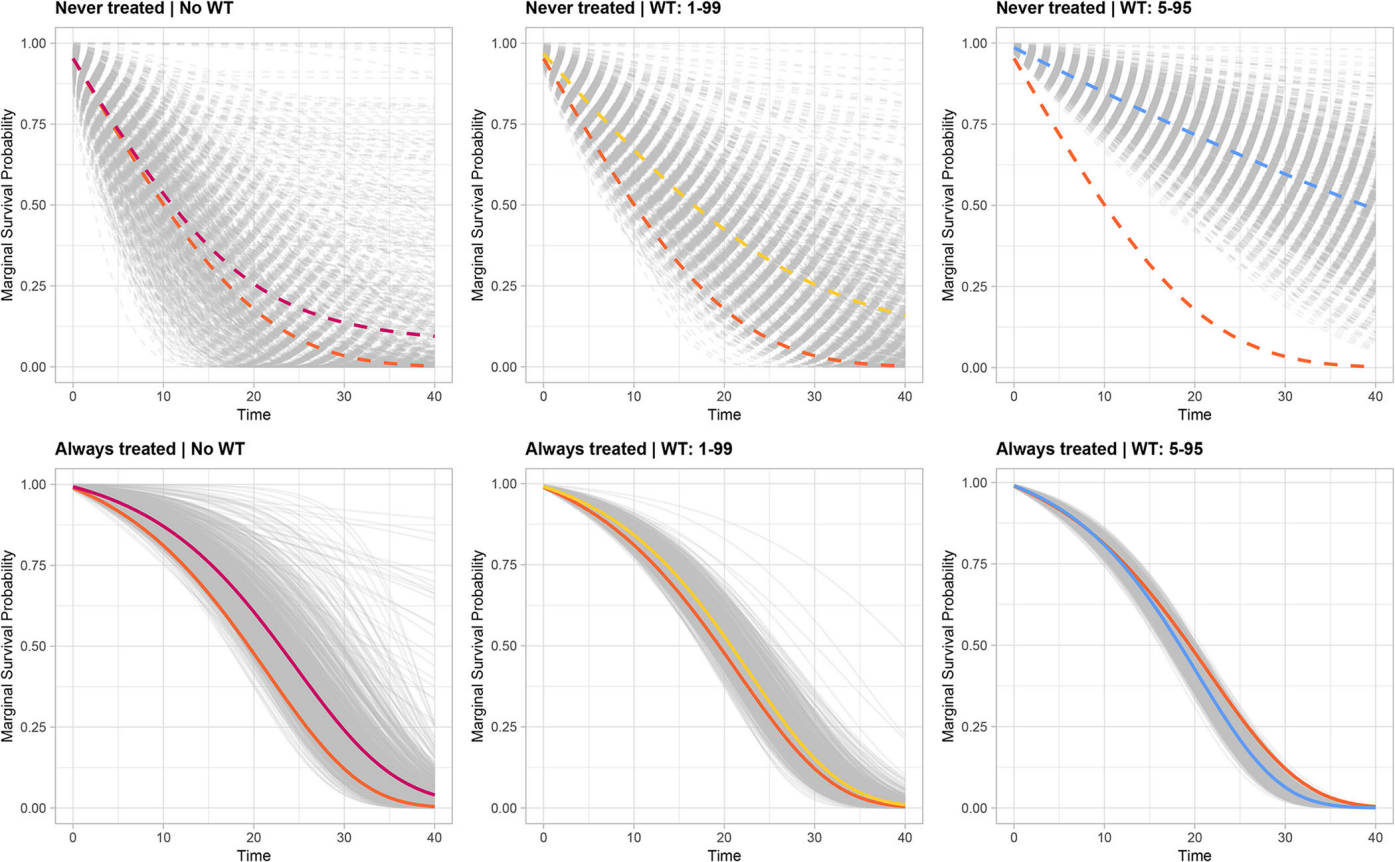
Estimated marginal survival curves for the *never treated* (top panels) and *always treated* (bottom panels) groups across B=1000 datasets simulated using Algorithm 2 with (n,π,τ)=(500,0.1,400) and different WT strategies (left panels: NoWT; middle panels: WT 1–99; right panels: WT 5–95). Each panel displays the true survival curve (in orange), the estimated curves for each dataset (in gray), and their mean (magenta: NoWT; yellow: WT 1–99; blue: WT 5–95).

This behavior is reflected in the estimates of marginal survival probabilities. Figure 6 shows the estimated marginal survival curves for the *never treated* (top panels) and *always treated* (bottom panels) groups across 1000 simulated datasets. Each panel displays the estimated curves for each dataset (in gray), their mean (in the same color as the WT strategy), and the true survival curve (in orange), under the three truncation strategies (left: NoWT; center: WT 1–99; right: WT 5–95). The plots highlight how highly variable IPTW weights, resulting from near‐violations of the positivity assumption, lead to substantial variability in the estimated survival curves, particularly for the *never treated* group. WT can reduce this variability but introduces bias, as it systematically excludes observations in the tails of the weight distribution. These observations often correspond to individuals in regions of the covariate space where the unexposed condition is rare but informative. Truncating these extreme weights disproportionately downweights such individuals, diminishing the representativeness of the weighted pseudo‐population. This creates a trade‐off: truncation improves stability but may compromise validity by altering the target estimand. As observed in Section 4.2.2, this issue becomes particularly concerning in settings with severe near‐violations of positivity, as extreme weights are more prevalent and the impact of excluding informative observations is amplified.

## 5 Simulation Study II

### 5.1 Data Generation

The second algorithm proposed in this work is based on the data‐generating mechanism introduced by Keogh et al. (2021) to simulate from an Aalen‐MSM. This mechanism is now briefly introduced and then extended by imposing positivity violations.

#### 5.1.1 Benchmark II in a Nutshell

Keogh et al. (2021) proposed a setting where, at each visit k=0,⋯,K, a binary treatment process $A_{i,k}\in \left\{0,1\right\}$ (control vs. treatment) and a time‐dependent biomarker $L_{i,k}\in R$ are observed for each subject i. The assumed data structure is illustrated in Figure 1b using a discrete‐time DAG setting where visit times correspond to visit numbers (i.e., $q_{k}=k$ ∀k) and $Y_{i,k+1}=I\left(k<T_{i}\leq k+1\right)$ is an indicator of whether the event $T_{i}$ occurs between visits k and k+1. As the time intervals become very small, the algorithm approaches the continuous time setting. The DAG also includes a baseline latent variable $U_{i}$, representing a subject‐specific unmeasured individual frailty, which has a direct effect on $L_{i,k}$ and $Y_{i,k+1}$, but not on $A_{i,k}$.

The DAG in Figure 1b exhibits time‐dependent confounding due to $L_{i,k}$ that predicts subsequent treatment use $A_{i,k}$, is affected by earlier treatment $A_{i,k-1}$, and affects the outcome $Y_{i,k+1}$ through pathways that are not just through subsequent treatment. Moreover, because $U_{i}$ is not a confounder of the association between the treatment and the outcome, the fact that it is unmeasured does not affect the ability to estimate causal effects of treatments. The authors demonstrated that using a conditional additive hazard of the form

(9) $$\lambda_{i}\left(t|{\bar{A}}_{i,\lfloor t\rfloor },{\bar{L}}_{i,\lfloor t\rfloor },U_{i}\right)=\alpha_{0}+\alpha_{A}\cdot A_{i,\lfloor t\rfloor }+\alpha_{L}\cdot L_{i,\lfloor t\rfloor }+\alpha_{U}\cdot U_{i},$$

their data‐generating mechanism correctly simulates data from the additive Aalen‐MSM of the form:

(10) $$\lambda^{\bar{a}}\left(t\right)={\tilde{\alpha }}_{0}\left(t\right)+\sum_{j=0}^{\left\lfloor t\right\rfloor }{\tilde{\alpha }}_{Aj}\left(t\right)\cdot a_{\left\lfloor t\right\rfloor -j},$$

that is an Aalen‐MSM including as treatment‐pattern $g\left({\tilde{\alpha }}_{A}\left(t\right);{\bar{a}}_{\left\lfloor t\right\rfloor }\right)$ the main effect terms at each visit (see Table A1). Researchers using this approach can only specify the parameters $\left(\alpha_{0},\alpha_{A},\alpha_{L},\alpha_{U}\right)$ of the conditional model (9). The true values of the cumulative regression coefficients $C_{0}\left(t\right)=\int_{0}^{t}{\tilde{\alpha }}_{0}\left(s\right)ds$ and $C_{Aj}\left(t\right)=\int_{0}^{t}{\tilde{\alpha }}_{Aj}\left(s\right)ds$ (j=0,⋯,4) of the Aalen‐MSM (10) must be computed using a simulation‐based approach, as detailed in Keogh et al. (2021).

**TABLE 2:** Mean, bias, empirical standard error (empSE), and root mean squared error (RMSE) of the cumulative coefficient estimates for three different settings of simulation study II with sample size n=500, exposure cutoff of π=0.05, poor health subgroup $I_{1}=\left(1;\infty \right)$, and WT strategies ∈{NoWT, 1--99, 5--95}.

Cum. Coefficient	Time	True	Weight Truncation	Mean	Bias	empSE	RMSE
$C_{0}\left(t\right)=\int_{0}^{t}{\tilde{\alpha }}_{0}\left(s\right)ds$	1	0.700	NoWT	0.693	−0.007	0.055	0.056
			1–99	0.694	−0.006	0.056	0.057
			5–95	0.688	−0.012	0.052	0.053
	2	1.408	NoWT	1.377	−0.031	0.176	0.179
			1–99	1.378	−0.030	0.124	0.127
			5–95	1.371	−0.037	0.094	0.101
	3	2.128	NoWT	2.069	−0.059	0.344	0.348
			1–99	2.059	−0.069	0.240	0.250
			5–95	2.050	−0.078	0.171	0.188
	4	2.863	NoWT	2.768	−0.095	0.478	0.487
			1–99	2.725	−0.138	0.388	0.412
			5–95	2.733	−0.130	0.285	0.314
	5	3.623	NoWT	3.457	−0.166	0.631	0.652
			1–99	3.424	−0.199	0.568	0.602
			5–95	3.403	−0.220	0.452	0.502
$C_{A0}\left(t\right)=\int_{0}^{t}{\tilde{\alpha }}_{A0}\left(s\right)ds$	1	−0.198	NoWT	−0.222	−0.024	0.192	0.194
			1–99	−0.213	−0.015	0.160	0.160
			5–95	−0.182	0.016	0.111	0.113
	2	−0.396	NoWT	−0.434	−0.038	0.371	0.372
			1–99	−0.417	−0.021	0.283	0.283
			5–95	−0.356	0.040	0.198	0.201
	3	−0.594	NoWT	−0.633	−0.039	0.555	0.556
			1–99	−0.613	−0.019	0.417	0.417
			5–95	−0.520	0.074	0.311	0.320
	4	−0.790	NoWT	−0.821	−0.031	0.747	0.747
			1–99	−0.767	0.023	0.610	0.610
			5–95	−0.676	0.114	0.464	0.477
	5	−0.987	NoWT	−0.974	0.013	0.969	0.969
			1–99	−0.934	0.053	0.832	0.834
			5–95	−0.819	0.168	0.660	0.681

**TABLE 3:** Mean, bias, empirical standard error (empSE), and root mean squared error (RMSE) of the cumulative coefficient estimates for three different settings of simulation study II with sample size n=500, exposure cutoff of π=0.05, poor health subgroup $I_{1}=\left(1;\infty \right)$, and WT strategies ∈{NoWT, 1--99, 5--95}.

Coefficient	Time	True	Weight Truncation	Mean	Bias	empSE	RMSE
$C_{A1}\left(t\right)=\int_{0}^{t}{\tilde{\alpha }}_{A1}\left(s\right)ds$	2	−0.098	NoWT	−0.048	0.050	0.344	0.348
			1–99	−0.044	0.054	0.241	0.247
			5–95	−0.045	0.053	0.163	0.172
	3	−0.195	NoWT	−0.109	0.086	0.571	0.577
			1–99	−0.074	0.121	0.421	0.438
			5–95	−0.075	0.120	0.285	0.310
	4	−0.291	NoWT	−0.149	0.142	0.795	0.807
			1–99	−0.117	0.174	0.618	0.642
			5–95	−0.117	0.174	0.468	0.499
	5	−0.386	NoWT	−0.156	0.230	1.013	1.038
			1–99	−0.171	0.215	0.856	0.883
			5–95	−0.135	0.251	0.696	0.740
$C_{A2}\left(t\right)=\int_{0}^{t}{\tilde{\alpha }}_{A2}\left(s\right)ds$	3	−0.077	NoWT	−0.016	0.061	0.444	0.448
			1–99	−0.045	0.032	0.355	0.356
			5–95	−0.033	0.044	0.246	0.250
	4	−0.153	NoWT	−0.071	0.082	0.644	0.649
			1–99	−0.050	0.103	0.590	0.599
			5–95	−0.040	0.113	0.439	0.453
	5	−0.228	NoWT	−0.093	0.135	0.921	0.930
			1–99	−0.041	0.187	0.875	0.894
			5–95	−0.053	0.175	0.669	0.691
$C_{A3}\left(t\right)=\int_{0}^{t}{\tilde{\alpha }}_{A3}\left(s\right)ds$	4	−0.060	NoWT	−0.015	0.045	0.525	0.526
			1–99	−0.019	0.041	0.482	0.483
			5–95	−0.005	0.055	0.357	0.361
	5	−0.121	NoWT	−0.052	0.069	0.812	0.814
			1–99	−0.045	0.076	0.770	0.773
			5–95	−0.022	0.099	0.620	0.628
$C_{A4}\left(t\right)=\int_{0}^{t}{\tilde{\alpha }}_{A4}\left(s\right)ds$	5	−0.047	NoWT	0.019	0.066	0.674	0.677
			1–99	−0.025	0.022	0.607	0.607
			5–95	−0.020	0.027	0.475	0.476

*Note:* For each j=1,2,3,4, the cumulative coefficient $C_{Aj}\left(t\right)$ is equal to 0 for t≤j.

**TABLE A1:** Examples of treatment‐pattern forms for function g(·) in Equations (1), (2), and (A1).

Form of treatment pattern	Logit‐MSM	Aalen‐MSM	Cox‐MSM
Function g(·)	$g\left({\tilde{\gamma }}_{A};{\bar{a}}_{k}\right)$ in (1)	$g\left({\tilde{\alpha }}_{A}\left(t\right);{\bar{a}}_{\left\lfloor t\right\rfloor }\right)$ in (2)	$g\left({\tilde{\beta }}_{A};{\bar{a}}_{\left\lfloor t\right\rfloor }\right)$ in (A1)
Current level of treatment	${\tilde{\gamma }}_{A}\cdot a_{k}$	${\tilde{\alpha }}_{A}\left(t\right)\cdot a_{\left\lfloor t\right\rfloor }$	${\tilde{\beta }}_{A}\cdot a_{\left\lfloor t\right\rfloor }$
Duration of treatment	${\tilde{\gamma }}_{A}\cdot \sum_{j=0}^{k}a_{k-j}$	${\tilde{\alpha }}_{A}\left(t\right)\cdot \sum_{j=0}^{\left\lfloor t\right\rfloor }a_{\left\lfloor t\right\rfloor -j}$	${\tilde{\beta }}_{A}\cdot \sum_{j=0}^{\left\lfloor t\right\rfloor }a_{\left\lfloor t\right\rfloor -j}$
Main effect terms at each visit	$\sum_{j=0}^{k}{\tilde{\gamma }}_{Aj}\cdot a_{k-j}$	$\sum_{j=0}^{\left\lfloor t\right\rfloor }{\tilde{\alpha }}_{Aj}\left(t\right)\cdot a_{\left\lfloor t\right\rfloor -j}$	$\sum_{j=0}^{\left\lfloor t\right\rfloor }{\tilde{\beta }}_{Aj}\cdot a_{\left\lfloor t\right\rfloor -j}$

Thanks to the collapsibility property of the Aalen's additive hazard model, the generating mechanism of Benchmark II includes the direct arrow from $L_{i,k}$ to $Y_{i,k+1}$, making it more realistic in practice compared to Benchmark I. Unlike Benchmark I, which is restricted to generating data closely matching the Swiss HIV Cohort Study (Sterne et al. 2005), Benchmark II can hence be applicable in more general contexts. However, its parameter values need to be carefully selected to ensure that the chance of obtaining a negative hazard, a common drawback in Aalen's model, is negligible.

#### 5.1.2 Algorithm 3: Imposing Random Positivity Violations in Benchmark II

Analogously to Algorithm 2, the second algorithm extends Benchmark II (Section 5.1.1) by imposing positivity violations randomly. As illustrated in the DAG in Figure 1d, compared to Benchmark II's structure in Figure 1b, two components are introduced.

i. The latent individual propensity for exposure $P_{i}∼U\left(0,1\right)$ directly acts on $A_{i,k}$: subjects in poor health condition with propensity $P_{i}$ above the *exposure cutoff* π (to be defined according to the simulation scenario) are always exposed.
ii. The poor health subgroup identified by $I_{\tau }$ acts on the purple path $L_{i,k}\to A_{i,k}$. In the framework of Keogh et al.'s procedure, the confounder $L_{i,k}$ represents a general biomarker with no direct real‐world interpretation, yet its higher values at time k correspond to an increased likelihood of exposure and an increased hazard. It is hence reasonable to assume that subjects with a poor health condition at time k are identified by $L_{i,k}>\tau$. This is equivalent to a range $I_{\tau }=\left(\tau ,\infty \right)$, where the lower threshold τ has to be defined according to the simulation scenario. Here, the lower the threshold τ, the wider the interval $I_{\tau }$ and the more severe the violation.

The proposed procedure extends Keogh et al.'s algorithm by incorporating the possibility for near‐positivity violations. For details regarding the chosen parameter values, please refer to their primary work.

Procedure
For each subject i=1,⋯,n, the simulation procedure with K+1 as administrative censoring time is as follows:
  1. Generate the individual propensity to exposure: $P_{i}∼U\left(0,1\right)$.
  2. Generate the individual frailty term: $U_{i}∼N\left(0,0.1\right)$.
  3. Generate the baseline biomarker as a transformation of $U_{i}$: $L_{i,0}∼N\left(U_{i},1\right)$.
  4. If $P_{i}\geq \pi$ and $L_{i,0}>\tau$, the subject is exposed to treatment and $A_{i,0}=1$. Otherwise, draw treatment decision $A_{i,0}∼Be\left(p_{i,0}^{A}\right)$, where $p_{i,0}^{A}={\text{logit}}^{-1}\left[-2+0.5\cdot L_{i,0}\right]$.
  5. Event times in the period 0<t<1 are generated by calculating $\Delta_{i}=-\log\left(\upsilon_{i,0}\right)/\lambda_{i}\left(t|A_{i,0},L_{i,0},U_{i}\right)$, where at numerator $\upsilon_{i,0}∼U\left(0,1\right)$ and the denominator is the individual conditional hazard in (9) with ⌊t⌋=0 and desired parameters. If $\Delta_{i}<1$, death occurred in the interval t∈(0,1): the event time is set to be $T_{i}=\Delta_{i}$, and the failure process is $Y_{i,1}=1$. Otherwise, subjects with $\Delta_{i}\geq 1$ remain at risk at time t=1 and set $Y_{i,1}=0$.
    For k=1,⋯,K, if the individual is still at risk:
  6. Update the biomarker value as: $L_{i,k}∼N\left(0.8\cdot L_{i,k-1}-A_{i,k-1}+0.1\cdot k+U_{i},1\right)$.
  7. Assign exposure
    a. *deterministically*: if $P_{i}\geq \pi$ and $L_{i,k}>\tau$, subject i is exposed to treatment and $A_{i,k}=1$;
    b. *stochastically*: otherwise, draw treatment decision $A_{i,k}∼Be\left(p_{i,k}^{A}\right)$ where
$$p_{i,k}^{A}={\text{logit}}^{-1}\left[-2+0.5\cdot L_{i,k}+A_{i,k}\right].$$
  8. Event times in the period k≤t<k+1 are generated by calculating $\Delta_{i}=-\frac{\log(\upsilon_{i,k})}{\lambda_{i}\left(t|{\bar{A}}_{i,k},{\bar{L}}_{i,k},U_{i}\right)}$, where $\upsilon_{i,k}∼U\left(0,1\right)$ and the denominator is the individual conditional hazard in (9) with ⌊t⌋=k and desired parameters. If $\Delta_{i}<1$, death occurred in the interval [k,k+1): the event time is set to be $T_{i}=k+\Delta_{i}$ and the failure process is $Y_{i,k+1}=1$. Otherwise, subjects with $\Delta_{i}\geq 1$ remain at risk at time k+1, that is, $Y_{i,k+1}=0$.
  Subjects who do not have an event time generated in the period 0<t<K+1 are administratively censored at time K+1.

The related pseudocode is provided in Appendix A.3. Note that when π=1 the positivity assumption always holds and this procedure corresponds to the data‐generating mechanism of Benchmark II. An example of a dataset simulated using Algorithm 3 is available in the vignette provided as Supporting Information.

### 5.2 Simulation Study Using Algorithm 3

#### 5.2.1 Methods and Estimands

Investigations are performed in several scenarios by considering different sample sizes (n= 50, 100, 250, 500, 1000), exposure cutoff values (π= 0, 0.05, 0.1, 0.3, 0.5, 0.8, 1), WT strategies (NoWT, 1–99, 5–95, 10–90), and poor health subgroups identified by intervals $I_{\tau }=\left(\tau ;\infty \right)$ with varying lower threshold τ. Since $L_{i,k}$ represents a general biomarker with no direct real‐world interpretation, the choice of possible values for τ relies on the distribution of the complete history of biomarker values generated using Benchmark II with 100,000 subjects. Specifically, the rounded values closest to the 80th, 90th, 95th, 99th, and 100th percentiles (i.e., τ=1,1.5,2,3,7) plus an extreme value outside the observed range (i.e, τ=10) are considered as possible lower thresholds. The other parameters are set to be identical to those considered by Keogh et al. (2021) in order to (i) have the same true values of the estimands of interest for the Aalen‐MSM (10) (see Tables 1 and 2 in Keogh et al. (2021), (ii) use their results as a benchmark for this analysis, and (iii) ensure that the probability of obtaining a negative hazard is negligible for Benchmark II. Specifically, K=4 time points with administrative censoring at K+1 are considered, and the conditional distribution parameters in Equation (9) were $\left(\alpha_{0},\alpha_{A},\alpha_{L},\alpha_{U}\right)=\left(0.7,-0.2,0.05,0.05\right)$.

For each scenario, B=1000 simulated datasets are generated. The Aalen‐MSM (10) is fitted to each simulated dataset through IPTW estimation using (truncated) stabilized weights. Weight components at time k are estimated by logistic regression models for the probability of being exposed at time k, with the numerator and denominator in (5) defined respectively as

$$\begin{array}{cc} \mathrm{Pr}\left(A_{i,k}=1|{\bar{A}}_{i,k-1},T_{i}\geq k\right) & ={\text{logit}}^{-1}\left[\theta_{0}+\theta_{1}\cdot A_{i,k-1}\right]\;\;\text{and}\;\\ \mathrm{Pr}\left(A_{i,k}\;=\;1|{\bar{A}}_{i,k-1},{\bar{L}}_{i,k},T_{i}\geq k\right)\; & =\;{\text{logit}}^{-1}\left[\theta_{0}+\theta_{1}\;\cdot A_{i,k-1}\;+\;\theta_{2}\;\cdot L_{i,k}\right]. \end{array}$$

In this way, since π≠0, the denominator model is correctly specified according to the data generation mechanism (Keogh et al. 2021).

The estimands of interest are the cumulative regression coefficients $C_{0}\left(t\right)=\int_{0}^{t}{\tilde{\alpha }}_{0}\left(s\right)ds$ and $C_{Aj}\left(t\right)=\int_{0}^{t}{\tilde{\alpha }}_{Aj}\left(s\right)ds$ (j=0,⋯,4), and the marginal survival probabilities in Equation (4) for the *always treated* and *never treated* regimens, where $g\left({\tilde{\alpha }}_{A}\left(t\right);{\bar{a}}_{\left\lfloor t\right\rfloor }\right)=\sum_{j=0}^{\left\lfloor t\right\rfloor }{\tilde{\alpha }}_{Aj}\left(t\right)\cdot a_{\left\lfloor t\right\rfloor -j}$.

Section 5.2.2 presents the results across all scenarios. For the cumulative regression coefficients, results are presented graphically by showing the performance (i.e., bias, empSE, and RMSE) measured at times t=1,2,3,4,5. For the marginal survival curves, the mean value of the estimates across repetitions is presented graphically across time points t=1,2,3,4,5. Note that simulation settings with π=1 and NoWT are equivalent to Benchmark II, regardless of τ (positivity always holds). In such cases, the analyses are based on correctly specified Aalen‐MSMs and correctly specified models for the weights, so the resulting estimates are expected to be approximately unbiased.

Section 5.2.3 presents the results of three specific scenarios with (n,π,τ)=(500,0.1,1) and different WT strategies (NoWT; WT 1–99; WT 5–95). For each scenario, the log‐transformed within‐dataset summary measures (i.e., mean, maximum, and minimum) of the estimated standardized IPTW weights over repetitions are shown along with the corresponding estimation errors for the cumulative regression coefficients and relative performance measures. Estimated marginal survival curves for each simulated dataset are presented graphically, along with the mean estimated curve across repetitions.

#### 5.2.2 Results Across All Scenarios

**FIGURE 7:**
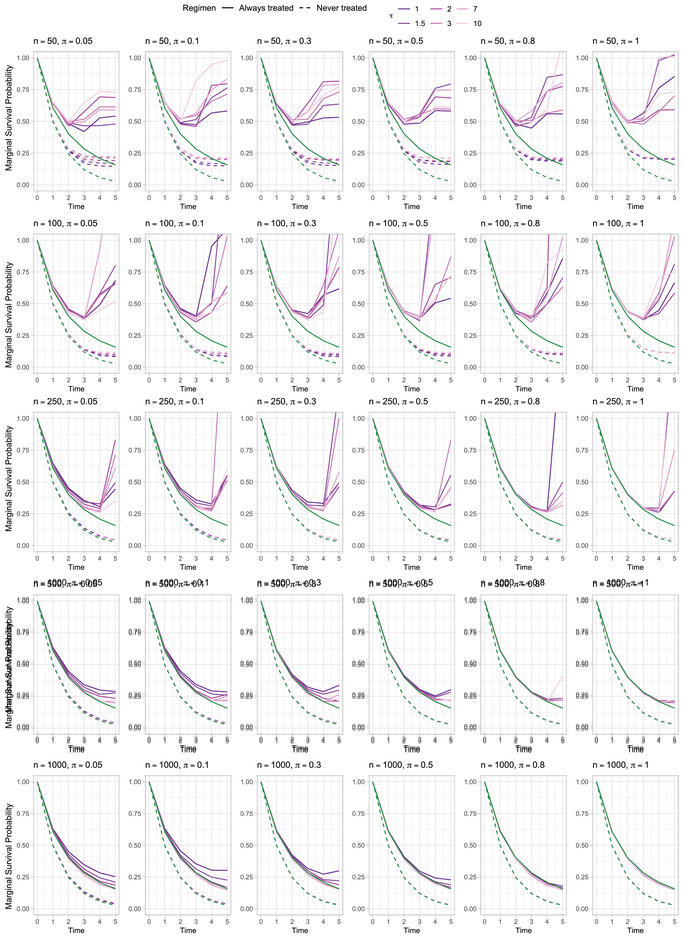
Marginal survival probability curves averaged across all the B=1000 repetitions for different settings without weight truncation (NoWT) of simulation study II. Each row refers to a different sample size n=50,100,250,500,1000. Each column refers to a different exposure cutoff π=0.05,0.1,0.3,0.5,0.8,1. Dashed lines refer to the *never treated* regimen, while solid ones refer to the *always treated* regimen. Curves are colored according to different values of rule‐threshold τ. True marginal survival curves are shown in green.

**FIGURE 8:**
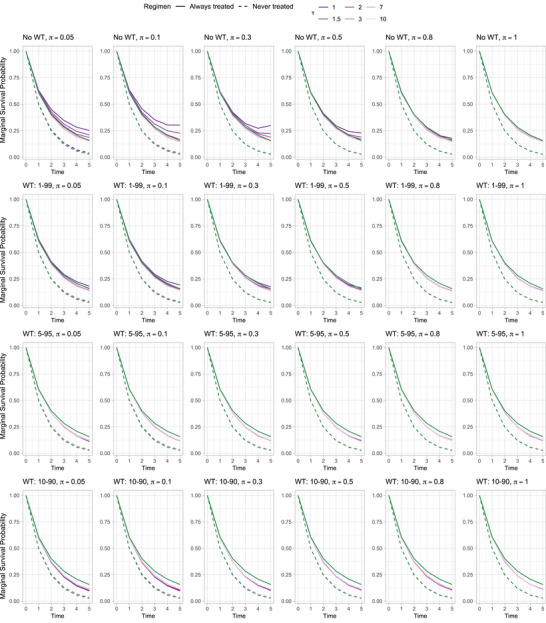
Marginal survival probability curves averaged across all the B=1000 repetitions for different settings with sample size n=1000 of simulation study II. Each row refers to a different weight truncation (WT) strategy: NoWT, 1–99, 5–95, 10–99. Each column refers to a different exposure cutoff π=0.05,0.1,0.3,0.5,0.8,1. Dashed lines refer to the *never treated* regimen, while solid ones refer to the *always treated* regimen. Curves are colored according to different values of rule‐threshold τ. True marginal survival curves are shown in green.

Figure 7 shows the mean marginal survival curves over times t=1,2,3,4,5 across repetitions estimated in the various scenarios without WT, along with the true ones (in green). Each line refers to a different τ value; the darker the line color, the more severe the violation (i.e., the smaller τ). Each row refers to a different sample size (n=50,100,250,500,1000), and each column to a different exposure cutoff (π=0.05,0.1,0.3,0.5,0.8,1). Even at high exposure cutoffs, scenarios with small sample sizes (n=50,100,250) heavily suffer from the main drawback of the additive hazard model, which does not restrict the hazard to be nonnegative. This determines survival probabilities for the *always treated* (solid lines) that wrongly increase over time. This issue is mitigated with bigger sample sizes (n=500,1000), where increasing the exposure cutoff π leads to estimated mean curves that closely align with the true ones, particularly when the expected positivity support proportion is 80% or higher. Adopting 1–99 WT (see Figure 8) improves performance compared to NoWT, with the estimated mean curves aligning with the true ones even at expected positivity support proportion as low as 30%. Nonetheless, further narrowing the WT range results in increased bias over time compared to 1–99 WT, suggesting that more aggressive truncation excludes important tail observations, reduces the representativeness of the pseudo‐population, and may introduce bias that outweighs the benefits of reduced variance.

These findings are supported by the performance metrics for the estimated cumulative coefficients over time (t=1,2,3,4,5) in each simulated scenario, as reported in Supporting Information S2. Indeed, the curves are derived from the cumulative coefficients—as in Equation (4)—whose estimates determine how closely the estimated mean curves match the true (green) ones. At each time point t=1,2,3,4,5, the curves for *never treated* (dashed lines) depend solely on ${\hat{C}}_{0}\left(t\right)$ (the cumulative intercept), while all cumulative coefficients contribute to estimating the curves for *always treated* (solid lines). The estimated bias for ${\hat{C}}_{0}\left(t\right)$ is negative and decreases over time, with minimal differences across π values for large sample sizes (n=500,1000). As a result, the estimated mean curves for *never treated* align with the true (green) curves for big sample sizes, exhibiting very low bias across time points. Conversely, summing the contributions of each cumulative coefficient results in higher bias for the *always treated*, particularly at later time points. Indeed, the results for the cumulative coefficients lead to similar conclusions: (i) performance worsens with time, (ii) adopting a WT strategy reduces the variability, and (iii) the estimated performance eventually converges to that observed under Benchmark II. Specifically, for the cumulative coefficient ${\hat{C}}_{A0}\left(t\right)$ related to the current main effect terms, smaller sample sizes exhibit worse performance, as increasing the sample size only mitigates the bias induced by finite sample issues. Across scenarios, bias, empSE, and RMSE increase with time. In general, the more severe the violation (i.e., low π, low τ), the higher empSE and RMSE. Adopting a WT strategy decreases empSE and RMSE, as extreme weights are truncated, especially for more severe violations. However, compared to WT 1–99, narrowing down the WT resulted in worse bias over time. Results eventually converge to be unbiased under NoWT, but a small bias still persists under 1–99 WT for t=4,5. In terms of variability, the empSE are comparable to the one estimated in Benchmark II, even when the expected positivity support proportion is 80%. The other treatment‐related cumulative coefficients, ${\hat{C}}_{Aj}\left(t\right)$ for j=1,2,3,4, exhibit similar patterns, with higher empSE and RMSE; however, unlike ${\hat{C}}_{A0}\left(t\right)$, no discernible relationship is found between the values of τ and the resulting bias.

#### 5.2.3 Focused Examination of WT in Selected Scenarios

Three specific scenarios are presented below to more closely examine the impact of WT on the estimation of the IPTW weights and the target estimands. Each scenario is generated B=1000 times and is defined by a sample size of n=500, an exposure cutoff of π=0.05, the poor health subgroup $I_{1}=\left(1;\infty \right)$, and one of three WT strategies: NoWT, WT 1–99, or WT 5–95.

**FIGURE 9:**
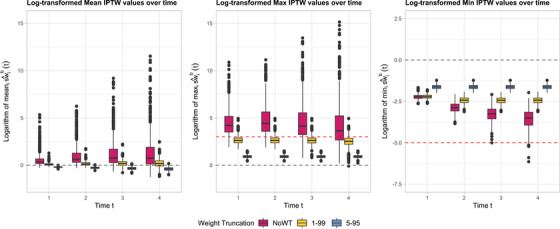
Boxplots of the logarithm of the within‐dataset mean (left panel), maximum (center panel), and minimum (right panel) of the estimated standardized IPTW weights over time, ${\hat{sw}}_{i}^{b}\left(t\right)$, computed across individuals in each dataset (b=1,⋯,1000) simulated using Algorithm 3 with a sample size of n=500, an exposure cutoff of π=0.05, the poor health subgroup $I_{1}=\left(1;\infty \right)$, and different WT strategies (magenta: NoWT; yellow: WT 1–99; blue: WT 5–95).

Figure 9 shows the boxplots of the logarithm of the within‐dataset mean (left panel), maximum (center panel), and minimum (right panel) of the estimated standardized IPTW weights, ${\hat{sw}}_{i}^{b}\left(t\right)$, computed across individuals (i=1,⋯,500) in each simulated dataset (b=1,⋯,1000). Several patterns indicate potential issues with weight stability. Under NoWT (magenta), deviations of log(mean) from 0 with an observable increase in the range over time suggest shifts in the weight distribution and growing instability. Values of log(max) greater than 3 indicate the presence of very large weights (e.g., ≥ 20), indicating limited covariate overlap and violations of the positivity assumption. The range of log(min) also increases over time, reflecting the greater influence of extreme weights as fewer subjects remain under observation. As expected, WT 1–99 (yellow) substantially decreases the range of log(mean) over time; however, high log(max) values greater than 3 are still observed, indicating possible violations of the positivity assumption. WT 5–95 (blue) more effectively limits extreme outliers; however, the increasingly negative log(mean) indicates that the mean of the standardized weights is drifting further from 1, suggesting potential positivity violations and raising concerns about bias.

**FIGURE 10:**
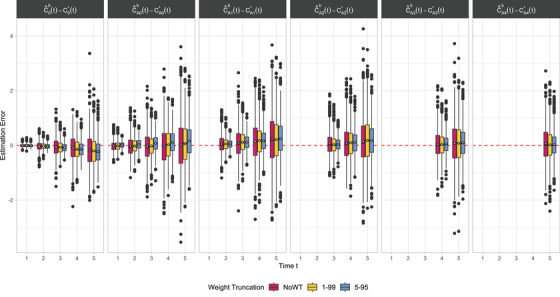
Boxplots of the estimation errors over time of the cumulative regression coefficients across the datasets simulated using Algorithm 3 with (n,π,τ)=(500,0.05,1) and three WT strategies (magenta: NoWT; yellow: WT 1–99; blue: WT 5–95). Each column refers to a different cumulative coefficient (first column: $C_{0}\left(t\right)=\int_{0}^{t}{\tilde{\alpha }}_{0}\left(s\right)ds$; columns from two to six: $C_{Aj}\left(t\right)=\int_{0}^{t}{\tilde{\alpha }}_{Aj}\left(s\right)ds$ with j=0,⋯,4, respectively). The white diamonds represent the bias across repetitions over time.

Figure 10 displays the boxplots of the estimation errors over time points t=1,⋯,5 of the cumulative regression coefficients $C_{0}\left(t\right)=\int_{0}^{t}{\tilde{\alpha }}_{0}\left(s\right)ds$ and $C_{Aj}\left(t\right)=\int_{0}^{t}{\tilde{\alpha }}_{Aj}\left(s\right)ds$ (j=0,⋯,4) across the simulated datasets for each scenario (magenta: NoWT; yellow: WT 1–99; blue: WT 5–95). Mean estimated coefficients and relative performance measures in terms of bias, empSE, and RMSE over time are shown in Tables 2 and 3. Results reflect how extreme weights resulting from near‐violations increase variability and reduce precision in IPTW‐based estimates, especially at later time points. By trimming extreme observations, WT strategies improve stability (lower empSE and RMSE) at the cost of increased bias, reflecting a trade‐off with estimate accuracy.

**FIGURE 11:**
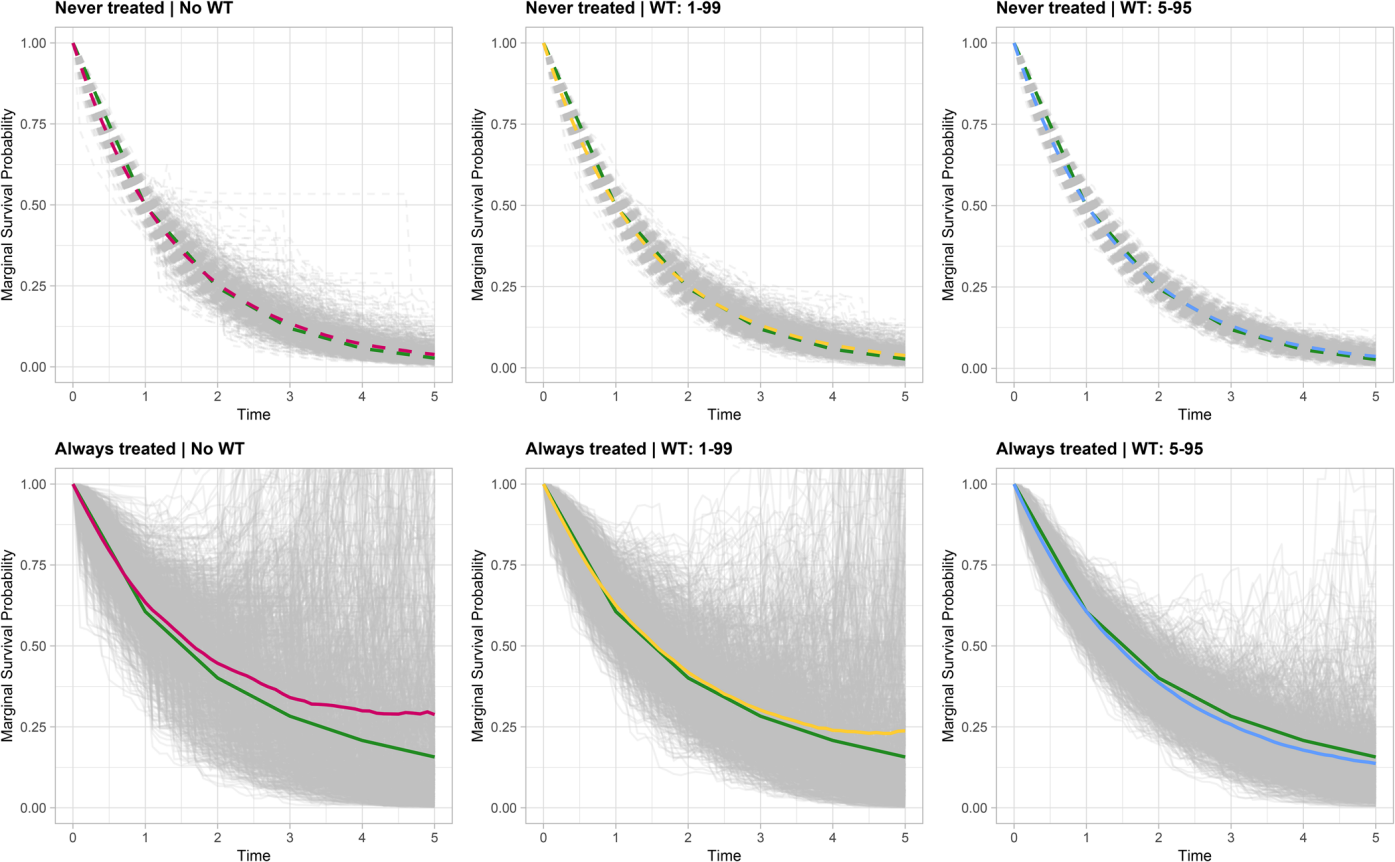
Estimated marginal survival curves for the *never treated* (top panels) and *always treated* (bottom panels) groups across B=1000 datasets simulated using Algorithm 3 with (n,π,τ)=(500,0.05,1) and different WT strategies (left panels: NoWT; middle panels: WT 1–99; right panels: WT 5–95). Each panel displays the true survival curve (in green), the estimated curves for each dataset (in gray), and their mean (magenta: NoWT; yellow: WT 1–99; blue: WT 5–95).

This pattern is evident in the estimated marginal survival probabilities across the simulated datasets presented in Figure 11 (gray lines). The plots highlight how extreme IPTW weights, resulting from near‐violations of the positivity assumption, lead to substantial variability in the estimated survival curves, particularly for the *always treated* group (bottom panels), which is more affected than the *never treated* group (top panels) by the inability of the additive hazards model to constrain the hazard function to be nonnegative. Compared to the NoWT strategy (left panels), WT 1–99 (middle panels) and WT 5–95 (right panels) help mitigate this variability, resulting in mean curves (colored lines) that more closely approximate the true survival curves (in green). However, the issue of individual non‐monotonic survival curves still persists.

## 6 Practical Tips for Applied Statisticians

The simulation studies discussed above provide the basis for deriving a set of practical recommendations for statisticians conducting real‐world studies in the presence of near‐positivity violations.

First, it is essential to conduct early diagnostic checks to identify potential positivity issues. This involves inspecting the distribution of IPTW weights, where extreme or highly variable weights, or stabilized weights with a mean far from one, may indicate limited treatment variation or model misspecification (Cole and Frangakis 2009; Cole and Hernán 2008). Routine use of summary statistics and diagnostic plots can help detect such problems. Additionally, quantitative measures—such as weighted standardized differences comparing covariate means or prevalences—and qualitative graphical methods should be employed to assess covariate balance between treatment groups in the weighted sample (Austin and Stuart 2015; Desai and Franklin 2019). Since the weights are time‐varying in longitudinal settings, these diagnostics should be performed separately at each time point, as the risk set can change over time.

Second, WT must be applied with caution. Truncating weights at high percentiles (e.g., 1–99) can substantially reduce estimator variance and improve stability. However, as shown in the simulation studies, more aggressive truncation usually leads to detrimental effects in terms of bias. Sensitivity analyses using a range of truncation thresholds are recommended to assess the robustness of the findings.

Third, analysts should consider alternative estimation strategies when near‐violations are suspected. Approaches like targeted maximum likelihood estimation, g‐computation, or doubly robust estimators can offer improved performance in these settings (Clare et al. 2019; Daniel et al. 2013; Léger et al. 2022; Petersen et al. 2012; Robins et al. 2000; van der Laan and Gruber 2016).

Fourth, when extreme sparsity in treatment–confounder combinations is observed, it may be appropriate to revise the causal estimand. Focusing on a subpopulation where sufficient support exists for both treatment arms can help preserve identifiability and yield more reliable estimates.

Finally, transparency in reporting is crucial. Researchers should clearly document any evidence of near‐positivity violations, the diagnostic tools employed, and the strategies adopted to address the issue, whether through WT strategies, alternative methods, or modified target populations. Such reporting not only strengthens the credibility of the analysis but also aids interpretability and reproducibility.

## 7 Discussion

Simulation studies play a key role in evaluating robustness to assumption violations, enabling the examination of various properties (Friedrich and Friede 2023; Morris et al. 2019). While existing literature on positivity violations in MSMs has largely focused on incorrect inferences using real data or simulations with exposure assigned at a single or two time points, this study fills the gap by presenting two simulation studies in realistic survival contexts involving a time‐varying binary treatment and a continuous time‐dependent confounder. Two distinct algorithms were proposed to simulate data from hazard‐MSMs and to account for potential near‐positivity violations, where remaining unexposed is rare within certain confounder levels. Systematic simulations were conducted to evaluate the impact of near‐positivity violations on the performance of target estimands obtained via IPTW under various scenarios and WT strategies.

Findings from both studies revealed a consistent trend: as the violation becomes more severe (i.e., low π), performance deteriorates. Increasing the sample size mitigates bias and variability due to finite sample size, but incorrect inference resulting from positivity violations persists. Even when $I_{\tau }$ is small, performance may still be poor due to the presence of extreme weights. Under NoWT, the higher the expected positivity support proportion π, the better the performance aligns with Benchmarks I and II. Adopting a WT strategy always reduces variability by truncating extreme weights, especially for wider $I_{\tau }$. WT 1–99 generally offers a better balance between reducing variability and maintaining accuracy than more aggressive truncations that may not improve bias. This suggests that bias becomes the more dominant factor when the positivity assumption is violated. The decision to adopt the 1–99 WT strategy in cases of near‐violations should carefully consider the bias‐variance trade‐off. For intermediate positivity support proportions (π=0.3,0.5), the 1–99 WT strategy generally outperformed NoWT. In contrast, for high values (π=0.8,1), NoWT was more effective.

Algorithms 2 and II (in the Supporting Information) proposed in this work were built on prior algorithms by Havercroft and Didelez (2012) and Keogh et al. (2021), respectively. The advantage of extending existing algorithms was threefold. First, the issue of non‐collapsibility (Didelez and Stensrud 2022; Robinson and Jewell 1991) between conditional and marginal models and the replication of complex confounding dynamics has already been overcome in the original studies. Second, by controlling the exposure‐confounder path and avoiding misspecification of the weighting model, the effect due to the imposed positivity violations was separated from other sources of bias. Third, the original Benchmarks I and II were used as the references for the expected true estimates when positivity is valid (i.e., for π=1).

While a direct comparison is not feasible as they pertain to different data‐generating mechanisms, Algorithm 2 generally exhibited poorer performance compared to Algorithm 3. This difference may stem from their distinct treatment decision mechanisms. Algorithm 2 requires continuous exposure until failure or censoring once treatment begins, whereas Algorithm 3 does not have such a requirement. This constraint limits the possible combinations of treatment‐covariate history in Algorithm 2, with a significant impact on the estimated coefficients, even though very few combinations are missing. Consequently, this influences the estimated mean survival curves, leading to incorrect survival probabilities for the *never treated* group. On the other hand, Algorithm 3 suffers, especially with small sample sizes, from the linear form of the Aalen‐MSM, which does not restrict the hazard to be nonnegative, resulting in unrealistic survival estimates.

This work has its limitations, which also open up intriguing possibilities for future research. Both studies focused on instances where violations occur within a single interval of the confounder variable and examined only a single continuous confounding variable. However, in real‐world scenarios, violations may span varied intervals, and multiple continuous/categorical confounding factors are typically present. This highlights interesting directions for extending the proposed algorithms, though adapting them to new contexts will require meticulous adjustments. Nonetheless, in their current form, Algorithms I and II developed in this study represent a valuable contribution to the literature. They could serve as data‐generating tools for systematic analyses, enabling (i) the comparison of different techniques for estimating causal effects from observational data under near‐positivity violations and (ii) the evaluation of potential new methods designed to address near‐positivity violations in a longitudinal‐treatment framework. Since current methods for detecting and addressing positivity violations are primarily tailored to point‐treatment settings (Danelian et al. 2023; Karavani et al. 2019; Traskin and Small 2011; A. Zhu et al. 2023; Zivich et al. 2024), developing methodologies specifically suited to a longitudinal framework presents a challenging direction for future research.

In summary, this study emphasizes the importance of carefully assessing positivity compliance to ensure robust and reliable causal inference in survival studies, while also highlighting the risks of underestimating it. By demonstrating the substantial impact of near‐positivity violations, it underscores the need for rigor in causal inference, particularly given the exponential growth of causal inference approaches and their applications to observational data. In practical analyses, researchers are strongly encouraged to examine group‐wise descriptives for the original and weighted populations, utilize bootstrap to quantify uncertainty in weights, and conduct sensitivity analysis for further insights (Austin and Stuart 2015; Cole and Hernán 2008; Desai and Franklin 2019). The causal effect of interest must be defined with consideration of positivity violations. While adopting a WT strategy may reduce variability, it should be approached with caution due to the potential risk of increased bias. Although IPTW‐based MSMs are widely used in applied studies for their simplicity in implementation and interpretation, analysts must remain vigilant about blindly accepting the positivity assumption, as doing so can lead to detrimental consequences. Finally, the two algorithms developed in this study also serve as valuable tools for generating data in future systematic analyses of novel causal inference methodologies.

## Funding

The author received no specific funding for this work. However, the author's position has been supported by KWF Kankerbestrijding (grant number 2023‐3 DEV / 15461).

## Conflicts of Interest

The author declares no conflicts of interest.

## Open Research Badges

This article has earned an Open Data badge for making publicly available the digitally‐shareable data necessary to reproduce the reported results. The data is available in the Supporting Information section.

This article has earned an open data badge “**Reproducible Research**” for making publicly available the code necessary to reproduce the reported results. The results reported in this article could fully be reproduced.

## Supporting information


**Supporting File 1:** bimj70093‐sup‐0001‐Datacode.zip.

**Supporting File 2:** bimj70093‐sup‐0002‐SuppMat.pdf.

**Supporting File 3:** bimj70093‐sup‐0003‐SuppMat.pdf.

## Data Availability

The data supporting the findings of this study can be generated using the code provided in the Supporting Information.

## A.1 Treatment‐Pattern Forms for Marginal Structural Hazard Models

As mentioned in Section 2.2, marginal structural hazards models (hazard‐MSMs) are a class of causal models for the *counterfactual event time* $T^{\bar{a}}$ that would be observed in a subject under complete exposure history $\bar{a}$ (Hernán and Robins 2020; Hernán et al. 2000; Robins et al. 2000). In the case of discrete‐time hazard of failure, the *logit‐MSM* form in (1) can be assumed to model the counterfactual probability of failure in a single interval $\left(q_{k},q_{k+1}\right]$, given survival up to $q_{k}$. In the context of continuous‐time hazard, the *Aalen‐MSM* form in (2) can be assumed to model the counterfactual hazard at time t given treatment history $\bar{a}$. An alternative often used is the *marginal structural Cox proportional hazard model* (*Cox‐MSM*) form, defined as

(A1)
$$\lambda^{\bar{a}}\left(t\right)=\lambda_{0}\left(t\right)\exp\left\{g\left({\tilde{\beta }}_{A};{\bar{a}}_{\left\lfloor t\right\rfloor }\right)\right\},$$

where $\lambda_{0}\left(t\right)$ is the baseline hazard function, ${\bar{a}}_{\left\lfloor t\right\rfloor }$ denotes treatment pattern up to the most recent visit prior to time t, g(·) is a function (to be specified) of treatment pattern ${\bar{a}}_{\left\lfloor t\right\rfloor }$, and ${\tilde{\beta }}_{A}$ is a vector of log hazard ratios.

In the hazard‐MSMs (1), (2), and (A1), the function g(·) combines information from the treatment pattern up to the most recent visit k prior to time t. Depending on the desired information provided in g(·), the hazard at time t (or visit k) can thus assume different forms. Table A1 shows examples of three forms of the treatment pattern function g(·), specifically: (i) the current level of treatment, (ii) the duration of treatment, or (iii) the history of treatment up to time t through the main effect terms for treatment at each visit. Any other desired form can be alternatively specified.

## A.2 Pseudocode of Algorithm 2

ALGORITHM 2 Pseudocode of Algorithm 2 introduced in Section 4.1.2. **Table:**

**Input parameters:**
n= sample size, τ= upper threshold of $I_{\tau }$, π= exposure cutoff
K= number of visits/time points, κ= checkup times,
$(\gamma_{0},\gamma_{A1},\gamma_{A2},\gamma_{A3})=$ conditional distribution parameters in Equation (8)
**Algorithm:**
**for** each subject i=1,⋯,n **do**
$P_{i}∼U\left(0,1\right)$▹ Individual propensity
$U_{i,0}∼U\left(0,1\right)$
$L_{i,0}=F_{\Gamma (3,154)}^{-1}\left(U_{i,0}\right)+\varepsilon_{i,0}$ where $\varepsilon_{i,0}∼N\left(0,20\right)$
**if** $P_{i}\geq \pi$ and $L_{i,0}<\tau$ **then**
$A_{i,0}=1$▹ Deterministic exposure assignment
**else**
$p_{i,0}^{A}={\text{logit}}^{-1}\left[-0.405-0.00405\cdot (L_{i,0}-500)\right]$▹ Stochastic exposure assignment
$A_{i,0}∼Be\left(p_{i,0}^{A}\right)$
**end if**
**if** $A_{i,0}=1$ **then** $K_{i}^{*}=0$ **end** **if**
$\lambda_{i,0}={\text{logit}}^{-1}\left[\gamma_{A0}+\gamma_{A2}\cdot A_{i,0}\right]$▹ Conditional hazard (8)
**if** $\lambda_{i,0}\geq U_{i,0}$ **then** $Y_{i,1}=1$ **else** $Y_{i,1}=0$ **end** **if**
k=1
**while** $Y_{i,k}=0$ and k≤K **do**
$U_{i,k}=\min\left\{1,\max\left\{0,U_{i,k-1}+\varepsilon_{i,k}\right\}\right\}$ where $\varepsilon_{i,k}∼N\left(0,0.05\right)$
**if** k mod κ≠0 **then**
$L_{i,k}=L_{i,k-1}$
$A_{i,k}=A_{i,k-1}$
**else**
$L_{i,k}=\max\left\{0,L_{i,k-1}+150\cdot A_{i,k-1}+\varepsilon_{i,k}\right\}$ where $\varepsilon_{i,k}∼N\left(100\left(U_{i,k}-2\right),50\right)$
**if** ($P_{i}\geq \pi$ and $L_{i,k}<\tau$) or $A_{i,k-\kappa }=1$ **then**
$A_{i,k}=1$▹ Deterministic exposure assignment
**else**
$p_{i,k}^{A}={\text{logit}}^{-1}\left[-0.405+0.0205\cdot k-0.00405\cdot (L_{i,k}-500)\right]$
▹ Stochastic exposure assignment
$A_{i,k}∼Be\left(p_{i,k}^{A}\right)$
**end if**
**if** $A_{i,k}=1$ and $A_{i,k-1}=0$ **then** $K_{i}^{*}=k$ **end** **if**
**end if**
$\lambda_{i,k}={\text{logit}}^{-1}\left[\gamma_{0}+\gamma_{A1}\cdot \left\{\left(1-A_{i,k}\right)k+A_{i,k}K_{i}^{*}\right\}+\gamma_{A2}\cdot A_{i,k}+\gamma_{A3}\cdot A_{i,k}\left(k-K_{i}^{*}\right)\right]$
▹ Conditional hazard (8)
**if** $\prod_{j=0}^{k}\left(1-\lambda_{i,j}\right)\leq 1-U_{i,0}$ **then** $Y_{i,k+1}=1$ **else** $Y_{i,k+1}=0$ **end** **if**
k=k+1
**end while**
**end for**

## A.3 Pseudocode of Algorithm 3

ALGORITHM 3 Pseudocode of Algorithm 3 introduced in Section 5.1.2. **Table:**

**Input parameters:**
n= sample size, τ= lower threshold of $I_{\tau }$, π= exposure cutoff
K= number of visits/time points
$(\alpha_{0},\alpha_{A},\alpha_{L},\alpha_{U})=$ desired true parameters in Equation (9)
**Algorithm:**
**for** each subject i=1,⋯,n **do**
$P_{i}∼U\left(0,1\right)$▹ Individual propensity
$U_{i,0}∼N\left(0,1\right)$
$L_{i,0}∼N\left(U_{i},1\right)$
**if** $P_{i}\geq \pi$ and $L_{i,0}>\tau$ **then**
$A_{i,0}=1$▹ Deterministic exposure assignment
**else**
$p_{i,0}^{A}={\text{logit}}^{-1}\left[-2+0.5\cdot L_{i,0}\right]$▹ Stochastic exposure assignment
$A_{i,0}∼Be\left(p_{i,0}^{A}\right)$
**end if**
$\lambda_{i}\left(t|A_{i,0},L_{i,0},U_{i}\right)=\alpha_{0}+\alpha_{A}\cdot A_{i,0}+\alpha_{L}\cdot L_{i,0}+\alpha_{U}\cdot U_{i}$▹ Conditional hazard (9)
$\Delta_{i}=-\log\left(\upsilon_{i,0}\right)/\lambda_{i}\left(t|A_{i,0},L_{i,0},U_{i}\right)$ where $\upsilon_{i,0}∼U\left(0,1\right)$
**if** $\Delta_{i}<1$ **then** $T_{i}=\Delta_{i}$ and $Y_{i,1}=1$ **else** $Y_{i,1}=0$ **end** **if**
k=1
**while** $Y_{i,k}=0$ and k≤K **do**
$L_{i,k}∼N\left(0.8\cdot L_{i,k-1}-A_{i,k-1}+0.1\cdot k+U_{i},1\right)$
**if** $P_{i}\geq \pi$ and $L_{i,k}>\tau$ **then**
$A_{i,k}=1$▹ Deterministic exposure assignment
**else**
$p_{i,k}^{A}={\text{logit}}^{-1}\left[-2+0.5\cdot L_{i,k}+A_{i,k}\right]$▹ Stochastic exposure assignment
$A_{i,k}∼Be\left(p_{i,k}^{A}\right)$
**end if**
$\lambda_{i}\left(t|{\bar{A}}_{i,k},{\bar{L}}_{i,k},U_{i}\right)=\alpha_{0}+\alpha_{A}\cdot A_{i,k}+\alpha_{L}\cdot L_{i,k}+\alpha_{U}\cdot U_{i}$▹ Conditional hazard (9)
$\Delta_{i}=-\log\left(\upsilon_{i,k}\right)/\lambda_{i}\left(t|A_{i,0},L_{i,0},U_{i}\right)$ where $\upsilon_{i,k}∼U\left(0,1\right)$
**if** $\Delta_{i}<1$ **then** $T_{i}=k+\Delta_{i}$ and $Y_{i,k+1}=1$ **else** $Y_{i,k+1}=0$ **end** **if**
k=k+1
**end while**
**if** is.null($T_{i}$) **then** $T_{i}=K+1$ **end** **if**▹ Administrative censoring at K+1
**end for**
