# Supplementary material for: Impact of Near‐Positivity Violations on IPTW‐Estimated Marginal Structural Survival Models With Time‐Dependent Confounding
Source: Biom J. 2025 Nov 3;67(6):e70093. doi: 10.1002/bimj.70093 (PMC12581517; doi:10.1002/bimj.70093)
Supplement: Supplementary file 2 — Supporting File 2: bimj70093‐sup‐0002‐SuppMat.pdf. [file BIMJ-67-e70093-s003.pdf]

Impact of near-positivity violations on IPTW-estimated marginal structural survival models with time-dependent confounding

Supplementary Material

Marta Spreafico

m.spreafico@math.leidenuniv.nl

S1 Simulation study I: additional results

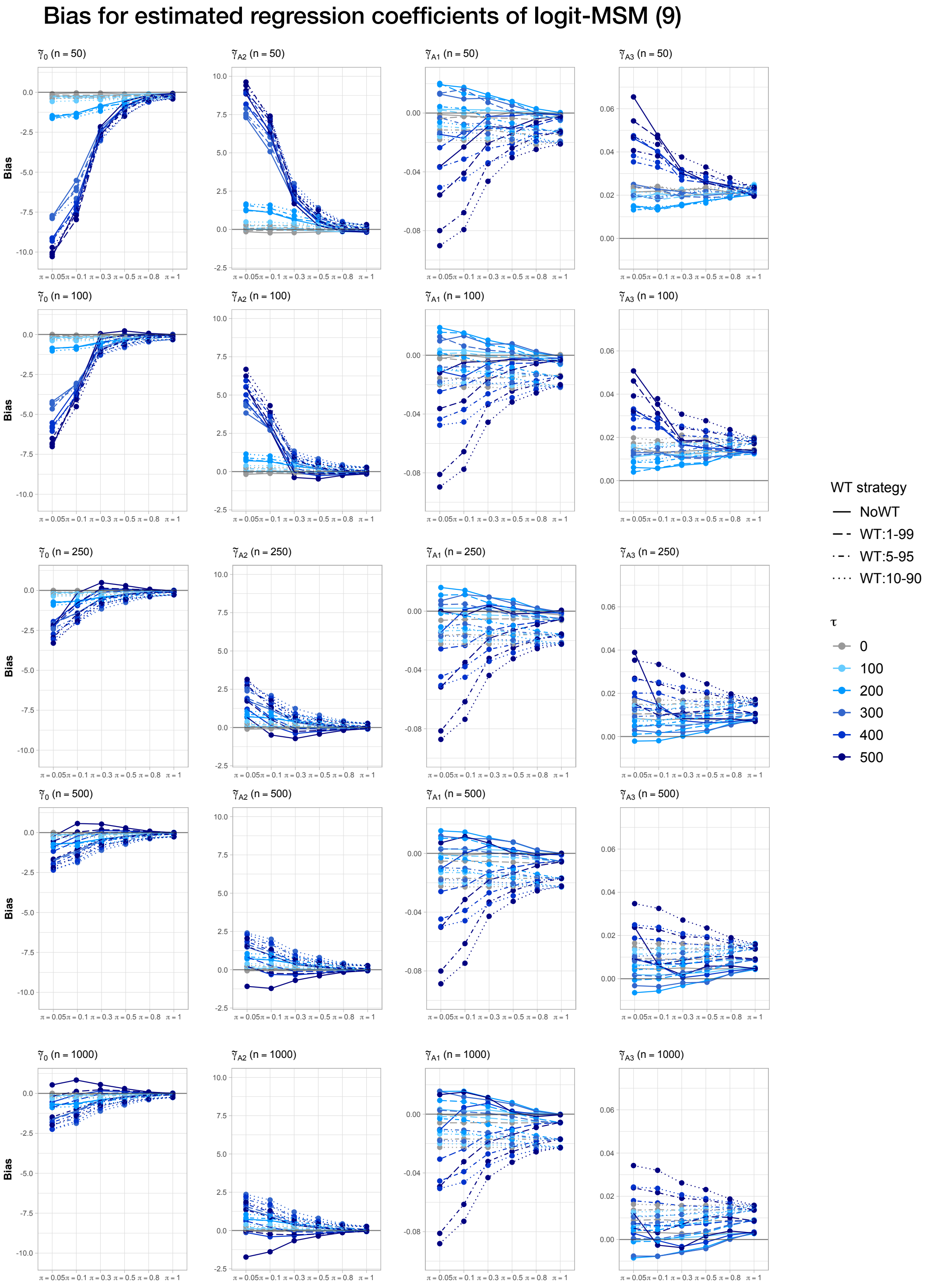

Figure S1: Bias of the coefficient estimates for the different setting of simulation study I. Each column refer to a different coefficient ($\tilde{\gamma}_0$: first column; $\tilde{\gamma}_{A1}$: third column; $\tilde{\gamma}_{A2}$: second column; $\tilde{\gamma}_{A3}$: fourth column). Each row refers to a different sample size $n = 50, 100, 250, 500, 1000$. The x-axes show the compliance-threshold values π. Different types of line refer to different weight truncation (WT) strategies (solid: No WT; long-dashed: 1-99 WT; dot-dashed: 5-95 WT; dotted: 10-90 WT). The colours refer to different values of the rule-threshold τ: the darker the colour, the more severe the violation (i.e., the higher τ). Note that the ranges of y-axes differ between panels.

EmpSE for estimated regression coefficients of logit-MSM (9)

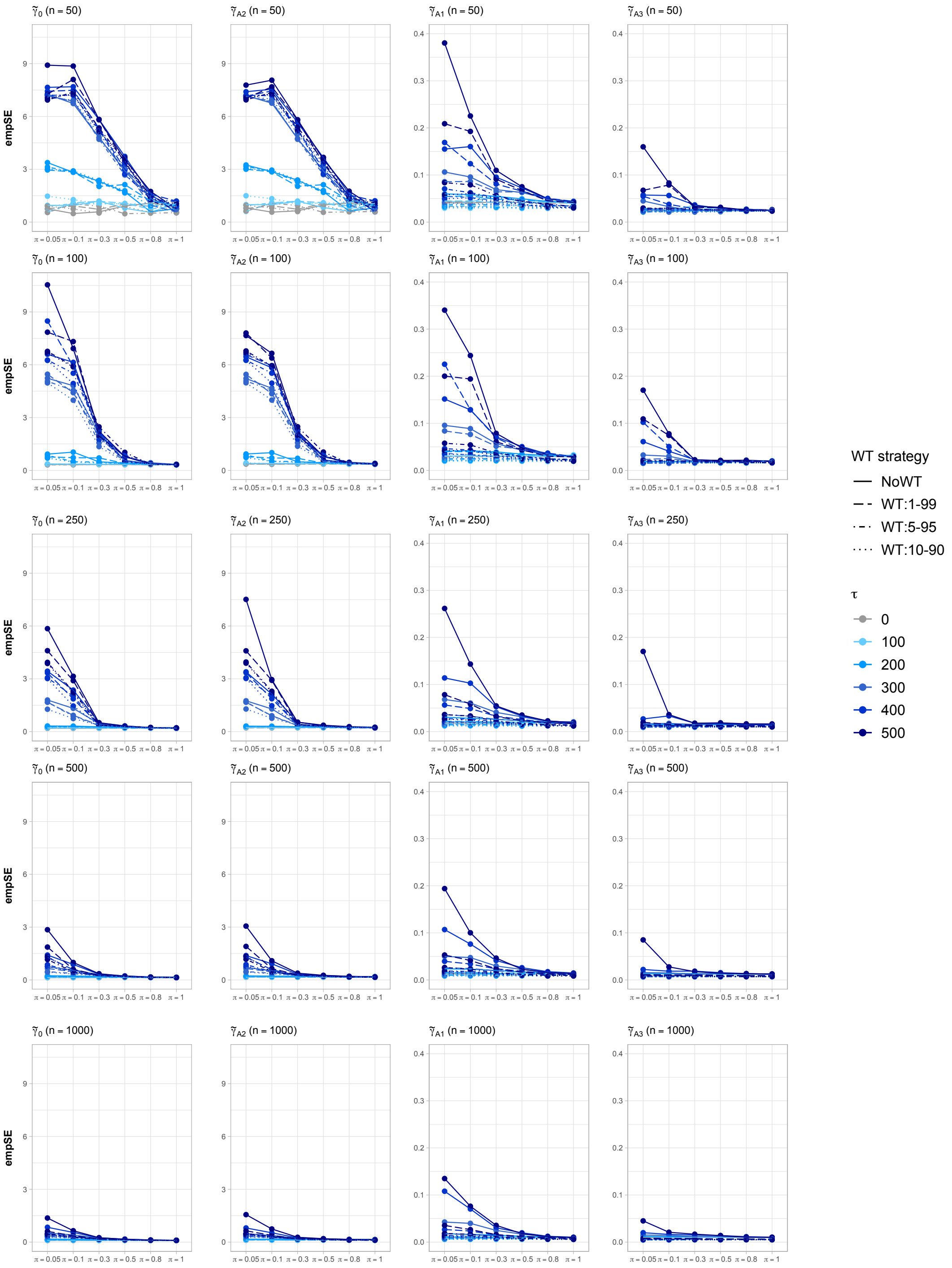

Figure S2: Empirical Standard Error (empSE) of the coefficient estimates for the different setting of simulation study I. Each column refer to a different coefficient ($\tilde{\gamma}_0$: first column; $\tilde{\gamma}_{A1}$: third column; $\tilde{\gamma}_{A2}$: second column; $\tilde{\gamma}_{A3}$: fourth column). Each row refers to a different sample size $n = 50, 100, 250, 500, 1000$. The x-axes show the compliance-threshold values π. Different types of line refer to different weight truncation (WT) strategies (solid: No WT; long-dashed: 1-99 WT; dot-dashed: 5-95 WT; dotted: 10-90 WT). The colours refer to different values of the rule-threshold τ: the darker the colour, the more severe the violation (i.e., the higher τ). Note that the ranges of y-axes differ between panels.

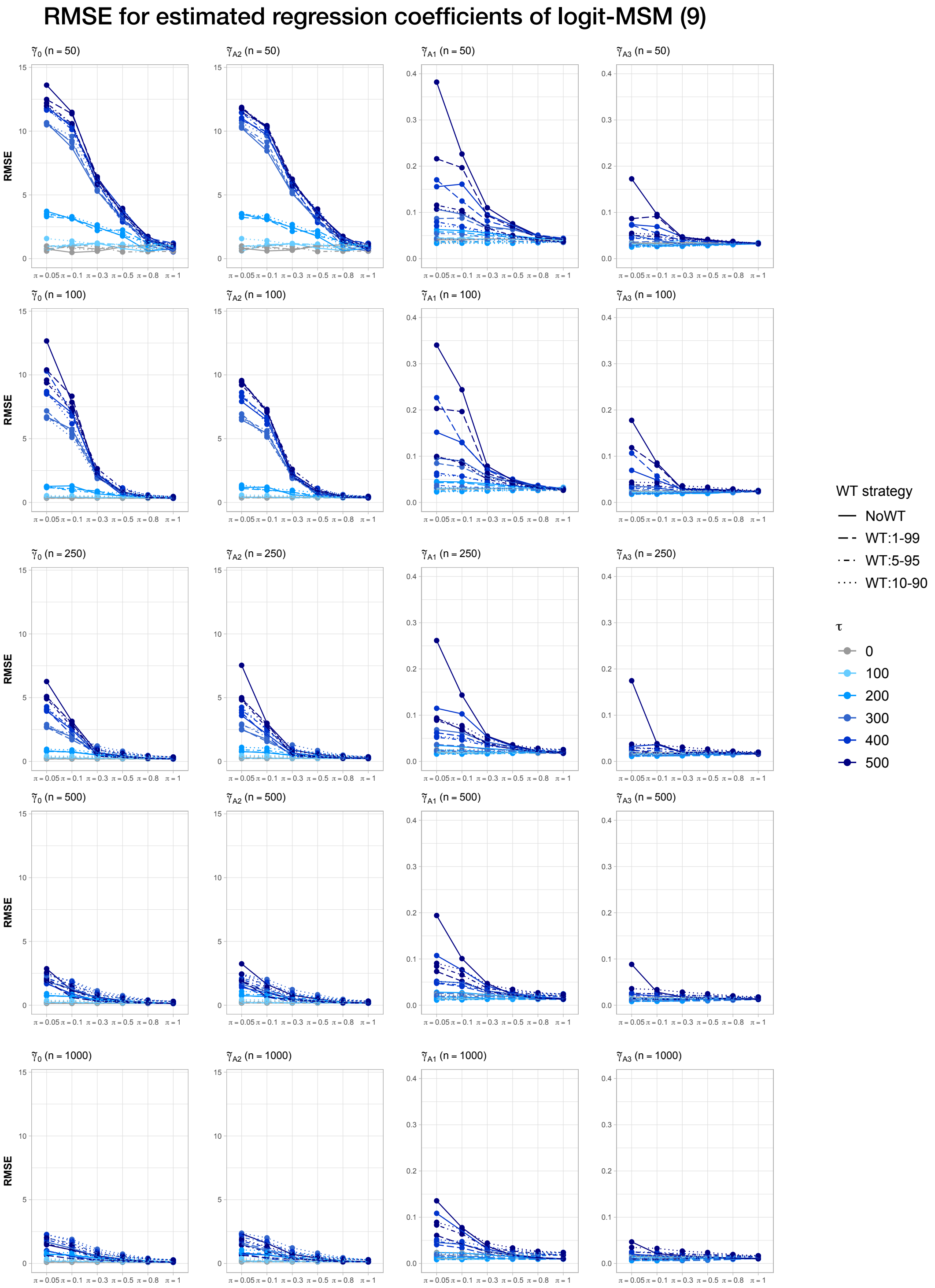

Figure S3: Root Mean Squared Error (RMSE) of the coefficient estimates for the different setting of simulation study I. Each column refer to a different coefficient ($\tilde{\gamma}_0$: first column; $\tilde{\gamma}_{A1}$: third column; $\tilde{\gamma}_{A2}$: second column; $\tilde{\gamma}_{A3}$: fourth column). Each row refers to a different sample size $n = 50, 100, 250, 500, 1000$. The x-axes show the compliance-threshold values π. Different types of line refer to different weight truncation (WT) strategies (solid: No WT; long-dashed: 1-99 WT; dot-dashed: 5-95 WT; dotted: 10-90 WT). The colours refer to different values of the rule-threshold τ: the darker the colour, the more severe the violation (i.e., the higher τ). Note that the ranges of y-axes differ between panels.

S2 Simulation study II: additional results

S2.1 Bias

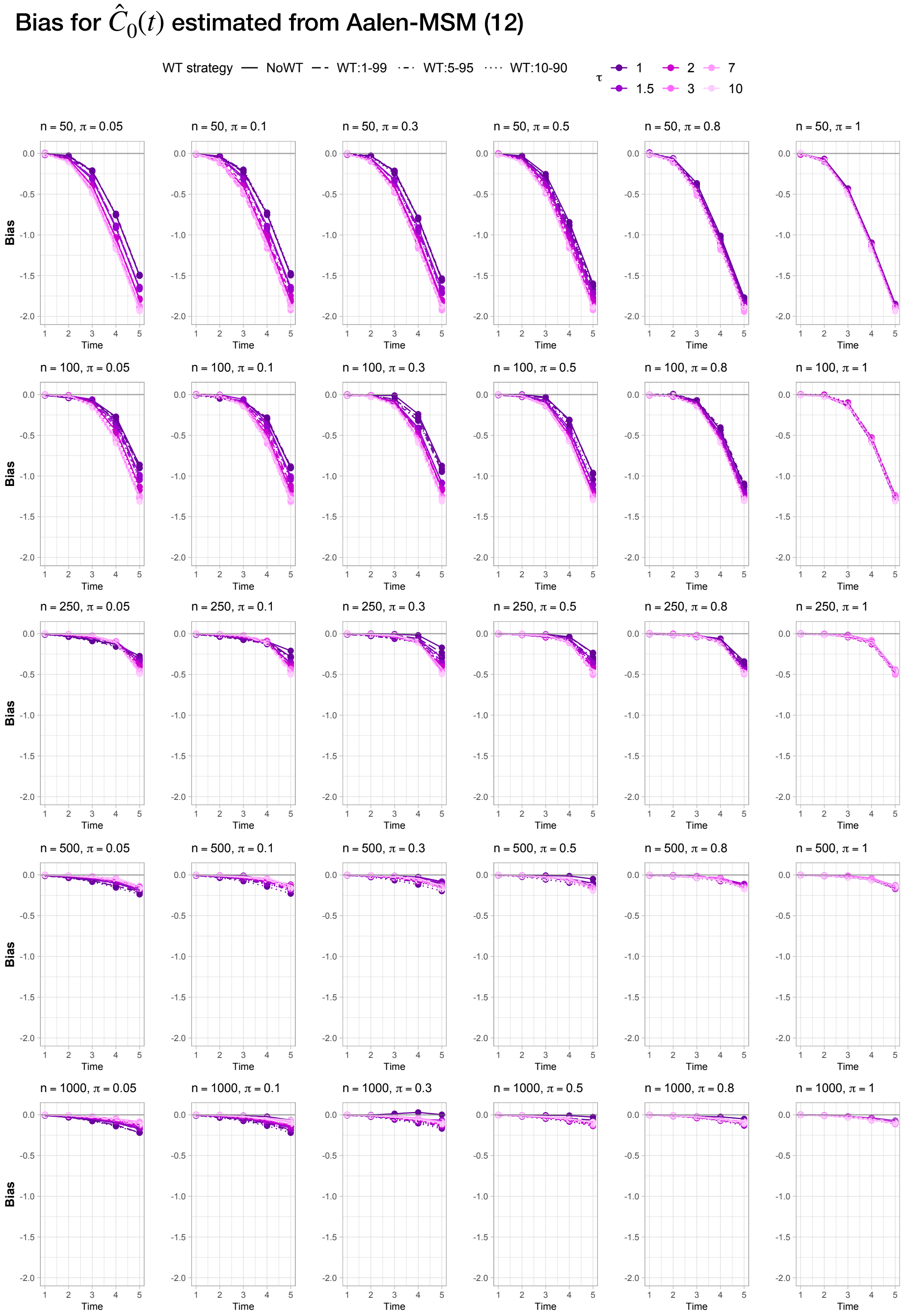

Figure S4: Bias of the estimates for the cumulative coefficient $C_0(t) = \int_0^t \tilde{\alpha}_0(s)ds$ at time points $t = 1, \ldots, 5$ for the different setting of simulation study II. Each row refers to a different sample size $n = 50, 100, 250, 500, 1000$. Each column refers to a different exposure cut-off $\pi = 0.05, 0.1, 0.3, 0.5, 0.8, 1$. Different types of line refer to different weight truncation (WT) strategies (solid: No WT; long-dashed: 1-99 WT; dot-dashed: 5-95 WT; dotted: 10-90 WT). The colours refer to different values of the rule-threshold τ: the darker the colour, the more severe the violation (i.e., the lower τ).

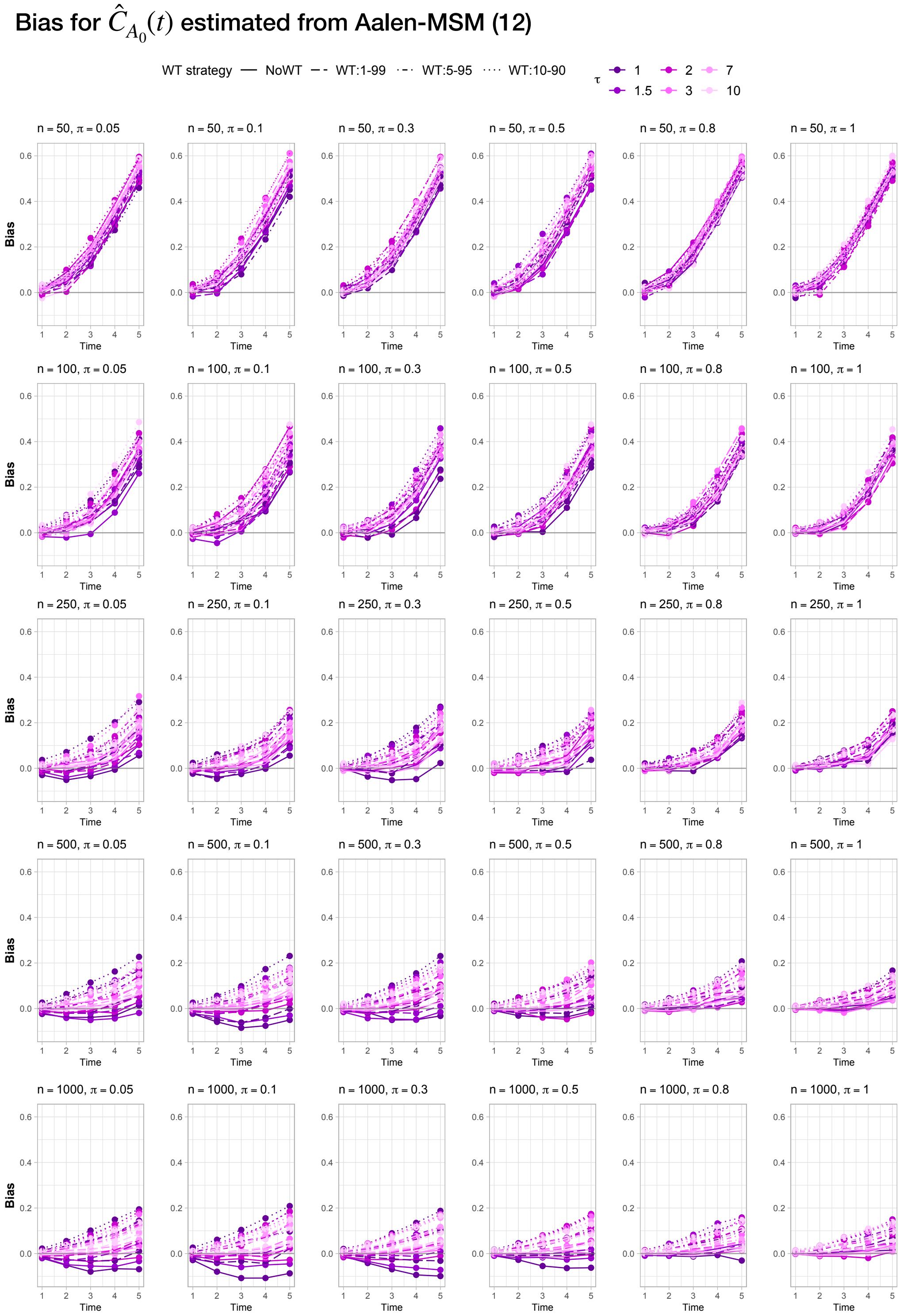

Figure S5: Bias of the estimates for the cumulative coefficient $C_{A0}(t) = \int_0^t \tilde{\alpha}_{A0}(s)ds$ at time points $t = 1, \ldots, 5$ for the different setting of simulation study II. Each row refers to a different sample size $n = 50, 100, 250, 500, 1000$. Each column refers to a different exposure cut-off $\pi = 0.05, 0.1, 0.3, 0.5, 0.8, 1$. Different types of line refer to different weight truncation (WT) strategies (solid: No WT; long-dashed: 1-99 WT; dot-dashed: 5-95 WT; dotted: 10-90 WT). The colours refer to different values of the rule-threshold τ: the darker the colour, the more severe the violation (i.e., the lower τ).

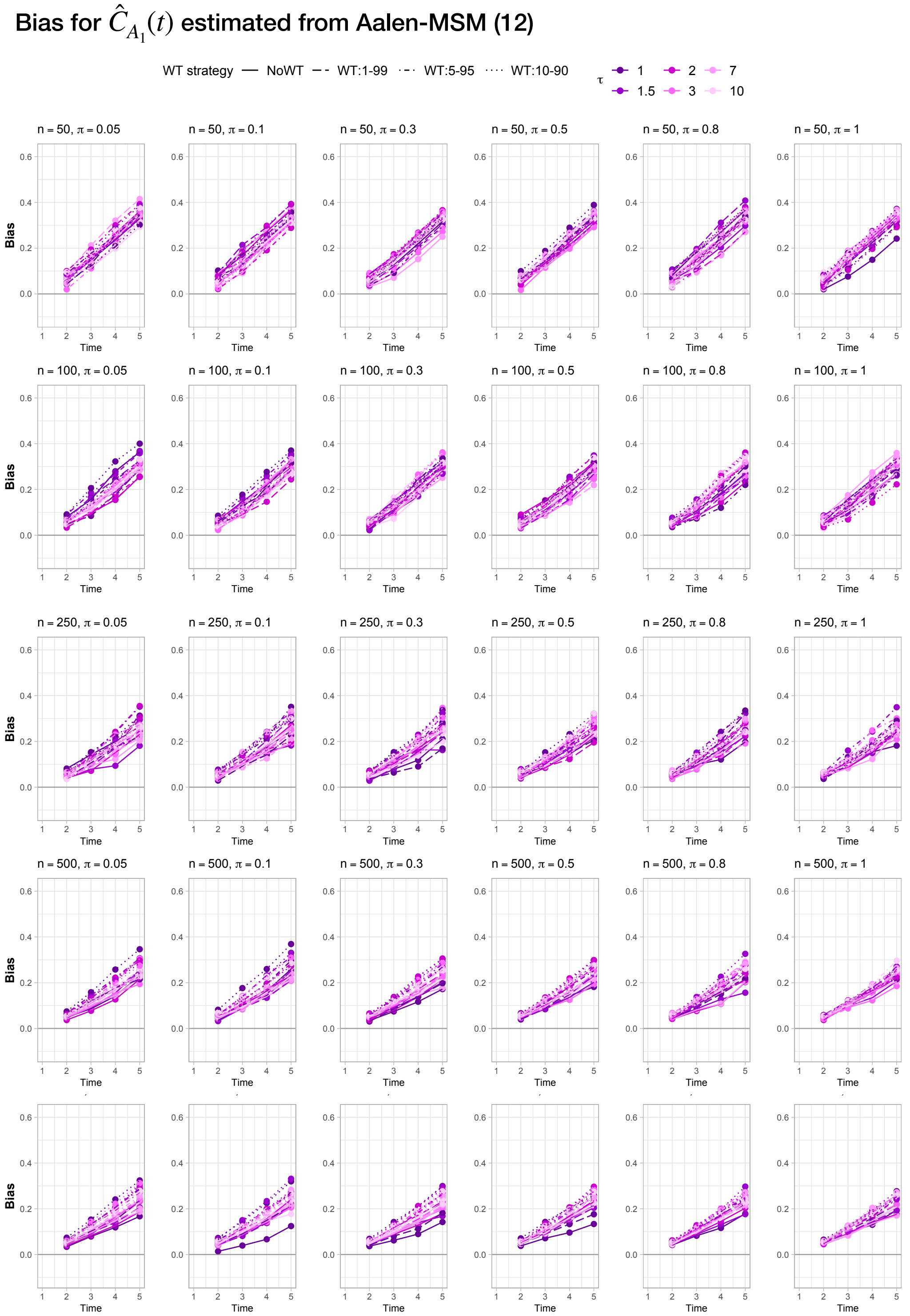

Figure S6: Bias of the estimates for the cumulative coefficient $C_{A1}(t) = \int_1^t \tilde{\alpha}_{A1}(s)ds$ at time points $t = 2, 3, 4, 5$ for the different setting of simulation study II. Each row refers to a different sample size $n = 50, 100, 250, 500, 1000$. Each column refers to a different exposure cut-off $\pi = 0.05, 0.1, 0.3, 0.5, 0.8, 1$. Different types of line refer to different weight truncation (WT) strategies (solid: No WT; long-dashed: 1-99 WT; dot-dashed: 5-95 WT; dotted: 10-90 WT). The colours refer to different values of the rule-threshold τ: the darker the colour, the more severe the violation (i.e., the lower τ).

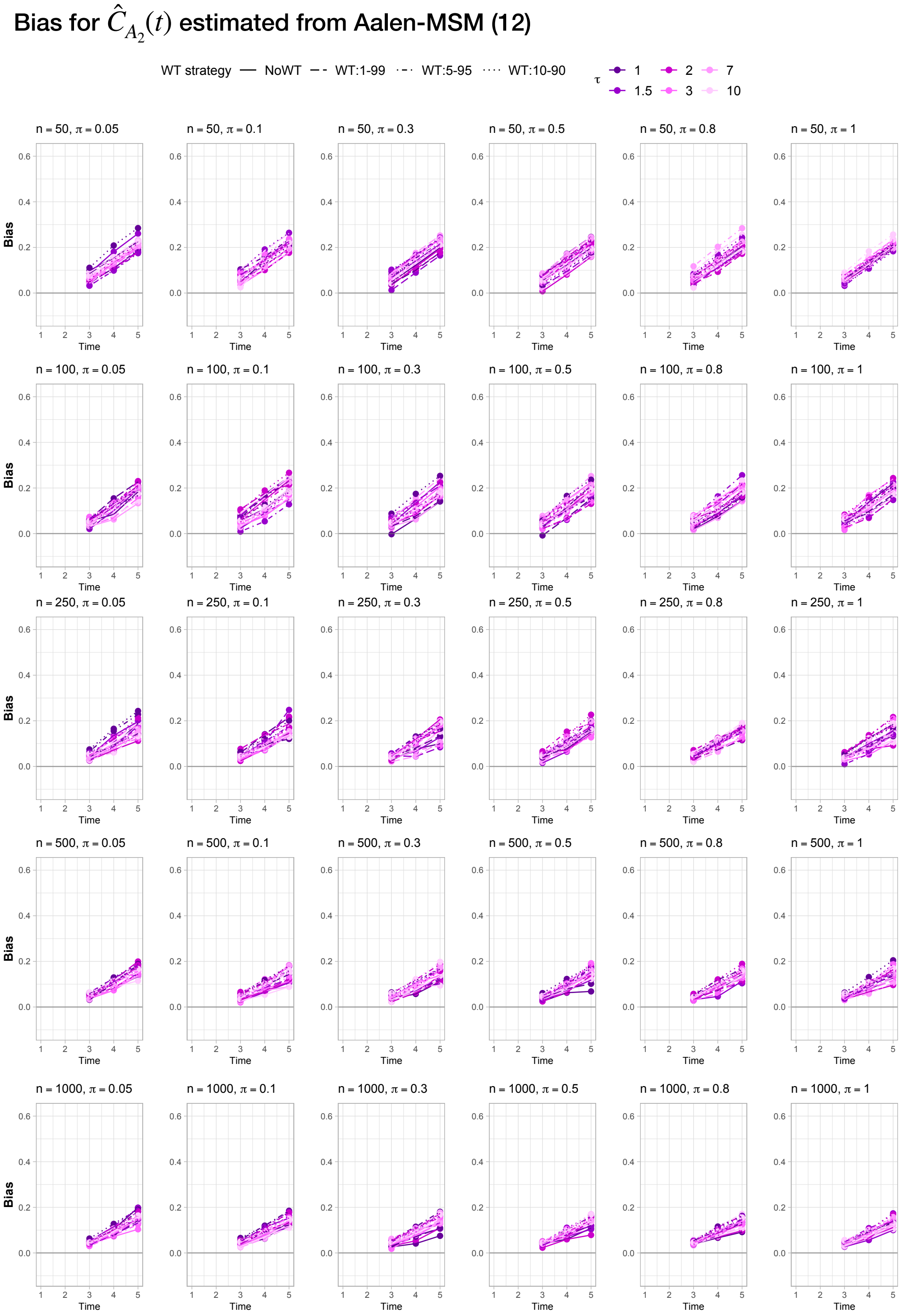

Figure S7: Bias of the estimates for the cumulative coefficient $C_{A2}(t) = \int_2^t \tilde{\alpha}_{A2}(s)ds$ at time points $t = 3, 4, 5$ for the different setting of simulation study II. Each row refers to a different sample size $n = 50, 100, 250, 500, 1000$. Each column refers to a different exposure cut-off $\pi = 0.05, 0.1, 0.3, 0.5, 0.8, 1$. Different types of line refer to different weight truncation (WT) strategies (solid: No WT; long-dashed: 1-99 WT; dot-dashed: 5-95 WT; dotted: 10-90 WT). The colours refer to different values of the rule-threshold τ: the darker the colour, the more severe the violation (i.e., the lower τ).

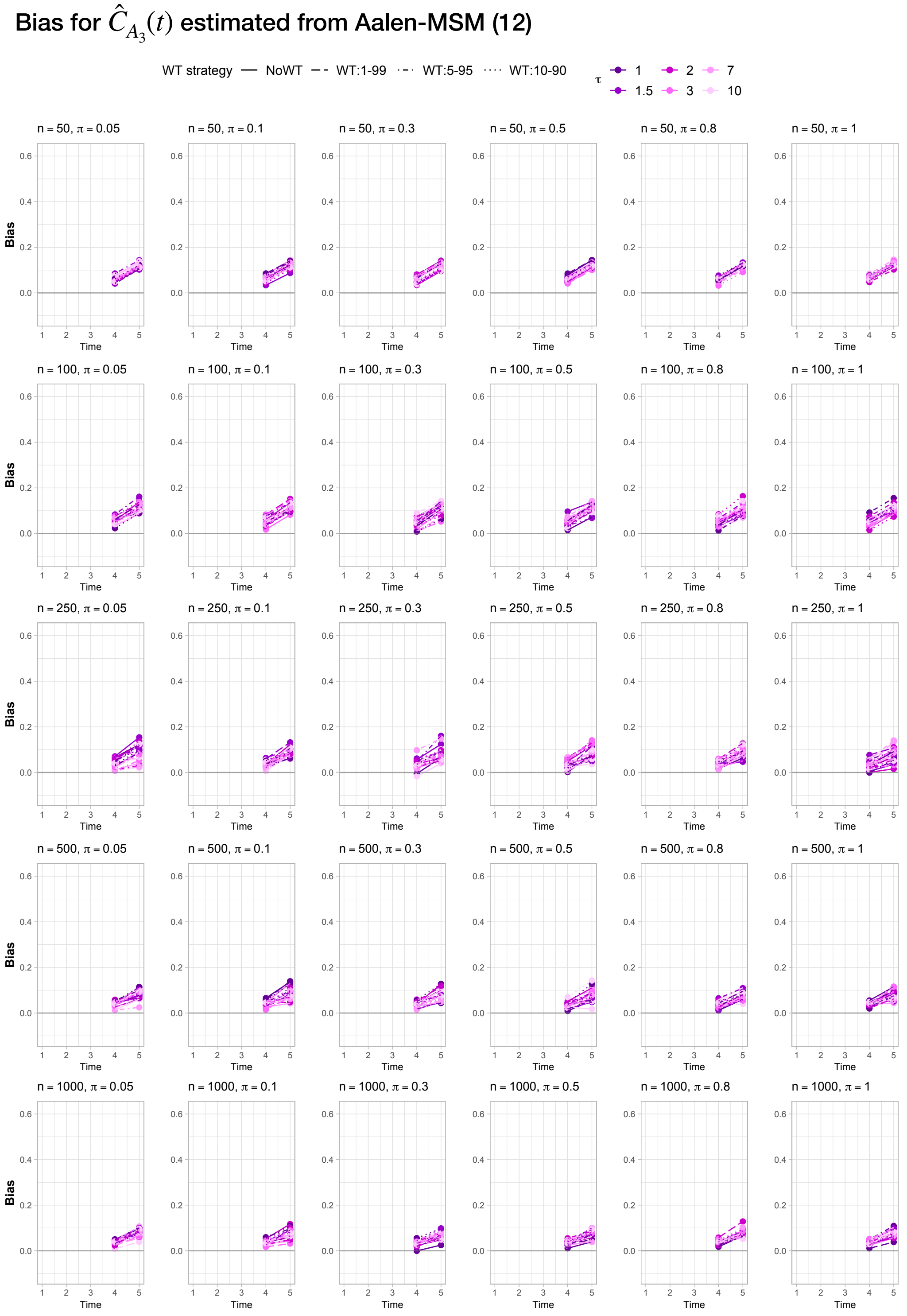

Figure S8: Bias of the estimates for the cumulative coefficient $C_{A3}(t) = \int_3^t \tilde{\alpha}_{A3}(s)ds$ at time points $t = 4, 5$ for the different setting of simulation study II. Each row refers to a different sample size $n = 50, 100, 250, 500, 1000$. Each column refers to a different exposure cut-off $\pi = 0.05, 0.1, 0.3, 0.5, 0.8, 1$. Different types of line refer to different weight truncation (WT) strategies (solid: No WT; long-dashed: 1-99 WT; dot-dashed: 5-95 WT; dotted: 10-90 WT). The colours refer to different values of the rule-threshold τ: the darker the colour, the more severe the violation (i.e., the lower τ).

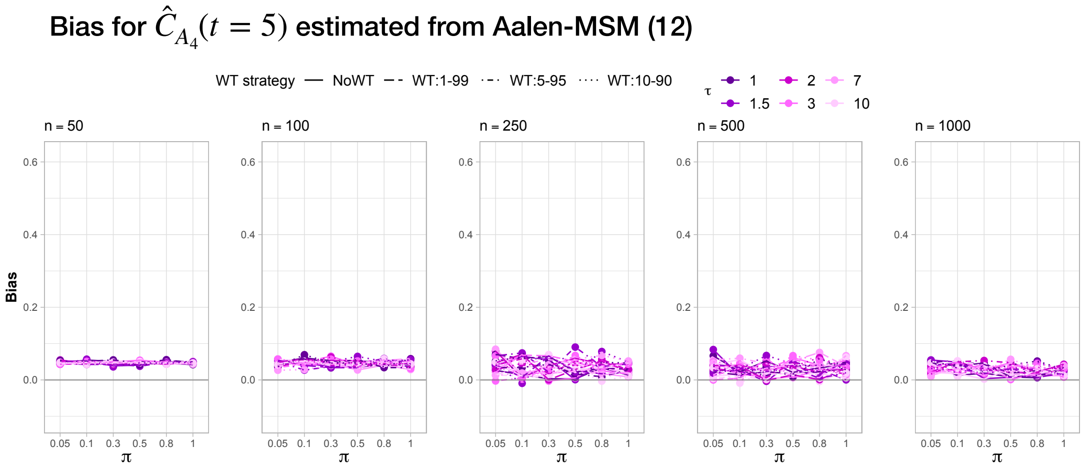

Figure S9: Bias of the estimates for the cumulative coefficient $C_{A4}(t = 5) = \int_4^5 \tilde{\alpha}_{A4}(s)ds$ for the different setting of simulation study II. Each column refers to a different sample size $n = 50, 100, 250, 500, 1000$. The x-axes show the compliance-threshold values $\pi = 0.05, 0.1, 0.3, 0.5, 0.8, 1$. Different types of line refer to different weight truncation (WT) strategies (solid: No WT; long-dashed: 1-99 WT; dot-dashed: 5-95 WT; dotted: 10-90 WT). The colours refer to different values of the rule-threshold τ: the darker the colour, the more severe the violation (i.e., the lower τ).

S2.2 Empirical standard error

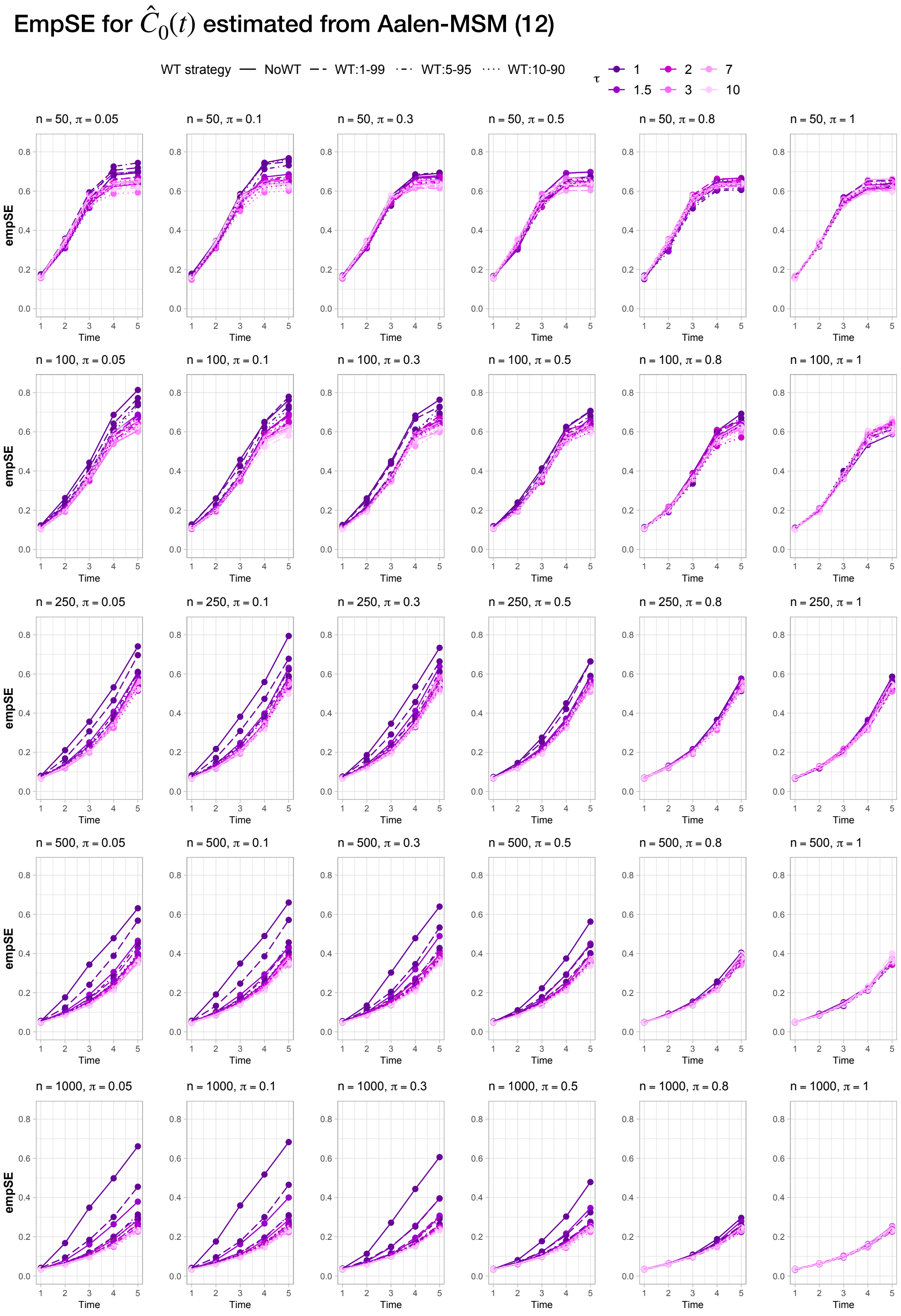

Figure S10: Empirical Standard Error (empSE) of the estimates for the cumulative coefficient $C_0(t) = \int_0^t \tilde{\alpha}_0(s)ds$ at time points $t = 1, \ldots, 5$ for the different setting of simulation study II. Each row refers to a different sample size $n = 50, 100, 250, 500, 1000$. Each column refers to a different exposure cut-off $\pi = 0.05, 0.1, 0.3, 0.5, 0.8, 1$. Different types of line refer to different weight truncation (WT) strategies (solid: No WT; long-dashed: 1-99 WT; dot-dashed: 5-95 WT; dotted: 10-90 WT). The colours refer to different values of the rule-threshold τ: the darker the colour, the more severe the violation (i.e., the lower τ).

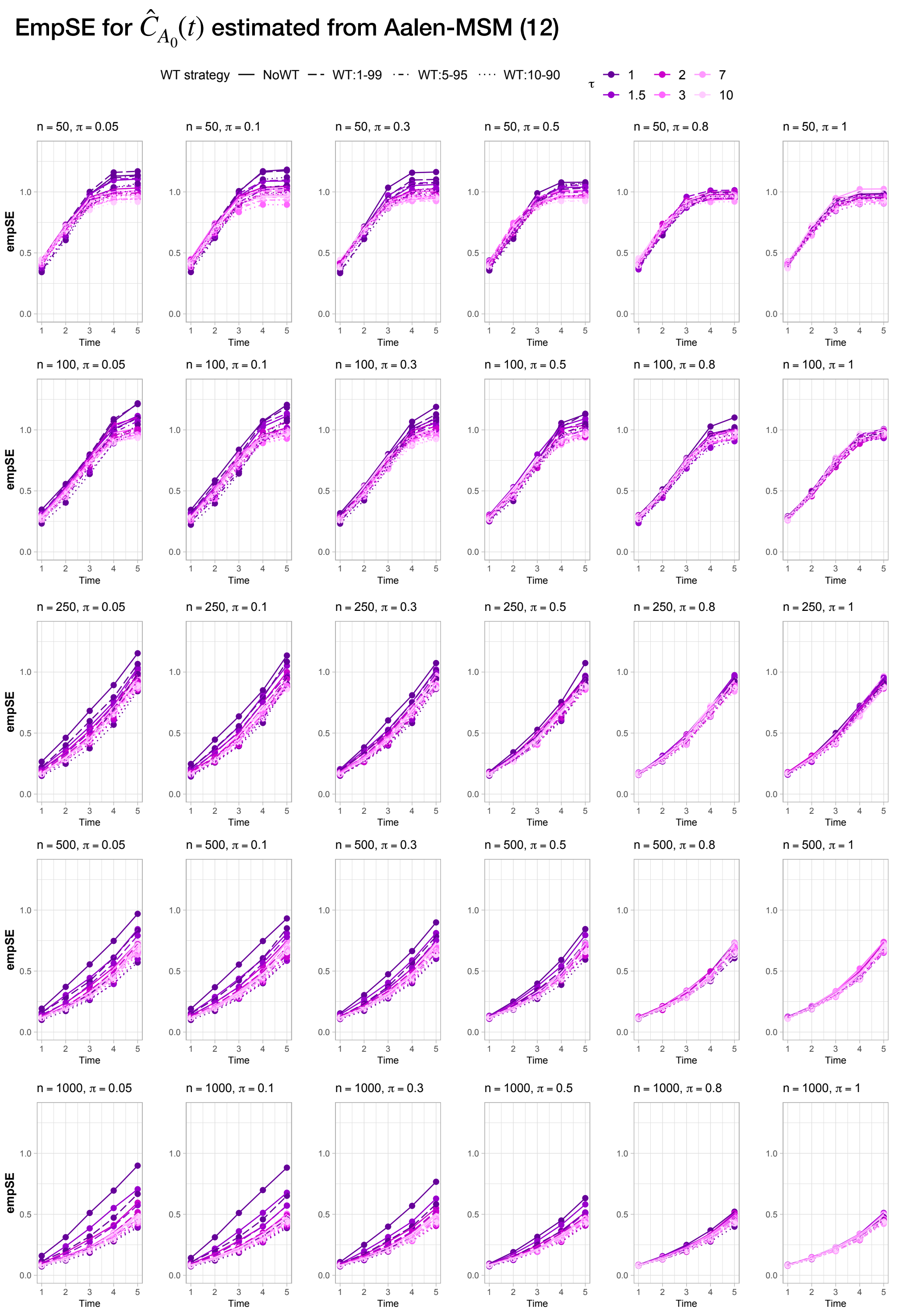

Figure S11: Empirical Standard Error (empSE) of the estimates for the cumulative coefficient $C_{A0}(t) = \int_0^t \tilde{\alpha}_{A0}(s)ds$ at time points $t = 1, \ldots, 5$ for the different setting of simulation study II. Each row refers to a different sample size $n = 50, 100, 250, 500, 1000$. Each column refers to a different exposure cut-off $\pi = 0.05, 0.1, 0.3, 0.5, 0.8, 1$. Different types of line refer to different weight truncation (WT) strategies (solid: No WT; long-dashed: 1-99 WT; dot-dashed: 5-95 WT; dotted: 10-90 WT). The colours refer to different values of the rule-threshold τ: the darker the colour, the more severe the violation (i.e., the lowerτ).

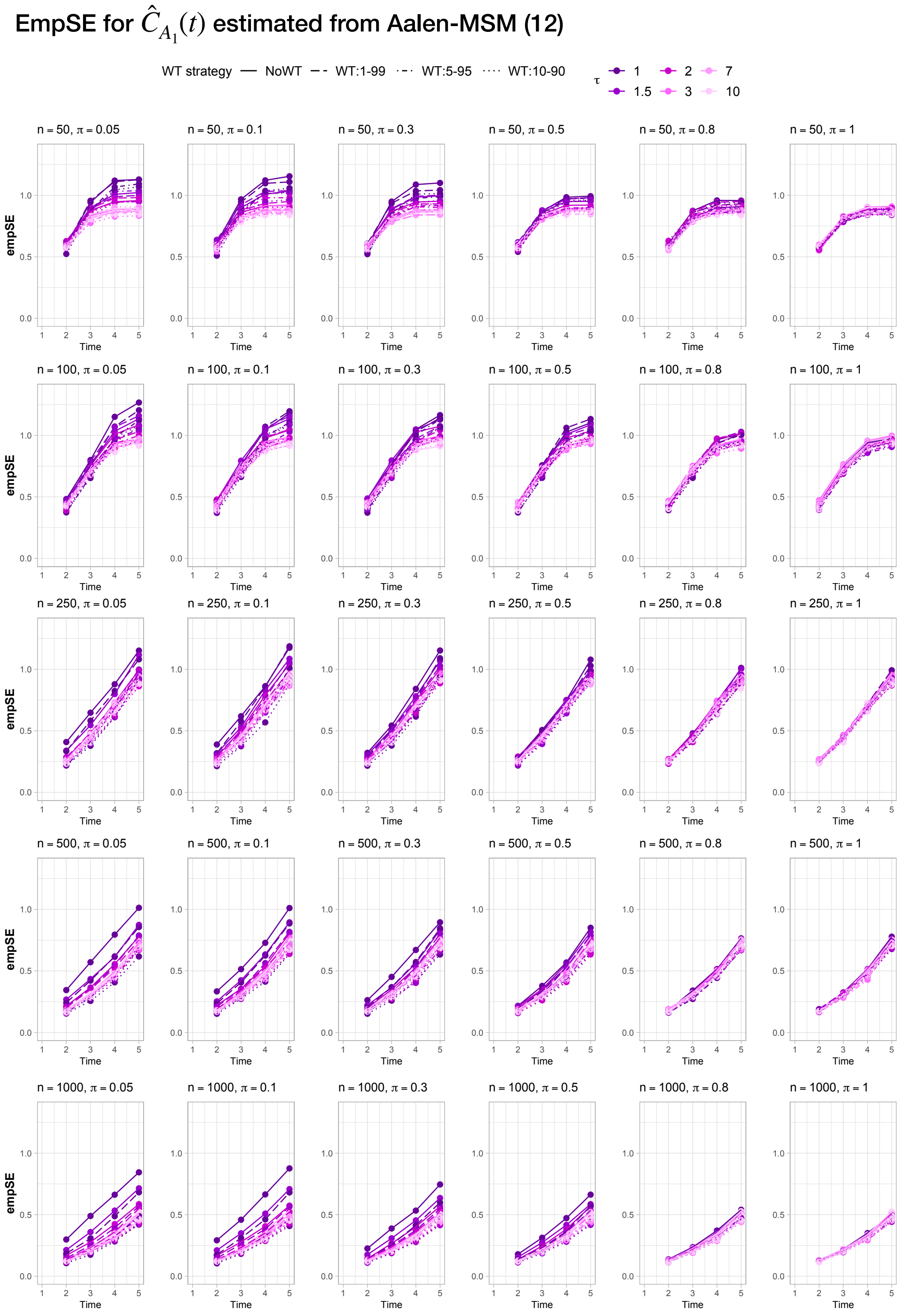

Figure S12: Empirical Standard Error (empSE)of the estimates for the cumulative coefficient $C_{A1}(t) = \int_1^t \tilde{\alpha}_{A1}(s)ds$ at time points $t = 2, 3, 4, 5$ for the different setting of simulation study II. Each row refers to a different sample size $n = 50, 100, 250, 500, 1000$. Each column refers to a different exposure cut-off $\pi = 0.05, 0.1, 0.3, 0.5, 0.8, 1$. Different types of line refer to different weight truncation (WT) strategies (solid: No WT; long-dashed: 1-99 WT; dot-dashed: 5-95 WT; dotted: 10-90 WT). The colours refer to different values of the rule-threshold τ: the darker the colour, the more severe the violation (i.e., the lower τ).

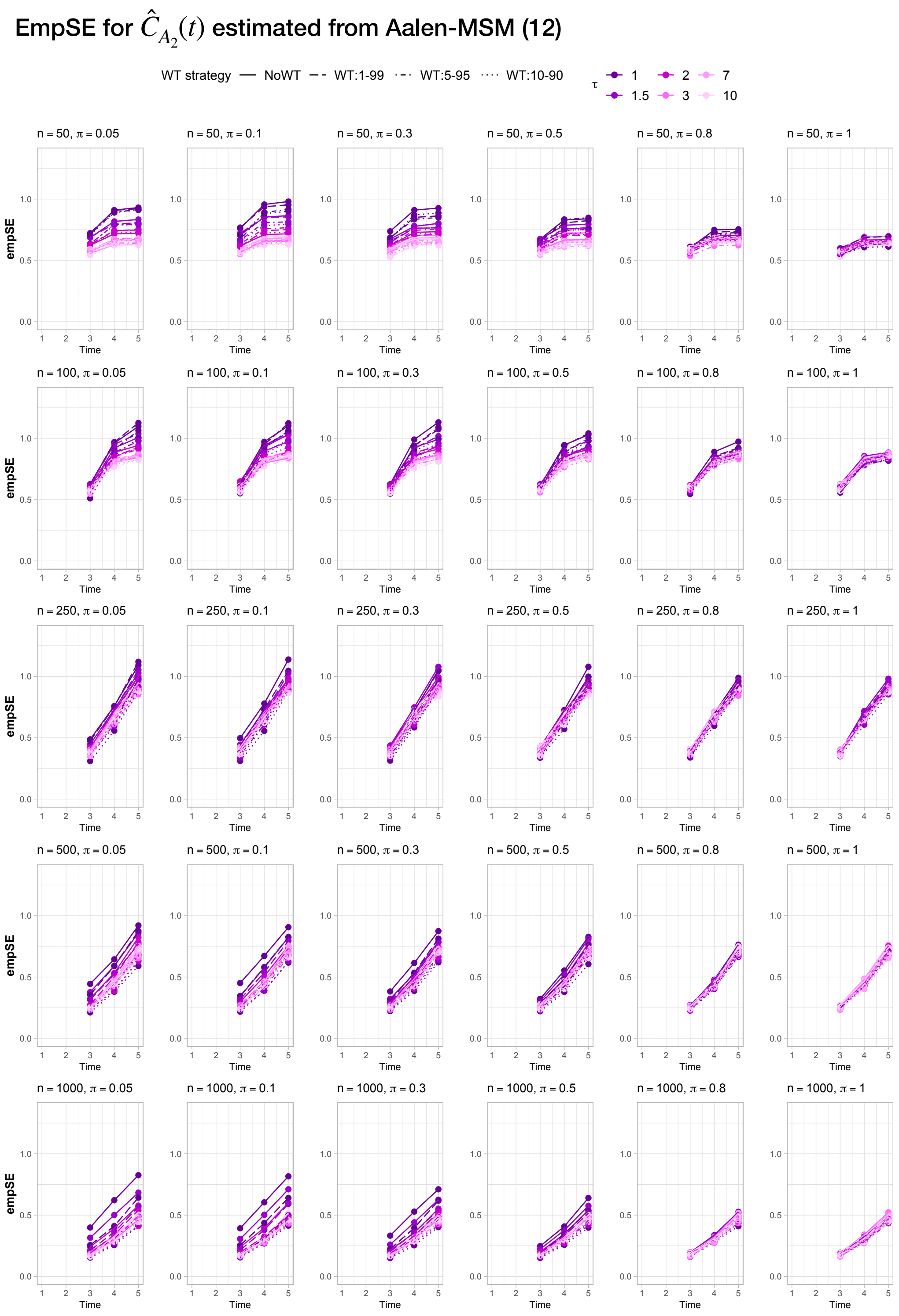

Figure S13: Empirical Standard Error (empSE)of the estimates for the cumulative coefficient $C_{A2}(t) = \int_2^t \tilde{\alpha}_{A2}(s)ds$ at time points $t = 3, 4, 5$ for the different setting of simulation study II. Each row refers to a different sample size $n = 50, 100, 250, 500, 1000$. Each column refers to a different exposure cut-off $\pi = 0.05, 0.1, 0.3, 0.5, 0.8, 1$. Different types of line refer to different weight truncation (WT) strategies (solid: No WT; long-dashed: 1-99 WT; dot-dashed: 5-95 WT; dotted: 10-90 WT). The colours refer to different values of the rule-threshold τ: the darker the colour, the more severe the violation (i.e., the lower τ).

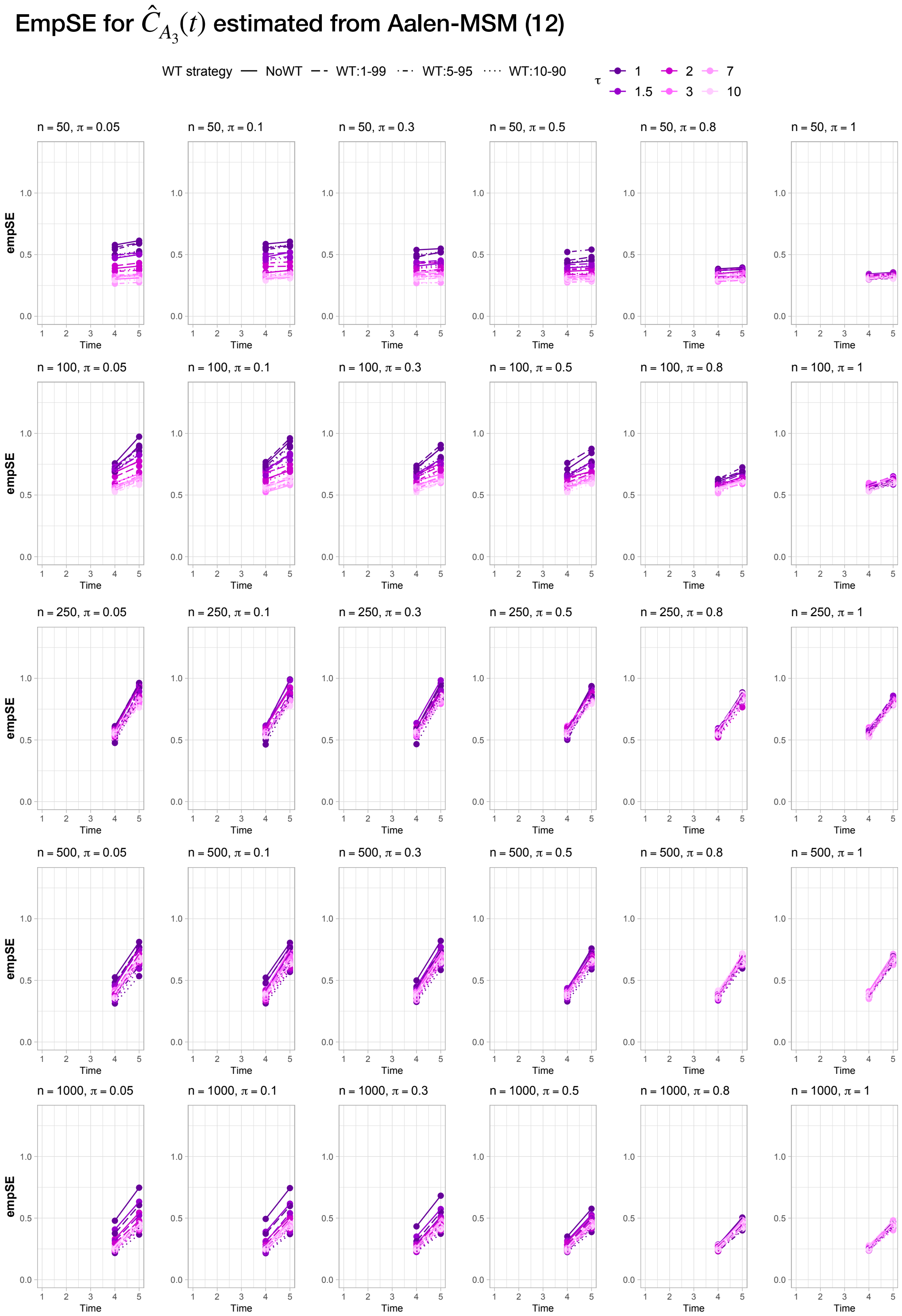

Figure S14: Empirical Standard Error (empSE)of the estimates for the cumulative coefficient $C_{A3}(t) = \int_3^t \tilde{\alpha}_{A3}(s)ds$ at time points $t = 4, 5$ for the different setting of simulation study II. Each row refers to a different sample size $n = 50, 100, 250, 500, 1000$. Each column refers to a different exposure cut-off $\pi = 0.05, 0.1, 0.3, 0.5, 0.8, 1$. Different types of line refer to different weight truncation (WT) strategies (solid: No WT; long-dashed: 1-99 WT; dot-dashed: 5-95 WT; dotted: 10-90 WT). The colours refer to different values of the rule-threshold τ: the darker the colour, the more severe the violation (i.e., the lower τ).

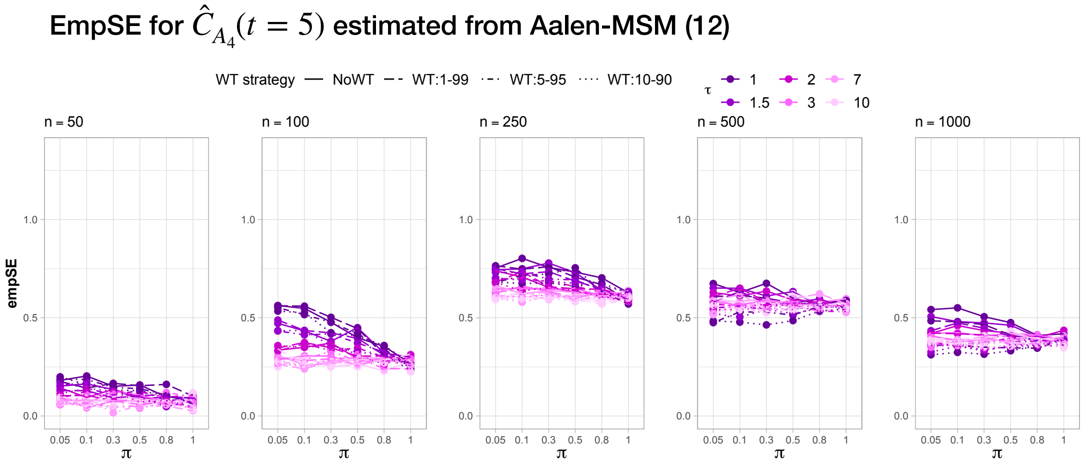

Figure S15: Empirical Standard Error (empSE) of the estimates for the cumulative coefficient $C_{A4}(t = 5) = \int_4^5 \tilde{\alpha}_{A4}(s)ds$ for the different setting of simulation study II. Each column refers to a different sample size $n = 50, 100, 250, 500, 1000$. The x-axes show the compliance-threshold values $\pi = 0.05, 0.1, 0.3, 0.5, 0.8, 1$. Different types of line refer to different weight truncation (WT) strategies (solid: No WT; long-dashed: 1-99 WT; dot-dashed: 5-95 WT; dotted: 10-90 WT). The colours refer to different values of the rule-threshold τ: the darker the colour, the more severe the violation (i.e., the lower τ).

S2.3 Root mean squared error

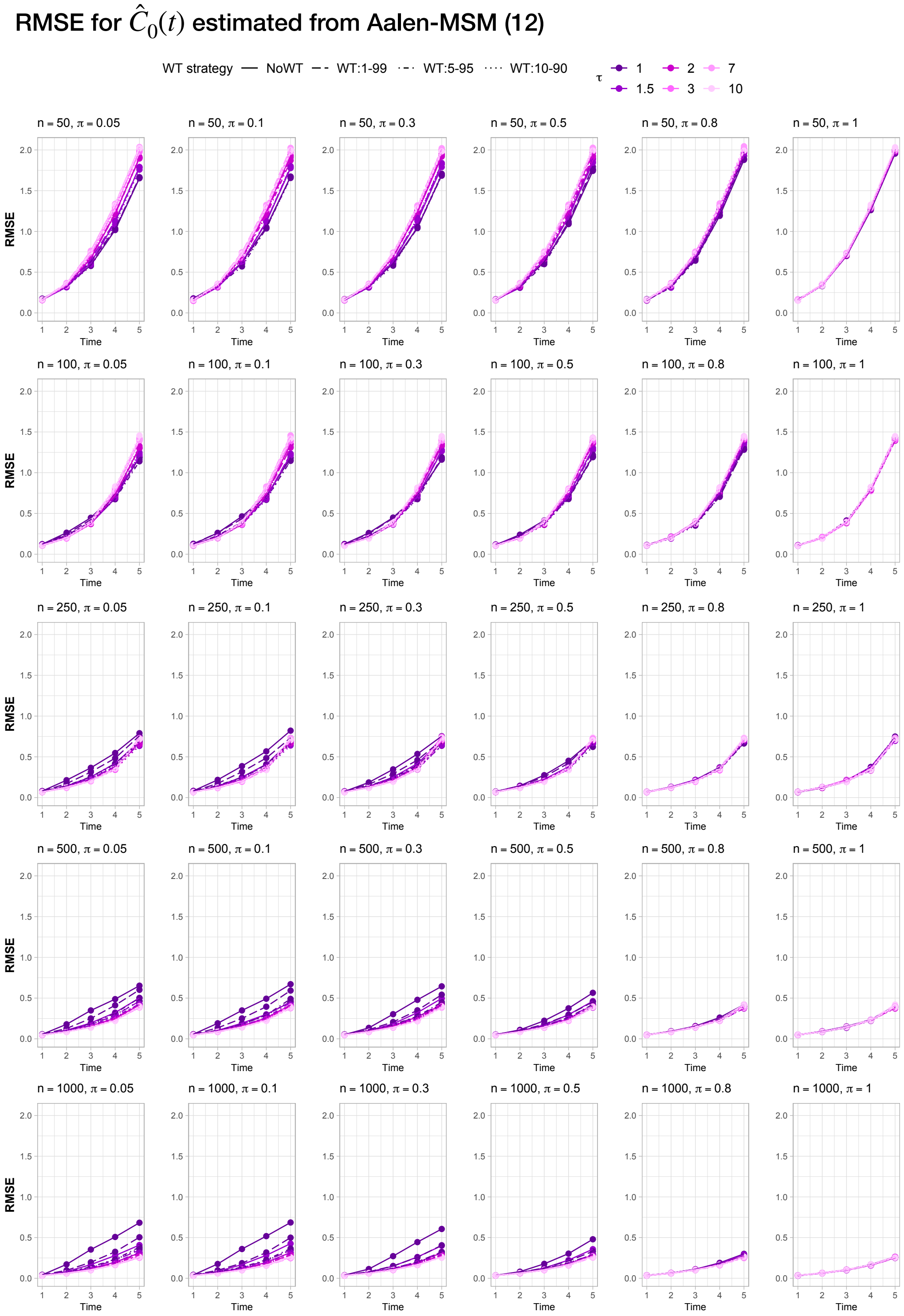

Figure S16: Root Mean Squared Error (RMSE) of the estimates for the cumulative coefficient $C_0(t) = \int_0^t \tilde{\alpha}_0(s)ds$ at time points $t = 1, \ldots, 5$ for the different setting of simulation study II. Each row refers to a different sample size $n = 50, 100, 250, 500, 1000$. Each column refers to a different exposure cut-off $\pi = 0.05, 0.1, 0.3, 0.5, 0.8, 1$. Different types of line refer to different weight truncation (WT) strategies (solid: No WT; long-dashed: 1-99 WT; dot-dashed: 5-95 WT; dotted: 10-90 WT). The colours refer to different values of the rule-threshold τ: the darker the colour, the more severe the violation (i.e., the lower τ).

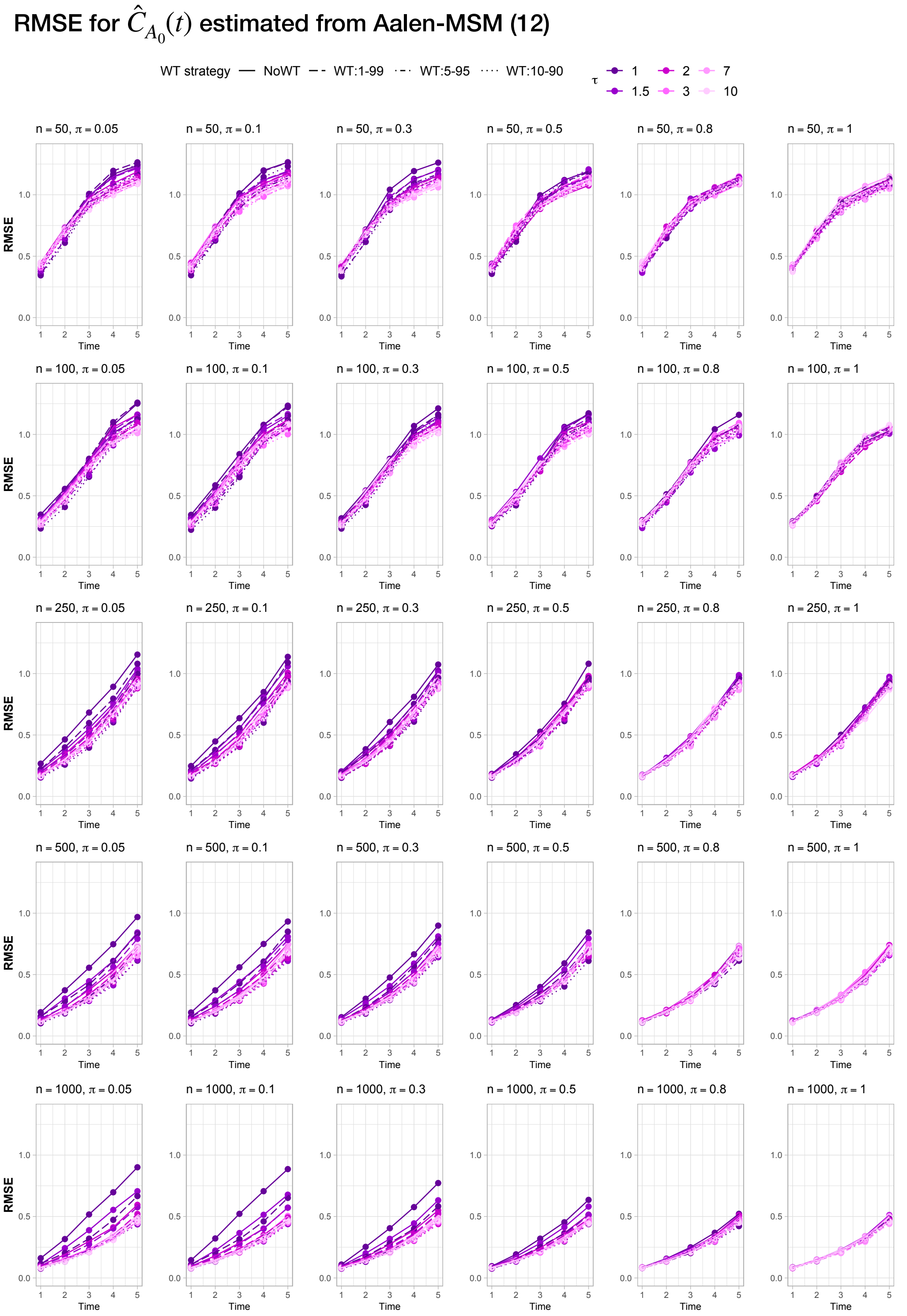

Figure S17: Root Mean Squared Error (RMSE) of the estimates for the cumulative coefficient $C_{A0}(t) = \int_0^t \tilde{\alpha}_{A0}(s)ds$ at time points $t = 1, \ldots, 5$ for the different setting of simulation study II. Each row refers to a different sample size $n = 50, 100, 250, 500, 1000$. Each column refers to a different exposure cut-off $\pi = 0.05, 0.1, 0.3, 0.5, 0.8, 1$. Different types of line refer to different weight truncation (WT) strategies (solid: No WT; long-dashed: 1-99 WT; dot-dashed: 5-95 WT; dotted: 10-90 WT). The colours refer to different values of the rule-threshold τ: the darker the colour, the more severe the violation (i.e., the lower τ).

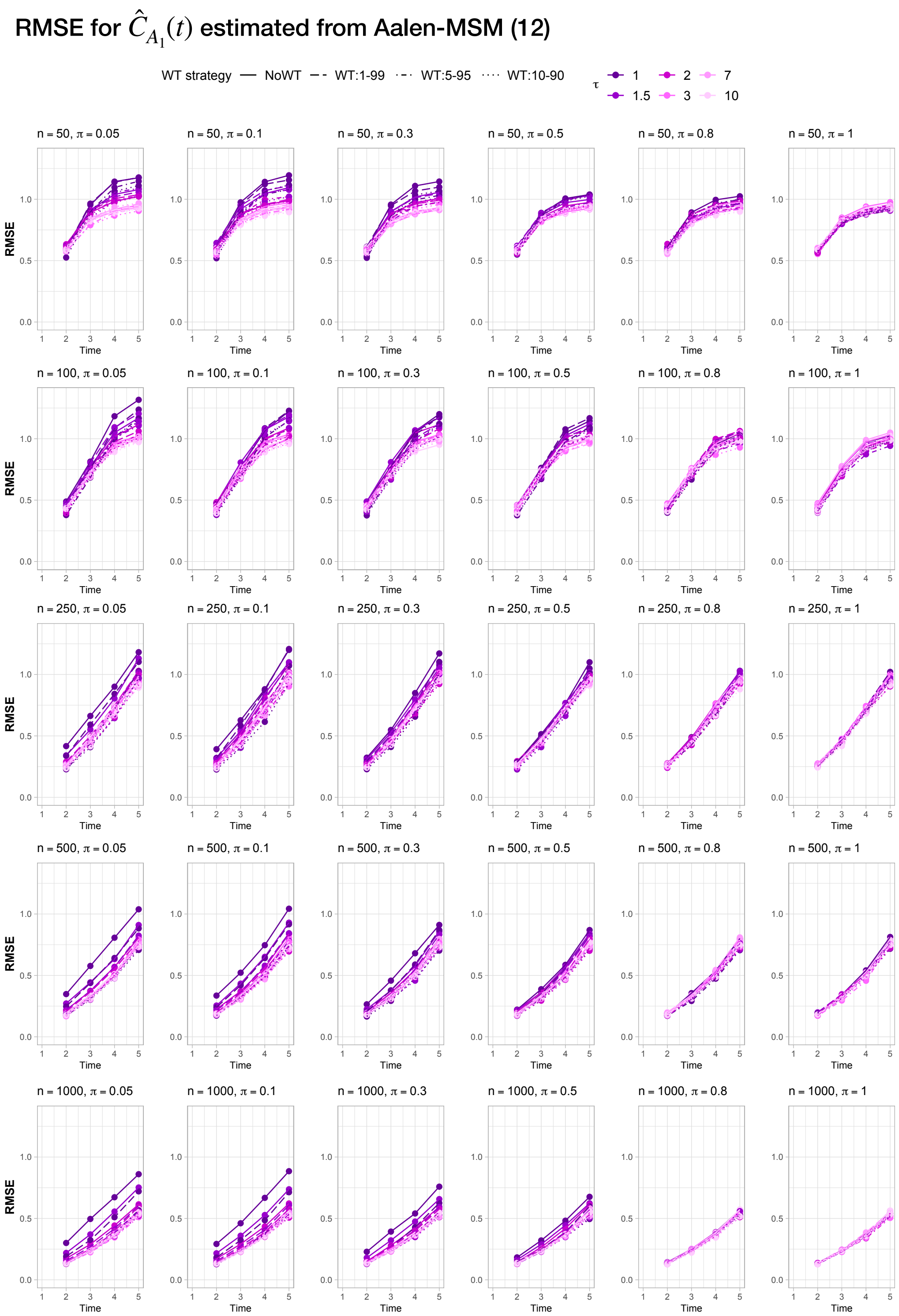

Figure S18: Root Mean Squared Error (RMSE) of the estimates for the cumulative coefficient $C_{A1}(t) = \int_1^t \tilde{\alpha}_{A1}(s)ds$ at time points $t = 2, 3, 4, 5$ for the different setting of simulation study II. Each row refers to a different sample size $n = 50, 100, 250, 500, 1000$. Each column refers to a different exposure cut-off $\pi = 0.05, 0.1, 0.3, 0.5, 0.8, 1$. Different types of line refer to different weight truncation (WT) strategies (solid: No WT; long-dashed: 1-99 WT; dot-dashed: 5-95 WT; dotted: 10-90 WT). The colours refer to different values of the rule-threshold τ: the darker the colour, the more severe the violation (i.e., the lower τ).

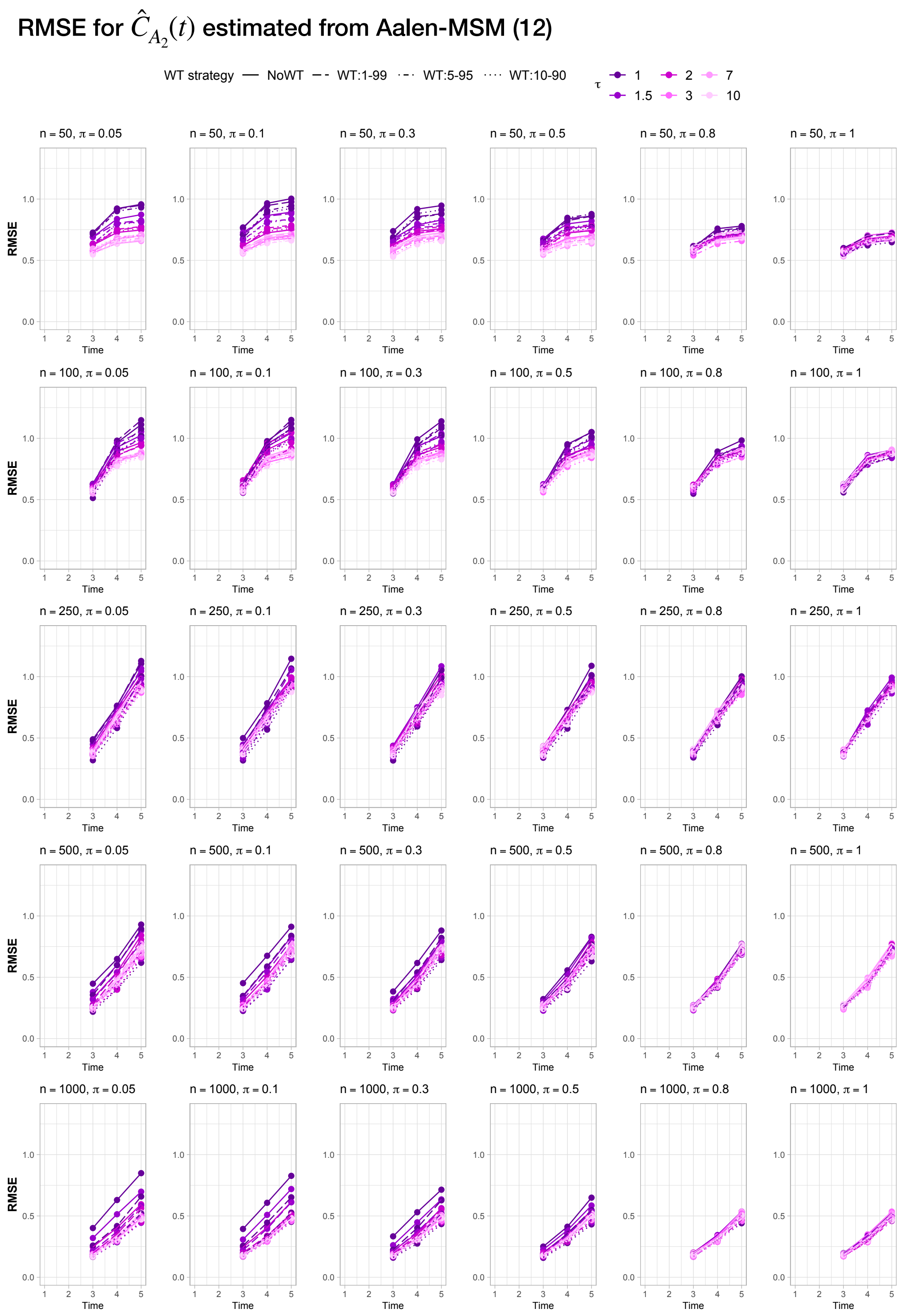

Figure S19: Root Mean Squared Error (RMSE)of the estimates for the cumulative coefficient $C_{A2}(t) = \int_2^t \tilde{\alpha}_{A2}(s)ds$ at time points $t = 3, 4, 5$ for the different setting of simulation study II. Each row refers to a different sample size $n = 50, 100, 250, 500, 1000$. Each column refers to a different exposure cut-off $\pi = 0.05, 0.1, 0.3, 0.5, 0.8, 1$. Different types of line refer to different weight truncation (WT) strategies (solid: No WT; long-dashed: 1-99 WT; dot-dashed: 5-95 WT; dotted: 10-90 WT). The colours refer to different values of the rule-threshold τ: the darker the colour, the more severe the violation (i.e., the lower τ).

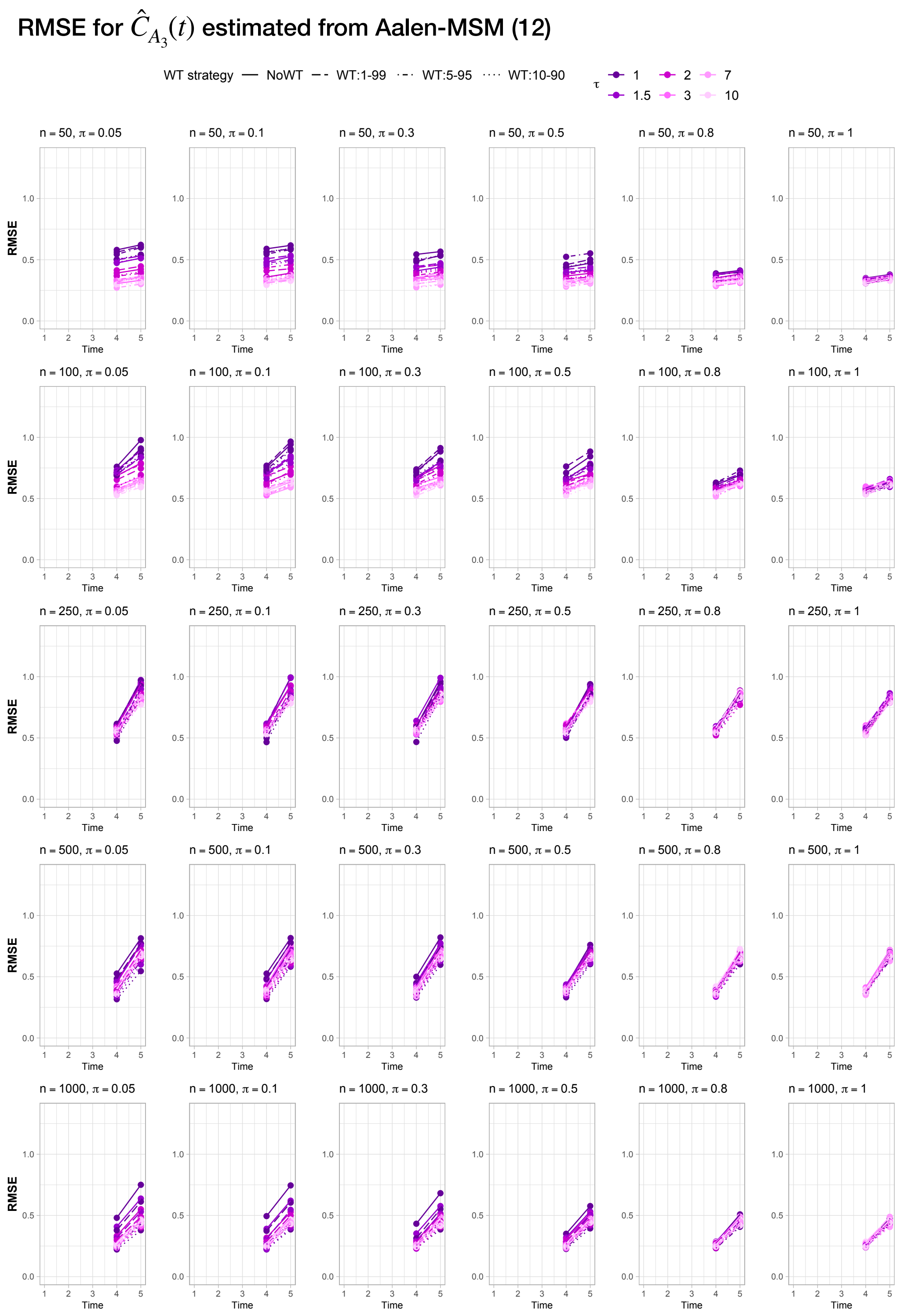

Figure S20: Root Mean Squared Error (RMSE)of the estimates for the cumulative coefficient $C_{A3}(t) = \int_3^t \tilde{\alpha}_{A3}(s)ds$ at time points $t = 4, 5$ for the different setting of simulation study II. Each row refers to a different sample size $n = 50, 100, 250, 500, 1000$. Each column refers to a different exposure cut-off $\pi = 0.05, 0.1, 0.3, 0.5, 0.8, 1$. Different types of line refer to different weight truncation (WT) strategies (solid: No WT; long-dashed: 1-99 WT; dot-dashed: 5-95 WT; dotted: 10-90 WT). The colours refer to different values of the rule-threshold τ: the darker the colour, the more severe the violation (i.e., the lower τ).

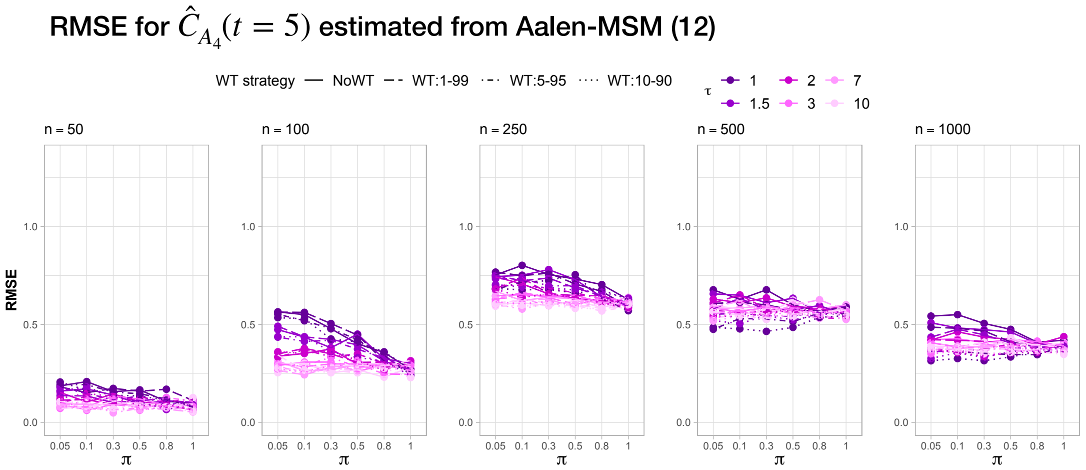

Figure S21: Root Mean Squared Error (RMSE) of the estimates for the cumulative coefficient $C_{A4}(t = 5) = \int_4^5 \tilde{\alpha}_{A4}(s)ds$ for the different setting of simulation study II. Each column refers to a different sample size $n = 50, 100, 250, 500, 1000$. The x-axes show the compliance-threshold values $\pi = 0.05, 0.1, 0.3, 0.5, 0.8, 1$. Different types of line refer to different weight truncation (WT) strategies (solid: No WT; long-dashed: 1-99 WT; dot-dashed: 5-95 WT; dotted: 10-90 WT). The colours refer to different values of the rule-threshold τ: the darker the colour, the more severe the violation (i.e., the lower τ).
