# Supplementary material for: Impact of Near‐Positivity Violations on IPTW‐Estimated Marginal Structural Survival Models With Time‐Dependent Confounding
Source: Biom J. 2025 Nov 3;67(6):e70093. doi: 10.1002/bimj.70093 (PMC12581517; doi:10.1002/bimj.70093)
Supplement: Supplementary file 3 — Supporting File 3: bimj70093‐sup‐0003‐SuppMat.pdf. [file BIMJ-67-e70093-s002.pdf]

# Supplementary Vignette

## Impact of near-positivity violations on IPTW-estimated marginal structural survival models with time-dependent confounding

Marta Spreafico

27-06-2025

This supplementary material aims to serve as a tutorial vignette, illustrating how to conduct the analysis using the code for both Algorithm I and II in two distinct contexts: first, using a single dataset emulating the context a real-data application, and second, employing $B$ simulated datasets to demonstrate a simulation-style approach within a single $(n, WT, \pi, \tau)$ scenario.

Download the R code from the online Supporting Information to the article or from https://github.com/mspreafico/PosViolMSM. All the functions required for this vignette are available in the files contained in the *functions* folder.

# I. Analysis using Algorithm I

## I.a Example of a single simulated dataset

**Step 1 | Simulate data.** Algorithm I (see Manuscript Section 4.1.2) is implemented in the function `sim.algorithmI`, available in the file *functions/algorithm_I.R*.

Using this function, let us simulate a dataset of $n = 300$ subjects (`n`) over $K = 40$ time-visits (`K`) with check-ups every ($\kappa = 5$)-th visit (`kappa`), considering an exposure cut-off of $\pi = 0.15$ (`pi.prop`) and a poor-health subgroup $\mathcal{I}_\tau = [0, \tau)$ with threshold $\tau = 400$ (`tau`).

*Remark.* As mentioned in Manuscript Section 4.2.1, by default the data are simulated in such a way that the true values of the parameters in desired marginal structural logistic regression model (logit-MSM) are $(\tilde{\gamma}_0^*, \tilde{\gamma}_{A1}^*, \tilde{\gamma}_{A2}^*, \tilde{\gamma}_{A3}^*) = (-3, 0.05, -1.5, 0.1)$.

```
source("functions/algorithm_I.R")
K.visits = 40
k.checkup = 5

set.seed(5678)
df = sim.algorithmI(pi.prop = 0.15, tau = 400, n = 300,
                    K = K.visits, kappa = k.checkup)
df[1:8,]
```

```
##    id visit Y A A_1        L d1 d3
## 1:  1     0 0 1   0 375.1453  0  0
## 2:  1     1 0 1   1 375.1453  0  1
## 3:  1     2 0 1   1 375.1453  0  2
## 4:  1     3 0 1   1 375.1453  0  3
## 5:  1     4 0 1   1 375.1453  0  4
## 6:  1     5 0 1   1 398.0477  0  5
## 7:  1     6 0 1   1 398.0477  0  6
## 8:  1     7 0 1   1 398.0477  0  7
```

The simulated dataset contains:

- `id`: subject index $i$
- `visit`: visit index $k$
- `Y`: observed survival outcome $Y_{i,k+1}$
- `A`: observed HAART exposure $A_{i,k}$
- `A_1`: observed HAART exposure at the previous visit $A_{i,k-1}$, with $A_{i,0} = 0$
- `L`: observed CD4 cell count $L_{i,k}$
- `d1`: time elapsed before after treatment initiation $d_{1k} = \min\{k, k^*\}$
- `d3`: time elapsed after treatment initiation $d_{3k} = \max\{k - k^*, 0\}$

**Step 2 | Compute IPTW weights.** As mentioned in Manuscript Section 4.2.1, weight components at each check-up visit $(k = 0, \kappa, 2\kappa, \ldots)$ are estimated by logistic regression models for the probability of treatment initiation, with numerator

$$\Pr\left(A_{i,k} = 1 \mid \bar{\boldsymbol{A}}_{i,k-1} = \bar{\mathbf{0}}, T_i \geq k\right) = logit^{-1}\left[\theta_0 + \theta_1 \cdot A_{i,k-1}\right]$$

and denominator

$$\Pr\left(A_{i,k} = 1 \mid \bar{\boldsymbol{A}}_{i,k-1} = \bar{\mathbf{0}}, \bar{\boldsymbol{L}}_{i,k}, T_i \geq k\right) = logit^{-1}\left[\theta_0 + \theta_1 \cdot A_{i,k-1} + \theta_2 \cdot L_{i,k}\right].$$

The stabilized weight for subject $i$ at time $t$ is then defined as:

$$sw_i(t) = \prod_{k=0}^{\lfloor t \rfloor} \frac{\Pr\left(A_{i,k} = 1 \mid \bar{\boldsymbol{A}}_{i,k-1} = \bar{\mathbf{0}}, T_i \geq k\right)}{\Pr\left(A_{i,k} = 1 \mid \bar{\boldsymbol{A}}_{i,k-1} = \bar{\mathbf{0}}, \bar{\boldsymbol{L}}_{i,k}, T_i \geq k\right)}$$

where $\lfloor t \rfloor$ is the largest check-up-visit-time prior to $t$.

The function `get.std.weightsI` from file *functions/iptw_I.R* computes the IPTW standardized weights $sw_i(t)$. By default, stabilized weights are computed without weight truncation (WT).

```
source("functions/iptw_I.R")

# No Weight Truncation (NoWT)
df_sw = get.std.weightsI(df, trunc = FALSE)
summary(df_sw$sw)
```

```
##      Min.   1st Qu.    Median      Mean   3rd Qu.      Max.
##   0.00948   0.40018   0.68786   1.55880   0.80552 153.27824
```

To apply WT to 1st and 9th percentiles (WT 1-99), parameters `trunc` must be `TRUE` and the desired lower and upper percentiles must be specified in parameter `percentiles`, as follows:

```
# Weight Truncation 1st-99th percentiles (WT 1-99)
df_sw_wt = get.std.weightsI(df, trunc = TRUE, percentiles = c(1,99))
summary(df_sw_wt$sw)
```

```
##    Min. 1st Qu.  Median    Mean 3rd Qu.    Max.
## 0.03273 0.40018 0.68785 0.85159 0.80553 9.13815
```

The logarithm of estimated standardized weights $\log\left(\widehat{sw}_i(t)\right)$ can be used to check the distributions of the weights over check-ups times:

```
check_ups = seq(0, K.visits, by=k.checkup)
par(mfrow = c(1, 2))
boxplot(log(sw) ~ visit, data = df_sw[df_sw$visit %in% check_ups,],
        xlab = 'Time-visit k', ylab = 'Logarithm of standardized weights',
        main = 'No Weight Truncation', ylim=c(-5,5))
boxplot(log(sw) ~ visit, data = df_sw_wt[df_sw$visit %in% check_ups,],
        xlab = 'Time-visit k', ylab = 'Logarithm of standardized weights',
        main = 'Weight Truncation 1-99', ylim=c(-5,5))
```

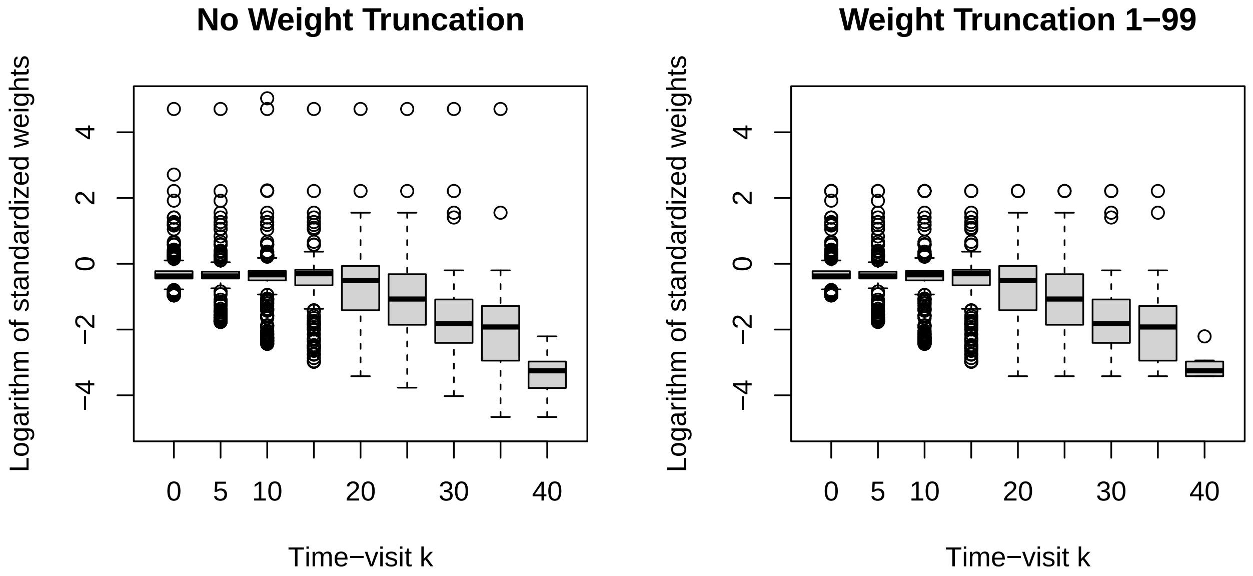


**Step 3 | Estimate logit-MSM.** The desired logit-MSM under treatment $\bar{\boldsymbol{a}}$ at time $k$ (see Manuscript Section 4.1.1) is

$$\lambda_k^{\bar{\boldsymbol{a}}} = \text{logit}^{-1}\left[\tilde{\gamma}_0 + \tilde{\gamma}_{A1} \cdot d_{1k} + \tilde{\gamma}_{A2} \cdot a_k + \tilde{\gamma}_{A3} \cdot d_{3k}\right].$$

It can be estimated from the data by applying IPTW to the conditional logistic regression model:

$$\lambda_{i,k} = \text{logit}^{-1}\left[\gamma_0 + \gamma_{A1} \cdot D_{1\,i,k} + \gamma_{A2} \cdot A_{i,k} + \gamma_{A3} \cdot D_{3\,i,k}\right].$$

This can be done by employing the `glm` function with `weights=sw`. The estimated coefficients $(\hat{\gamma}_0, \hat{\gamma}_{A1}, \hat{\gamma}_{A2}, \hat{\gamma}_{A3})$ without WT are

```
est_coefs = coef(glm(Y ~ d1 + A + d3, family=quasibinomial, data=df_sw, weights=sw))
est_coefs
```

```
## (Intercept)          d1           A          d3
## -2.43349354  0.13547876 -2.71406976  0.09636121
```

and under WT 1-99:

```
est_coefs_wt = coef(glm(Y ~ d1 + A + d3, family=quasibinomial, data=df_sw_wt, weights=sw))
est_coefs_wt
```

```
## (Intercept)          d1           A          d3
## -2.97866032  0.03448066 -1.27477735  0.09372310
```

while the true values are $(\gamma_0, \gamma_{A1}, \gamma_{A2}, \gamma_{A3}) = (-3, 0.05, -1.5, 0.1)$.

**Step 4 | Marginal Survival Curves.** For logit-MSMs, the marginal survival probability at time $t$ under treatment history $\bar{\boldsymbol{a}}$ is defined as $S^{\bar{\boldsymbol{a}}}(t) = \Pr(T^{\bar{\boldsymbol{a}}} > t) = \prod_{k \le t}(1 - \lambda_k^{\bar{\boldsymbol{a}}})$.

By substituting $\lambda_k^{\bar{\boldsymbol{a}}}$ with the desired logit-MSM, the marginal survival probabilities for the *never treated* ($\bar{\boldsymbol{a}} = \bar{\mathbf{0}}$) and *always treated* ($\bar{\boldsymbol{a}} = \bar{\mathbf{1}}$) groups are:

$$S^{\bar{\mathbf{0}}}(t) = \prod_{k \leq t} \left(1 - \text{logit}^{-1}\left[\tilde{\gamma}_0 + \tilde{\gamma}_{A1} \cdot k\right]\right),$$

$$S^{\bar{\mathbf{1}}}(t) = \prod_{k \leq t} \left(1 - \text{logit}^{-1}\left[\tilde{\gamma}_0 + \tilde{\gamma}_{A2} + \tilde{\gamma}_{A3} \cdot k\right]\right).$$

Let us define the function `marginal.survivals` which, from given coefficients (`gammas`) and time-visit poits (`time.points`), computes the marginal survival probabilities for the *never treated* and *always treated* group.

```
marginal.survivals <- function(gammas, time.points){

  haz0 = expit(gammas[1] + gammas[2]*time.points)
  haz1 = expit(gammas[1] + gammas[3] + gammas[4]*time.points)
  surv0 = cumprod(1-haz0)
  surv1 = cumprod(1-haz1)

  return(list('never' = surv0, 'always' = surv1))
}
```

Use the defined function to compute the true marginal survival probabilities and the ones estimated from estimated coefficients under NoWT and WT 1-99.

```
time_points = seq(0, K.visits, by=0.1)
true_marg_surv = marginal.survivals(gammas = c(-3, 0.05, -1.5, 0.1), time_points)
marg_surv = marginal.survivals(gammas = est_coefs, time_points)
marg_surv_wt = marginal.survivals(gammas = est_coefs_wt, time_points)

par(mfrow=c(1,2))
plot(time_points, marg_surv$never, type='l', ylim = c(0,1), lty = 2, lwd = 2,
     xlab = 'Time-visit k', ylab = 'Marginal survival probabilities',
     main = 'No Weight Truncation')
points(time_points, marg_surv$always, type = 'l', lty = 1, lwd = 2)
points(time_points, true_marg_surv$never, type='l', lty = 2, lwd = 2, col='#FF6633')
points(time_points, true_marg_surv$always, type = 'l', lty = 1, lwd = 2, col='#FF6633')
legend(x=10, y=1,  legend = c("Never-treated", "Always-treated", "True values"),
       col = c("black", "black","#FF6633"), lty = c(2,1,1), bty = "n")

plot(time_points, marg_surv_wt$never, type='l', ylim = c(0,1), lty = 2, lwd = 2,
     xlab = 'Time-visit k', ylab = 'Marginal survival probabilities',
     main = 'Weight Truncation 1-99')
points(time_points, marg_surv_wt$always, type = 'l', lty = 1, lwd = 2)
points(time_points, true_marg_surv$never, type='l', lty = 2, lwd = 2, col='#FF6633')
points(time_points, true_marg_surv$always, type = 'l', lty = 1, lwd = 2, col='#FF6633')
legend(x=10, y=1,  legend = c("Never-treated", "Always-treated", "True values"),
       col = c("black", "black","#FF6633"), lty = c(2,1,1), bty = "n")
```

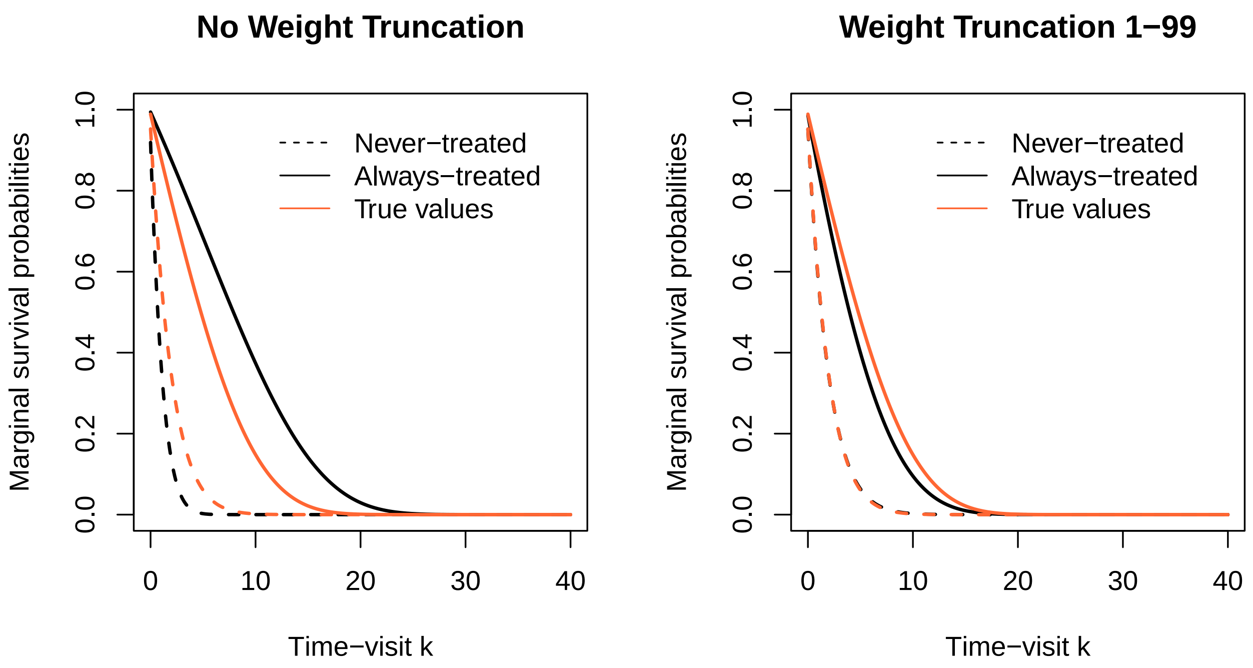


### I.b Examples for $B$ datasets simulated within a single $(n, WT, \pi, \tau)$ scenario

Now we want to perform the same analysis under WT 1-99, that is scenario

$$n = 300, \quad WT = 1 - 99, \quad \pi = 0.15, \quad \tau = 400,$$

for $B = 50$ repetitions. To this purpose, we can use the function `mc.sim.algI` from file *functions/mc_simI_functions.R*, as follows

```
source("functions/mc_simI_functions.R")

set.seed(5678)
result = mc.sim.algI(B = 50, K=K.visits, kappa=k.checkup,
                     pi.compliance = 0.15, tau.rule = 400, n.size = 300,
                     trunc = TRUE, trunc.percentiles = c(1,99))
```

For each repetition $b = 1, \ldots, 50$, the function first simulate a dataset $\mathcal{D}^b$ using Algorithm I, then computes the IPTW weights $\widehat{sw}_i^b(t)$ and finally estimate the logit-MSM by weighted logistic regression $(\widehat{\gamma}_0^b, \widehat{\gamma}_{A1}^b, \widehat{\gamma}_{A2}^b, \widehat{\gamma}_{A3}^b)$. At the end of each repetition, the simulated dataset and the individual estimated weights are discarded.

**IPTW weights.** For each repetition $b = 1, \ldots, B$, the within-dataset summary measures (mean, sd, max, min) of the estimated standardized IPTW weights $\widehat{sw}_i^b(t)$ computed across individuals $(i = 1, \ldots, 300)$ are given in `result$weights`.

```
head(result$weights)
```

```
##   rep_b   WT   pi tau   n visit   mean_sw     sd_sw     min_sw   max_sw
## 1     1 1-99 0.15 400 300     0 0.9033448 1.0268922 0.38207988 9.138151
## 2     1 1-99 0.15 400 300     5 0.8309057 0.9586861 0.17069002 9.138151
## 3     1 1-99 0.15 400 300    10 0.8834087 1.2073539 0.08761932 9.138151
## 4     1 1-99 0.15 400 300    15 0.8507560 1.0534497 0.05078070 9.138151
## 5     1 1-99 0.15 400 300    20 0.8749678 1.3311141 0.03272593 9.138151
## 6     1 1-99 0.15 400 300    25 0.8782367 1.5936757 0.03272593 9.138151
```

Let us compute the logarithm of the within-dataset mean, maximum, and minimum values

```
df_ipw = data.table(result$weights)
df_ipw[, log_mean_sw := log(mean_sw)]
df_ipw[, log_max_sw := log(max_sw)]
df_ipw[, log_min_sw := log(min_sw)]
```

and visualize the results over check-up visits:

```
par(mfrow=c(1,3))
# Log Mean
boxplot(log_mean_sw ~ visit, df_ipw[visit %in% check_ups], ylim = c(-5,5),
        xlab = 'Time-visit k', ylab = 'Log Mean Std. Weights', main = 'Log Mean')
abline(h=0, col='gray30', lty = 2)
# Log Max
boxplot(log_max_sw ~ visit, df_ipw[visit %in% check_ups], ylim = c(-5,5),
        xlab = 'Time-visit k', ylab = 'Log Max Std. Weights', main = 'Log Max')
abline(h=0, col='gray30', lty = 2)
abline(h=3, col='red', lty = 2)
# Log Min
boxplot(log_min_sw ~ visit, df_ipw[visit %in% check_ups], ylim = c(-5,5),
        xlab = 'Time-visit k', ylab = 'Log Min Std. Weights', main = 'Log Min')
abline(h=0, col='gray30', lty = 2)
abline(h=-5, col='red', lty = 2)
```

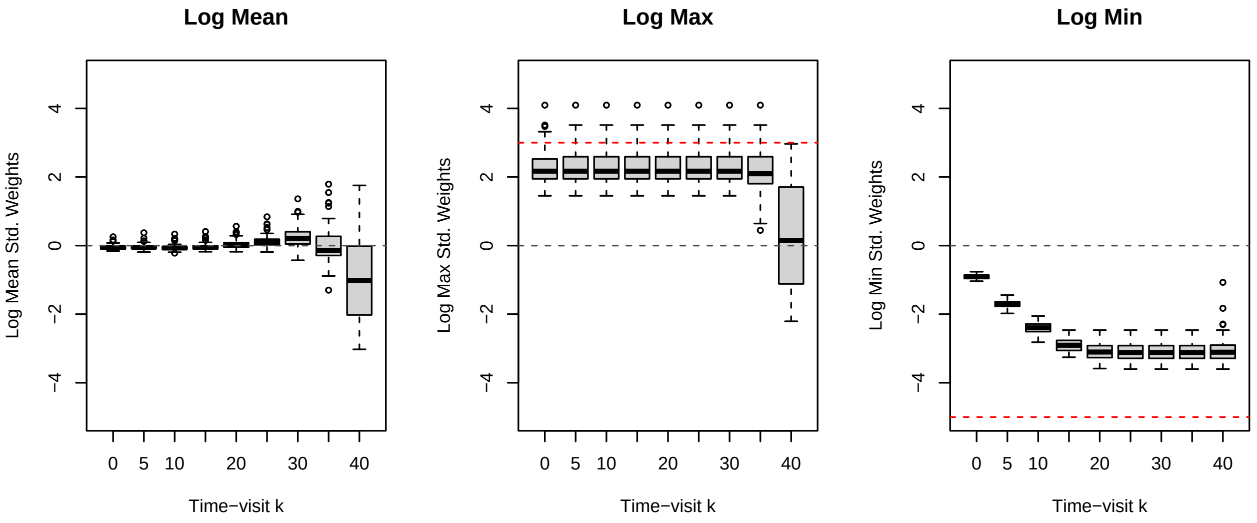


**Regression coefficients.** The estimasted regression coefficients $(\hat{\gamma}^b_0, \hat{\gamma}^b_{A1}, \hat{\gamma}^b_{A2}, \hat{\gamma}^b_{A3})$ for each repetition $b$ are given in `result$coef_est`

```
head(result$coef_est)
```

```
##   rep_b   WT   pi tau   n    gamma0     gammaA1    gammaA2    gammaA3
## 1     1 1-99 0.15 400 300 -2.978660 0.034480665 -1.2747774 0.09372310
## 2     2 1-99 0.15 400 300 -4.301139 0.048290954 -0.5800787 0.13145804
## 3     3 1-99 0.15 400 300 -3.128574 0.046344708 -1.5660689 0.11031263
## 4     4 1-99 0.15 400 300 -2.634837 0.004656646 -1.8543826 0.09926546
## 5     5 1-99 0.15 400 300 -4.208505 0.009968947 -0.5433567 0.11237498
## 6     6 1-99 0.15 400 300 -2.740924 0.006484770 -1.7993694 0.09789450
```

For a given coefficient (`coef.name`) with true value `true`, we can now use the function `eval.resultsI` from file *functions/eval_measuresI.R* to compute the estimation errors (`$errors`) across repetitions with the

relative performance (`$eval`).

```
source("functions/eval_measuresI.R")

# Evaluating performance
gamma0 = eval.resultsI(result$coef_est, coef.name='gamma0', true = -3)
gammaA1 = eval.resultsI(result$coef_est, coef.name='gammaA1', true = 0.05)
gammaA2 = eval.resultsI(result$coef_est, coef.name='gammaA2', true = -1.5)
gammaA3 = eval.resultsI(result$coef_est, coef.name='gammaA3', true = 0.1)
```

For example, for coefficient $\gamma_0$, the estimation errors over repetitions are given in `gamma0$errors`

```
head(gamma0$errors)
```

```
##    rep_b   WT   pi tau   n    gamma0       error
## 1:     1 1-99 0.15 400 300 -2.978660  0.02133968
## 2:     2 1-99 0.15 400 300 -4.301139 -1.30113934
## 3:     3 1-99 0.15 400 300 -3.128574 -0.12857385
## 4:     4 1-99 0.15 400 300 -2.634837  0.36516348
## 5:     5 1-99 0.15 400 300 -4.208505 -1.20850549
## 6:     6 1-99 0.15 400 300 -2.740924  0.25907629
```

with relative performance measures given in `gamma0$eval`.

Let us visualize the boxplots of the estimation errors of each regression coefficient across the simulated datasets.

```
library(latex2exp)
par(mfrow=c(1,4))
boxplot(gamma0$errors$error, ylab = 'Error', ylim = c(-2,2),
        main = expression(hat(gamma)[0]^"b" - tilde(gamma)[0]^"*"))
abline(h=0, col='blue', lty = 2)
boxplot(gammaA2$errors$error, ylim = c(-2,2),
        main = expression(hat(gamma)[A2]^"b" - tilde(gamma)[A2]^"*"))
abline(h=0, col='blue', lty = 2)

boxplot(gammaA1$errors$error, ylab = 'Error',ylim = c(-0.15,0.1),
        main = expression(hat(gamma)[A1]^"b" - tilde(gamma)[A1]^"*"))
abline(h=0, col='blue', lty = 2)
boxplot(gammaA3$errors$error, ylim = c(-0.15,0.1),
        main = expression(hat(gamma)[A3]^"b" - tilde(gamma)[A3]^"*"))
abline(h=0, col='blue', lty = 2)
```

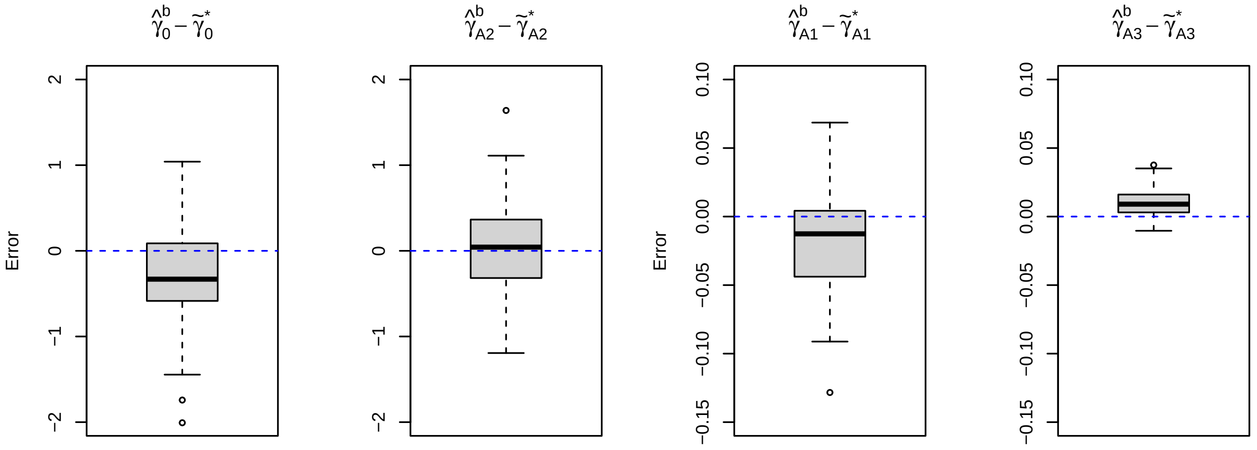

Mean estimated coefficients and relative performance measures in terms of estimated bias, empirical Standard Error (empSE), and Root Mean Squared Error (RMSE) are:

```
table.1 = cbind(Coefficient = c("gamma0","gammaA1","gammaA2","gammaA3"),
                True = c(-3,0.05,-1.5,0.1),
                rbind(gamma0$eval[,.(meanG,Bias,empSE,RMSE)],
                      gammaA1$eval[,.(meanG,Bias,empSE,RMSE)],
                      gammaA2$eval[,.(meanG,Bias,empSE,RMSE)],
                      gammaA3$eval[,.(meanG,Bias,empSE,RMSE)]))
table.1
```

```
##    Coefficient  True       meanG         Bias      empSE       RMSE
## 1:      gamma0 -3.00 -3.33341698 -0.333416976 0.58595137 0.66905838
## 2:     gammaA1  0.05  0.03170745 -0.018292551 0.03931282 0.04300239
## 3:     gammaA2 -1.50 -1.41184165  0.088158353 0.55640926 0.55782733
## 4:     gammaA3  0.10  0.10957826  0.009578259 0.01073630 0.01430754
```

**Marginal Survival Curves.** We want now to retrieve the estimated marginal survival curves for *always treated* and *never treated* groups for each simulated dataset $b = 1, \dots, B$. To this purpose we can use the Function `marginal.surv.probI` from from file *functions/eval_measuresI.R*, as follows

```
surv_est = marginal.surv.probI(result$coef_est, times = time_points)
head(surv_est)
```

```
##    rep_b   WT   pi tau   n time       haz0       haz1     surv0     surv1 nsim
## 1:     1 1-99 0.15 400 300  0.0 0.04839929 0.01401604 0.9516007 0.9859840   50
## 2:     1 1-99 0.15 400 300  0.1 0.04855835 0.01414615 0.9053925 0.9720361   50
## 3:     1 1-99 0.15 400 300  0.2 0.04871790 0.01427746 0.8612837 0.9581579   50
## 4:     1 1-99 0.15 400 300  0.3 0.04887795 0.01440996 0.8191859 0.9443509   50
## 5:     1 1-99 0.15 400 300  0.4 0.04903849 0.01454368 0.7790143 0.9306165   50
## 6:     1 1-99 0.15 400 300  0.5 0.04919954 0.01467862 0.7406872 0.9169564   50
```

Let us display the true survival curve (in orange), the estimated curves for each dataset (in grey), and their mean (in yellow).

```
true_curve_data = data.frame(time = time_points,
                             true_surv0 = true_marg_surv$never,
                             true_surv1 = true_marg_surv$always)

avg_curves = surv_est[, lapply(.SD, mean), by = time, .SDcols = c("surv0", "surv1")]

library(ggplot2)
library(ggpubr)
p.never = ggplot(surv_est, aes(x=time, y=surv0, group=factor(rep_b))) +
  geom_line(alpha=0.8, linetype='dashed', col='gray30') + theme_light() +
  geom_line(data = true_curve_data, aes(x=time, y=true_surv0, group=NULL),
            color = "#FF6633", linetype = "dashed", linewidth = 1) +
  geom_line(data = avg_curves, aes(x=time, y=surv0, group=NULL),
            color = "#FFCC33", linetype = "dashed", linewidth = 1) +
  labs(x='Time-visit k', y='Marginal Survival Probability', title = 'Never treated')

p.always = ggplot(surv_est, aes(x=time, y=surv1, group=factor(rep_b))) +
  geom_line(alpha=0.8, linetype='solid', col='gray30') + theme_light() +
  geom_line(data = true_curve_data, aes(x=time, y=true_surv1, group=NULL),
            color = "#FF6633", linetype = "solid", linewidth = 1) +
  geom_line(data = avg_curves, aes(x=time, y=surv1, group=NULL),
            color = "#FFCC33", linetype = "solid", linewidth = 1) +
```

```
  labs(x='Time-visit k', y='Marginal Survival Probability', title = 'Always treated')

ggarrange(p.never, p.always)
```

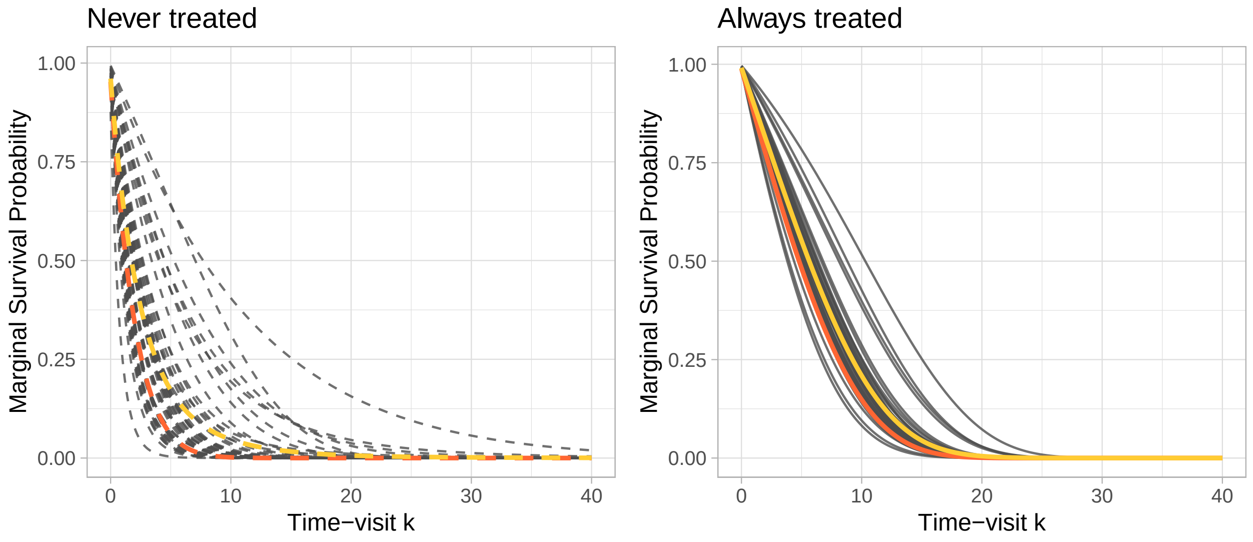

# II. Analysis with Algorithm II

## II.a Example of a single simulated dataset

**Step 1 | Simulate data.** Algorithm II (see Manuscript Section 5.1.2) is implemented in the function `sim.algorithmII`, available in the file *functions/algorithm_II.R*.

Using this function, let us simulate a dataset of $n = 300$ subjects (`n`) over $K = 4$ time-points with administrative censoring at $K + 1$ (`K`), considering an exposure cut-off of $\pi = 0.15$ (`pi.prop`) and a poor-health subgroup $\mathcal{I}_\tau = (\tau, \infty)$ with threshold $\tau = 1$ (`tau`). Note that parameter `K` is the total number of visits over time-points $k = 0, 1, \ldots, K$ including baseline so it is $K + 1$.

*Remark.* As mentioned in Manuscript Section 5.2.1, by default the data are simulated in such a way that the true values of the conditional distribution parameters are $(\alpha_0, \alpha_A, \alpha_L, \alpha_U) = (0.7, -0.2, 0.05, 0.05)$

```
source("functions/algorithm_II.R")
K.visits = 5

set.seed(1234)
df = sim.algorithmII(pi.prop = 0.05, tau = 1, n = 300, K=K.visits)
df[1:9,]
```

```
##     id      T.obs Y.obs            U time A           L Alag1 Alag2 Alag3 Alag4
## 1.0  1 0.02427997     1 -0.037723765    0 0  0.32494319     0     0     0     0
## 2.0  2 3.92865949     1  0.009761946    0 1  1.42116659     0     0     0     0
## 2.1  2 3.92865949     1  0.009761946    1 0 -0.17984723     1     0     0     0
## 2.2  2 3.92865949     1  0.009761946    2 0  0.13734677     0     1     0     0
## 2.3  2 3.92865949     1  0.009761946    3 0 -0.07886111     0     0     1     0
## 3.0  3 0.40089287     1  0.163874465    0 1  1.53141878     0     0     0     0
## 4.0  4 0.08574960     1 -0.087559247    0 0 -0.49473539     0     0     0     0
## 5.0  5 1.84507761     1  0.012176000    0 1  0.77471773     0     0     0     0
## 5.1  5 1.84507761     1  0.012176000    1 1 -0.71064933     1     0     0     0
##      time.stop event
## 1.0 0.02427997     1
## 2.0 1.00000000     0
## 2.1 2.00000000     0
## 2.2 3.00000000     0
## 2.3 3.92865949     1
## 3.0 0.40089287     1
## 4.0 0.08574960     1
## 5.0 1.00000000     0
## 5.1 1.84507761     1
```

The dataset contains:

- `id`: subject index $i$
- `T.obs`: time to event or censoring $T_i^* = \min(C_i, T_i)$
- `Y.obs`: event/censoring indicator $I(T_i \le C_i)$
- `U`: baseline latent variable $U_i$
- `time`: start visit time $k$
- `A`: observed binary treatment $A_{i,k}$
- `L`: observed biomarker value $L_{i,k}$
- `Alag1`: observed binary treatment at the previous time $A_{i,k-1}$

- `Alag2`: observed binary treatment with lag 2 $A_{i,k-2}$
- `Alag3`: observed binary treatment with lag 3 $A_{i,k-3}$
- `Alag4`: observed binary treatment with lag 4 $A_{i,k-4}$
- `time.stop`: stop visit time, that is $k \leq T_i < k+1$ if the subject experienced the event or $k+1$ otherwise
- `event`: indicator $Y_{i,k+1} = I(k < T_i \leq k+1)$ of whether the event occurs between visits $k$ and $k+1$

**Step 2 | Compute IPTW weights.** As mentioned in Manuscript Section 5.2.1, weight components at time $k$ are estimated by logistic regression models for the probability of being exposed at time $k$, with numerator

$$\Pr\left(A_{i,k} = 1 \mid \bar{\boldsymbol{A}}_{i,k-1}, T_i \geq k\right) = logit^{-1}\left[\theta_0 + \theta_1 \cdot A_{i,k-1}\right]$$

and denominator

$$\Pr\left(A_{i,k} = 1 \mid \bar{\boldsymbol{A}}_{i,k-1}, \bar{\boldsymbol{L}}_{i,k}, T_i \geq k\right) = logit^{-1}\left[\theta_0 + \theta_1 \cdot A_{i,k-1} + \theta_2 \cdot L_{i,k}\right].$$

The stabilized weight for subject $i$ at time $t$ is then defined as:

$$sw_i(t) = \prod_{k=0}^{\lfloor t \rfloor} \frac{\Pr\left(A_{i,k} \mid \bar{\boldsymbol{A}}_{i,k-1}, T_i \geq k\right)}{\Pr\left(A_{i,k} \mid \bar{\boldsymbol{A}}_{i,k-1}, \bar{\boldsymbol{L}}_{i,k}, T_i \geq k\right)}$$

where $\lfloor t \rfloor$ is the largest time-visit prior to $t$.

The function `get.std.weightsII` from file *functions/iptw_II.R* computes the IPTW standardized weights $sw_i(t)$. By default, stabilized weights are computed without weight truncation (WT).

```
source("functions/iptw_II.R")

# No Weight Truncation (NoWT)
df_sw = get.std.weightsII(df, trunc = FALSE)
summary(df_sw$sw)
```

```
##      Min.   1st Qu.    Median      Mean   3rd Qu.      Max.
##     0.025     0.505     0.814    47.400     0.980 28058.859
```

To apply WT to 1st and 9th percentiles (WT 1-99), parameters `trunc` must be `TRUE` and the desired lower and upper percentiles must be specified in parameter `percentiles`, as follows:

```
# Weight Truncation 1st-99th percentiles (WT 1-99)
df_sw_wt = get.std.weightsII(df, trunc = TRUE, percentiles = c(1,99))
summary(df_sw_wt$sw)
```

```
##    Min. 1st Qu.  Median    Mean 3rd Qu.    Max.
## 0.08755 0.50543 0.81362 1.05233 0.97972 9.24435
```

The logarithm of estimated standardized weights $\log\left(\widehat{sw}_i(t)\right)$ can be used to check the distributions of the weights times:

```
par(mfrow = c(1, 2))
boxplot(log(sw) ~ time, data = df_sw,
        xlab = 'Time t', ylab = 'Logarithm of standardized weights',
        main = 'No Weight Truncation')
boxplot(log(sw) ~ time, data = df_sw_wt,
        xlab = 'Time t', ylab = 'Logarithm of standardized weights',
        main = 'Weight Truncation 1-99', ylim=range(log(df_sw$sw)))
```

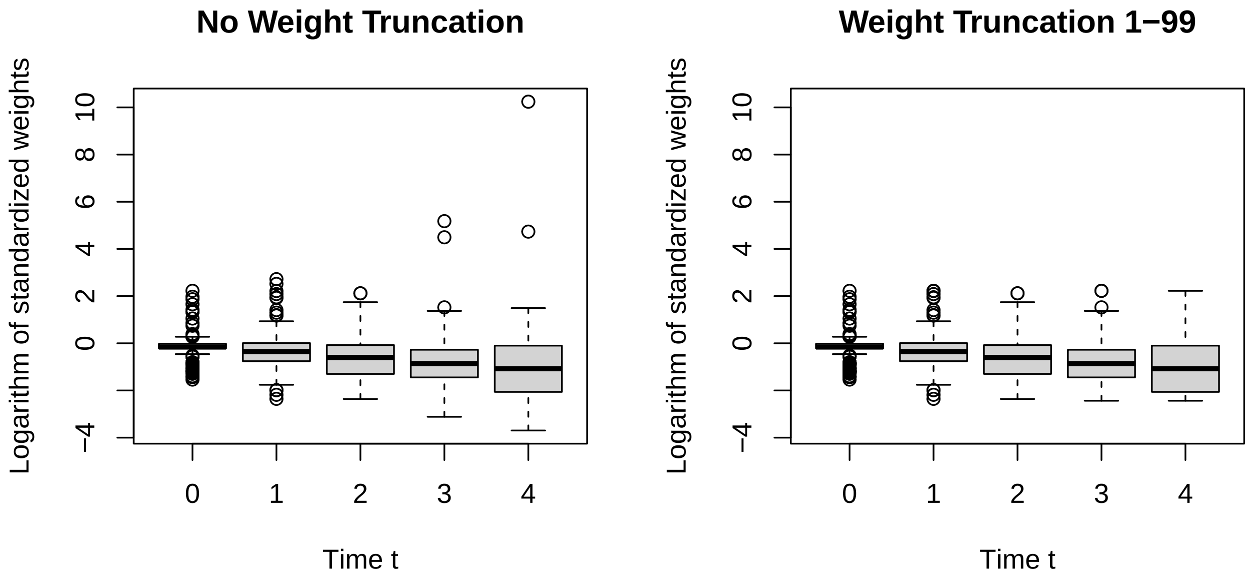


**Step 3 | Estimate Aalen-MSM.** The desired marginal structural Aalen's additive hazard model (Aalen-MSM) $\bar{\boldsymbol{a}}$ at time $t$ (see Manuscript Section 5.1.1) is

$$\lambda^{\bar{\boldsymbol{a}}}(t) = \tilde{\alpha}_0(t) + \sum_{j=0}^{\lfloor t \rfloor} \tilde{\alpha}_{Aj}(t) \cdot a_{\lfloor t \rfloor - j}$$

which can be estimated by appling IPTW to the following conditional additive hazard model

$$\lambda_i \left( t \mid \bar{\boldsymbol{A}}_{i,\lfloor t \rfloor}, \bar{\boldsymbol{L}}_{i,\lfloor t \rfloor}, U_i \right) = \alpha_0 + \alpha_A \cdot A_{i,\lfloor t \rfloor} + \alpha_L \cdot L_{i,\lfloor t \rfloor} + \alpha_U \cdot U_i.$$

This can be done by employing the functions `aalen.MSM`, `cum.coef.aalen`, and `extract.cum.coef` from file *functions/mc_simII_functions.R*.

The estimates of the cumulative regression coefficients

$$C_0(t) = \int_0^t \tilde{\alpha}_0(s) ds \quad \text{and} \quad C_{Aj}(t) = \int_0^t \tilde{\alpha}_{Aj}(s) ds \quad (j = 0, \ldots, 4)$$

over time $t = 1, \ldots, 5$ under WT are

```
source("functions/mc_simII_functions.R")
```

```
## Loading required package: survival
```

```
MSMmodel = aalen.MSM(df_sw, K.visits)
cum_coefs = cum.coef.aalen(MSMmodel, t.hor=seq(0,K.visits,0.01))
cum_coefs_t15 = extract.cum.coef(cum_coefs, K.visits)
cum_coefs_t15[is.na(cum_coefs_t15)] = 0
cum_coefs_t15
```

```
##             t1         t2         t3          t4          t5
## C0   0.6721644  1.3165961  2.1100850  2.16972190  2.16974930
## CA0 -0.4440846 -0.6752067 -1.0538338 -1.56135850 -1.50660497
## CA1  0.0000000 -0.1656435  0.5983235  1.06425945  1.61765355
## CA2  0.0000000  0.0000000 -0.4745750  0.39523202 -0.15816350
## CA3  0.0000000  0.0000000  0.0000000 -0.07773807  1.44358241
## CA4  0.0000000  0.0000000  0.0000000  0.00000000  0.03804083
```

and under WT 1-99:

```
MSMmodel_wt = aalen.MSM(df_sw_wt, K.visits)
cum_coefs_wt = cum.coef.aalen(MSMmodel_wt, t.hor=seq(0,K.visits,0.01))
cum_coefs_wt_t15 = extract.cum.coef(cum_coefs_wt, K.visits)
cum_coefs_wt_t15[is.na(cum_coefs_wt_t15)] = 0
cum_coefs_wt_t15
```

```
##             t1         t2         t3         t4           t5
## C0   0.6721644  1.3068483  2.1003372  2.4393310  2.503991868
## CA0 -0.4440449 -0.7203079 -1.0989350 -1.7157740 -1.653316336
## CA1  0.0000000 -0.1307712  0.6331958  1.1657239  1.639198782
## CA2  0.0000000  0.0000000 -0.4745750  0.3133559 -0.225586684
## CA3  0.0000000  0.0000000  0.0000000 -0.3217845  1.389048185
## CA4  0.0000000  0.0000000  0.0000000  0.0000000 -0.007876851
```

while the true values are

```
load("results/algorithmII/true_cum_coefs.Rdata")
CCtrue = reshape(Ccoefs_true, idvar = "Ccoef", timevar = "time", direction = "wide")
colnames(CCtrue) = c('Ccoef',paste0('t',1:5))
CCtrue[is.na(CCtrue)] = 0
CCtrue
```

```
##    Ccoef     t1     t2     t3     t4     t5
## 1:    C0  0.700  1.408  2.128  2.863  3.623
## 2:   CA0 -0.198 -0.396 -0.594 -0.790 -0.987
## 3:   CA1  0.000 -0.098 -0.195 -0.291 -0.386
## 4:   CA2  0.000  0.000 -0.077 -0.153 -0.228
## 5:   CA3  0.000  0.000  0.000 -0.060 -0.121
## 6:   CA4  0.000  0.000  0.000  0.000 -0.047
```

**Step 4 | Marginal Survival Curves.** For Aalen-MSMs, the marginal survival probability at time $t$ under treatment history $\bar{\boldsymbol{a}}$ is defined as

$$S^{\bar{\boldsymbol{a}}}(t) = \exp\left( - \int_0^t \tilde{\alpha}_0(s)\, ds - \int_0^1 g(\tilde{\boldsymbol{\alpha}}_A(s); a_0)\, ds - \int_1^2 g(\tilde{\boldsymbol{\alpha}}_A(s); \bar{\boldsymbol{a}}_1)\, ds - \cdots - \int_{\lfloor t \rfloor}^t g(\tilde{\boldsymbol{\alpha}}_A(s); \bar{\boldsymbol{a}}_{\lfloor t \rfloor})\, ds \right).$$

For the desired Aalen-MSM with $g\left(\tilde{\boldsymbol{\alpha}}_A(t); \bar{\boldsymbol{a}}_{\lfloor t \rfloor}\right) = \sum_{j=0}^{\lfloor t \rfloor} \tilde{\alpha}_{Aj}(t) \cdot a_{\lfloor t \rfloor - j}$, the marginal survival probabilities for the *never treated* ($\bar{\boldsymbol{a}} = \bar{\mathbf{0}}$) and *always treated* ($\bar{\boldsymbol{a}} = \bar{\mathbf{1}}$) groups are:

$$S^{\bar{\mathbf{0}}}(t) = \exp\left( - \int_0^t \tilde{\alpha}_0(s)\, ds \right) = \exp\big( - C_0(t) \big)$$

$$S^{\bar{\mathbf{1}}}(t) = \exp\left( - C_0(t) - \sum_{j=0}^{\lfloor t \rfloor - 1} \left[ C_{Aj}(t) - C_{Aj}(j) \right] - \left[ C_{A\lfloor t \rfloor}(t) - C_{A\lfloor t \rfloor}(\lfloor t \rfloor) \right] \right).$$

Function `marginal.surv.probII` from file *functions/mc_simII_functions.R* compute the marginal survival probabilities over times `t.hor` in the *always treated* and *never treated* groups

```
time_points = seq(0, K.visits, by=0.1)
marg_surv = marginal.surv.probII(MSMmodel, time_points)
marg_surv_wt = marginal.surv.probII(MSMmodel_wt, time_points)

# True values
load("results/algorithmII/true_survivals.Rdata")
```

```r
par(mfrow=c(1,2))
plot(time_points, marg_surv$surv0, type='l', ylim = c(0,1), lty = 2, lwd = 2,
     xlab = 'Time t', ylab = 'Marginal survival probabilities',
     main = 'No Weight Truncation')
points(time_points, marg_surv$surv1, type = 'l', lty = 1, lwd = 2)
points(true_surv$time, true_surv$true_surv0, type='l', lty = 2, lwd = 2, col='#FF6633')
points(true_surv$time, true_surv$true_surv1, type = 'l', lty = 1, lwd = 2, col='#FF6633')
legend(x=10, y=1,  legend = c("Never-treated", "Always-treated", "True values"),
       col = c("black", "black","#FF6633"), lty = c(2,1,1), bty = "n")

plot(time_points, marg_surv_wt$surv0, type='l', ylim = c(0,1), lty = 2, lwd = 2,
     xlab = 'Time t', ylab = 'Marginal survival probabilities',
     main = 'Weight Truncation 1-99')
points(time_points, marg_surv_wt$surv1, type = 'l', lty = 1, lwd = 2)
points(true_surv$time, true_surv$true_surv0, type='l', lty = 2, lwd = 2, col='#FF6633')
points(true_surv$time, true_surv$true_surv1, type = 'l', lty = 1, lwd = 2, col='#FF6633')
legend(x=10, y=1,  legend = c("Never-treated", "Always-treated", "True values"),
       col = c("black", "black","#FF6633"), lty = c(2,1,1), bty = "n")
```

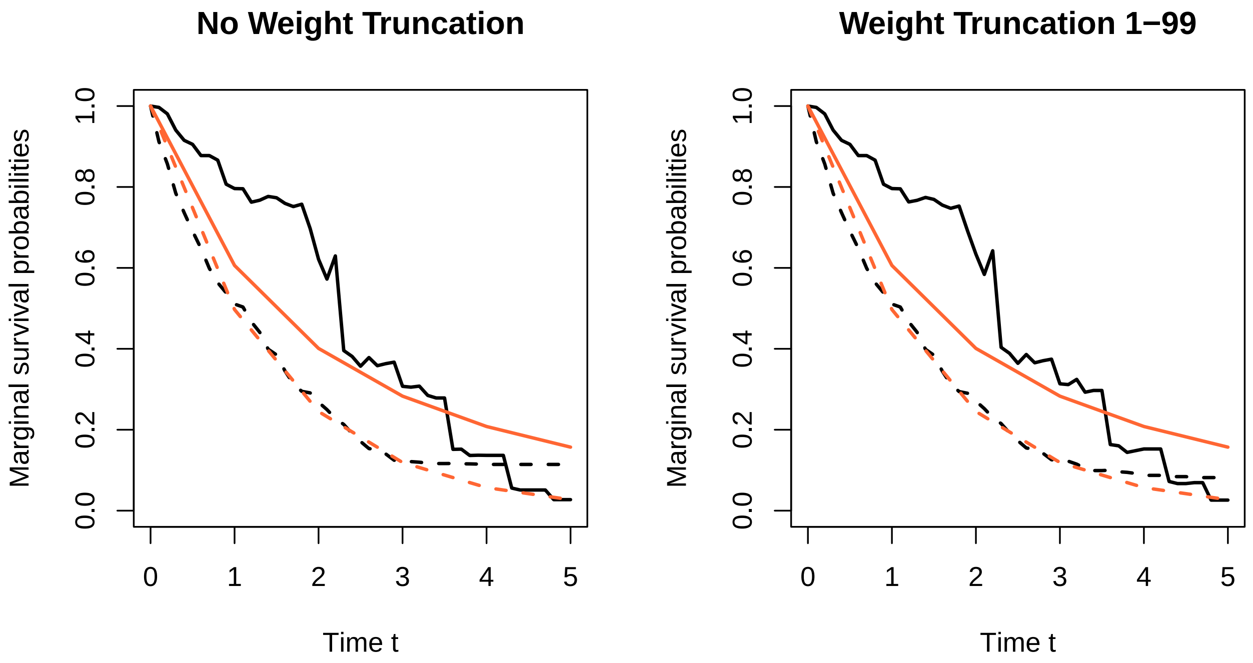


### II.b Examples for $B$ datasets simulated within a single $(n, WT, \pi, \tau)$ scenario

Now we want to perform the same analysis under WT 1-99, that is scenario

$$n = 300, \quad WT = 1 - 99, \quad \pi = 0.15, \quad \tau = 1,$$

for $B = 50$ repetitions. To this purpose, we can use the function `mc.sim.algII` from file *functions/mc_simII_functions.R*, as follows

```r
source("functions/mc_simII_functions.R")

set.seed(1234)
```

```r
result = mc.sim.algII(B = 50, K=K.visits,
                      pi.compliance = 0.15, tau.rule = 1, n.size = 300,
                      trunc = TRUE, trunc.percentiles = c(1,99))
```

For each repetition $b = 1, \ldots, 50$, the function first simulate a dataset $\mathcal{D}^b$ using Algorithm II, then computes the IPTW weights $\widehat{sw}_i^b(t)$ and finally estimate the cumulative regression coefficients $\widehat{C}_0^b(t) = \int_0^t \tilde{\alpha}_0(s)ds$ and $\widehat{C}_{Aj}^b(t) = \int_0^t \tilde{\alpha}_{Aj}(s)ds$ $(j = 0, \ldots, 4)$ by weighted logistic Aalen-regression and the marginal survival probabilities $\{\widehat{S}^{\bar{a}}(t)\}^b$. At the end of each repetition, the simulated dataset and the individual estimated weights are discarded.

**IPTW weights.** For each repetition $b = 1, \ldots, B$, the within-dataset summary measures (mean, sd, max, min) of the estimated standardized IPTW weights $\widehat{sw}_i^b(t)$ computed across individuals $(i = 1, \ldots, 300)$ are given in `result$weights`.

```r
head(result$weights)
```

```
##    rep_b   WT   pi tau   n time   mean_sw     sd_sw     min_sw    max_sw
## 1:     1 1-99 0.15   1 300    0 0.9805570 0.7870408 0.20848994  8.351214
## 2:     1 1-99 0.15   1 300    1 1.0856299 1.4117730 0.09750394  8.548420
## 3:     1 1-99 0.15   1 300    2 1.0734478 1.4238097 0.09195479  7.685142
## 4:     1 1-99 0.15   1 300    3 1.1754543 2.0071371 0.09195479  8.548420
## 5:     1 1-99 0.15   1 300    4 1.6042542 2.6198265 0.09195479  8.548420
## 6:     2 1-99 0.15   1 300    0 0.9915949 1.0160340 0.23367149 12.555474
```

Let us compute the logarithm of the within-dataset mean, maximum, and minimum values

```r
df_ipw = data.table(result$weights)
df_ipw[, log_mean_sw := log(mean_sw)]
df_ipw[, log_max_sw := log(max_sw)]
df_ipw[, log_min_sw := log(min_sw)]
```

and visualize the results over check-up visits:

```r
par(mfrow=c(1,3))
# Log Mean
boxplot(log_mean_sw ~ time, df_ipw, ylim = c(-5,5),
        xlab = 'Time t', ylab = 'Log Mean Std. Weights', main = 'Log Mean')
abline(h=0, col='gray30', lty = 2)
# Log Max
boxplot(log_max_sw ~ time, df_ipw, ylim = c(-5,5),
        xlab = 'Time t', ylab = 'Log Max Std. Weights', main = 'Log Max')
abline(h=0, col='gray30', lty = 2)
abline(h=3, col='red', lty = 2)
# Log Min
boxplot(log_min_sw ~ time, df_ipw, ylim = c(-5,5),
        xlab = 'Time t', ylab = 'Log Min Std. Weights', main = 'Log Min')
abline(h=0, col='gray30', lty = 2)
abline(h=-5, col='red', lty = 2)
```

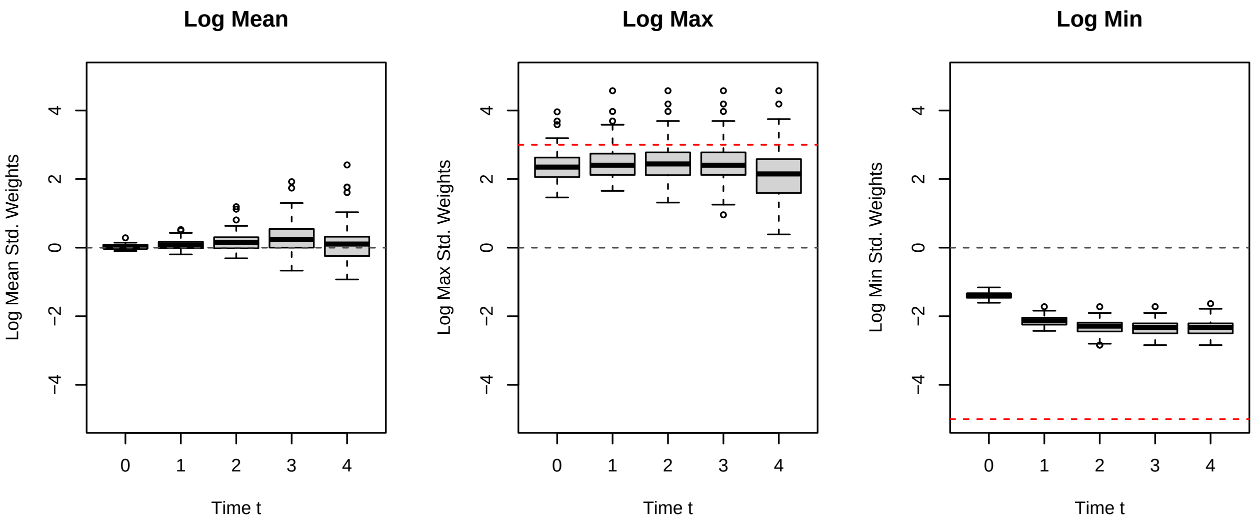


**Cumulative regression coefficients.** The estimasted regression coefficients $\widehat{C}_0^b(t) = \int_0^t \tilde{\alpha}_0(s)ds$ and $\widehat{C}_{Aj}^b(t) = \int_0^t \tilde{\alpha}_{Aj}(s)ds$ $(j = 0, \ldots, 4)$ for each repetition $b$ are given in `result$cum_coefs`

```
head(result$cum_coefs)
```

```
##     rep_b    WT   pi  tau    n time        C0         CA0        CA1        CA2
## 1:      1 1-99 0.15    1 300    1 0.6423817 -0.39932598         NA         NA
## 2:      1 1-99 0.15    1 300    2 1.2696765 -0.67865235 -0.0955894         NA
## 3:      1 1-99 0.15    1 300    3 2.1519279 -1.12707516  0.5983592 -0.5939495
## 4:      1 1-99 0.15    1 300    4 2.5010187 -1.74928082  1.1310761  0.1923705
## 5:      1 1-99 0.15    1 300    5 2.5567114 -1.63447151  1.5784301 -0.3138735
## 6:      2 1-99 0.15    1 300    1 0.7354737 -0.06866656         NA         NA
##            CA3       CA4
## 1:          NA        NA
## 2:          NA        NA
## 3:          NA        NA
## 4: -0.3239538         NA
## 5:  1.3679869 0.0074784
## 6:          NA        NA
```

*Warning.* `NA` values are equal to 0 by definition.

For a given coefficient (`coef.name`) with true values `true.df`, we can now use the function `eval.resultsII` from file *functions/eval_measuresII.R* to compute the estimation errors (`$errors`) across repetitions with the relative performance (`$eval`).

```
source("functions/eval_measuresII.R")

C0 = eval.resultsII(result$cum_coefs, coef.name='C0', true.df = Ccoefs_true)
CA0 = eval.resultsII(result$cum_coefs, coef.name='CA0', true.df = Ccoefs_true)
CA1 = eval.resultsII(result$cum_coefs, coef.name='CA1', true.df = Ccoefs_true)
CA2 = eval.resultsII(result$cum_coefs, coef.name='CA2', true.df = Ccoefs_true)
CA3 = eval.resultsII(result$cum_coefs, coef.name='CA3', true.df = Ccoefs_true)
CA4 = eval.resultsII(result$cum_coefs, coef.name='CA4', true.df = Ccoefs_true)
```

For example, for cumulative coefficient $C_0(t)$, the estimation errors across time $t$ over repetitions are given in `C0$errors`

```
head(C0$errors)
```

```
##    time rep_b   WT   pi tau   n        C0 true       error
## 1:    1     1 1-99 0.15   1 300 0.6423817  0.7 -0.05761829
## 2:    1     2 1-99 0.15   1 300 0.7354737  0.7  0.03547372
## 3:    1     3 1-99 0.15   1 300 0.6727198  0.7 -0.02728021
## 4:    1     4 1-99 0.15   1 300 0.6005304  0.7 -0.09946962
## 5:    1     5 1-99 0.15   1 300 0.6478036  0.7 -0.05219645
## 6:    1     6 1-99 0.15   1 300 0.7060715  0.7  0.00607146
```

with relative performance measures given in `C0$eval`.

Let us visualize the boxplots of the estimation errors over time of each regression coefficient across the simulated datasets.

```
library(latex2exp)
par(mfrow=c(2,3))
boxplot(C0$errors$error ~ C0$errors$time,
        xlab = 'Time t', ylab = 'Error', ylim = c(-3,3),
        main = expression(hat(C)[0]^"b"*(t) - tilde(C)[0]^"*"*(t)))
abline(h=0, col='blue', lty = 2)

boxplot(CA0$errors$error ~ CA0$errors$time,
        xlab = 'Time t', ylab = 'Error', ylim = c(-3,3),
        main = expression(hat(C)[A0]^"b"*(t) - tilde(C)[A0]^"*"*(t)))
abline(h=0, col='blue', lty = 2)

CA1$errors$time <- factor(CA1$errors$time, levels = 1:5)
boxplot(CA1$errors$error ~ CA1$errors$time,
        xlab = 'Time t', ylab = 'Error',  ylim = c(-3,3),
        main = expression(hat(C)[A1]^"b"*(t) - tilde(C)[A1]^"*"*(t)))
abline(h=0, col='blue', lty = 2)

CA2$errors$time <- factor(CA2$errors$time, levels = 1:5)
boxplot(CA2$errors$error ~ CA2$errors$time,
        xlab = 'Time t', ylab = 'Error',  ylim = c(-3,3),
        main = expression(hat(C)[A2]^"b"*(t) - tilde(C)[A2]^"*"*(t)))
abline(h=0, col='blue', lty = 2)

CA3$errors$time <- factor(CA3$errors$time, levels = 1:5)
boxplot(CA3$errors$error ~ CA3$errors$time,
        xlab = 'Time t', ylab = 'Error', ylim = c(-3,3),
        main = expression(hat(C)[A3]^"b"*(t) - tilde(C)[A3]^"*"*(t)))
abline(h=0, col='blue', lty = 2)

CA4$errors$time <- factor(CA4$errors$time, levels = 1:5)
boxplot(CA4$errors$error ~ CA4$errors$time,
        xlab = 'Time t', ylab = 'Error',  ylim = c(-3,3),
        main = expression(hat(C)[A4]^"b"*(t) - tilde(C)[A4]^"*"*(t)))
abline(h=0, col='blue', lty = 2)
```

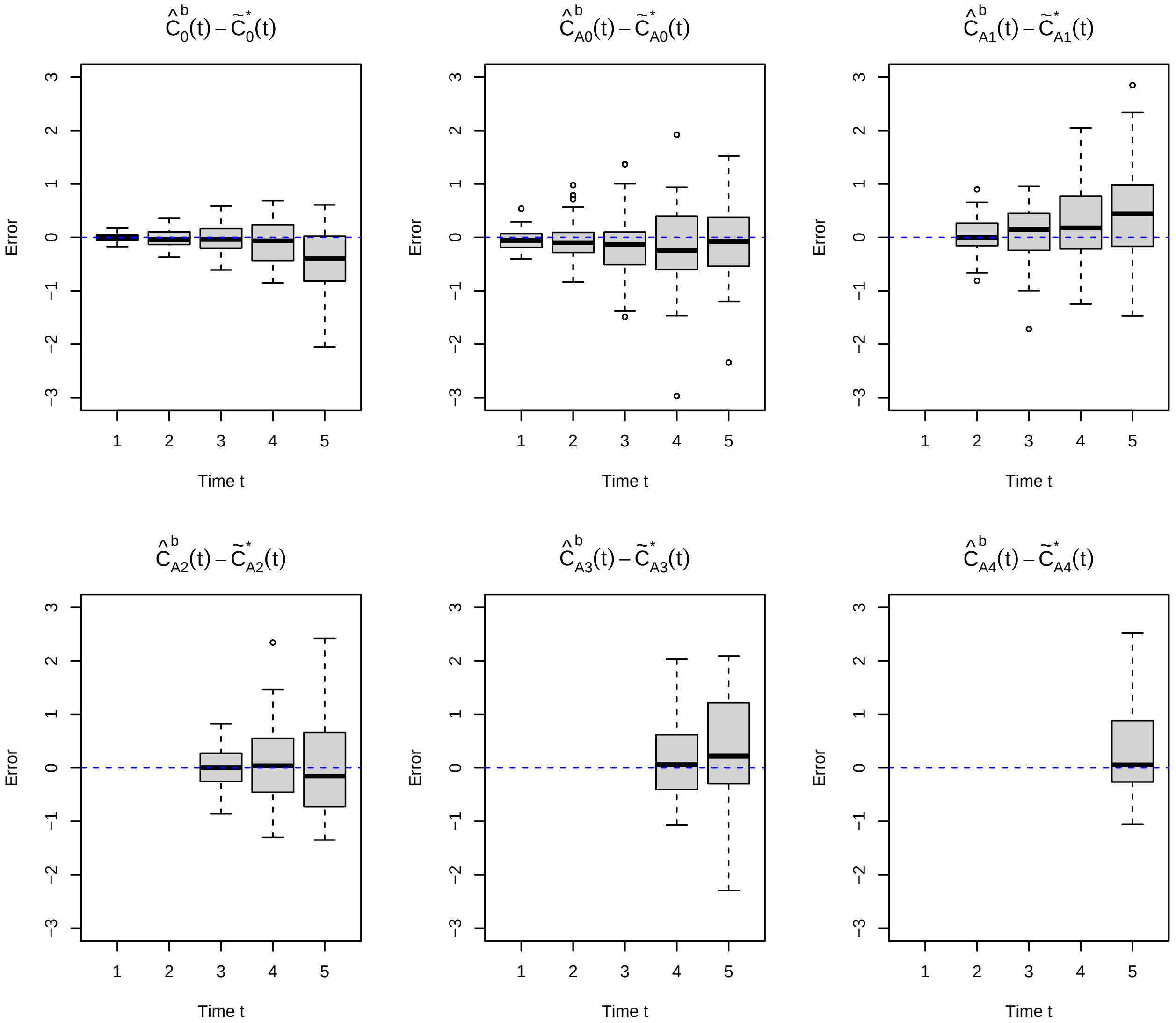


Mean estimated coefficients and relative performance measures over time in terms of estimated bias, empirical Standard Error (empSE), and Root Mean Squared Error (RMSE) are:

```
cum_coef_eval = rbind(cbind(Ccoef="C0", C0$eval),
                      cbind(Ccoef="CA0", CA0$eval),
                      cbind(Ccoef="CA1", CA1$eval),
                      cbind(Ccoef="CA2", CA2$eval),
                      cbind(Ccoef="CA3", CA3$eval),
                      cbind(Ccoef="CA4", CA4$eval))
cum_coef_eval = merge(cum_coef_eval, Ccoefs_true, by=c('Ccoef','time'))

table.23 = cum_coef_eval[,.(Ccoef,time,true,meanG,Bias,empSE,RMSE)]
table.23
```

```
##     Ccoef time  true      meanG          Bias      empSE       RMSE
##  1:    C0    1 0.700 0.70034110  0.0003410993 0.07758162 0.07680264
##  2:    C0    2 1.408 1.38620993 -0.0217900728 0.16471082 0.16450492
##  3:    C0    3 2.128 2.12294814 -0.0050518629 0.28102450 0.27824592
##  4:    C0    4 2.863 2.76140023 -0.1015997740 0.38563253 0.39504521
##  5:    C0    5 3.623 3.19545614 -0.4275438645 0.55284594 0.69449235
```

```
##  6:   CA0    1 -0.198 -0.24768639 -0.0496863880 0.18671254 0.19139770
##  7:   CA0    2 -0.396 -0.46056548 -0.0645654770 0.37377776 0.37561193
##  8:   CA0    3 -0.594 -0.75411188 -0.1601118793 0.55170608 0.56914657
##  9:   CA0    4 -0.790 -0.99240405 -0.2024040525 0.79791603 0.81541645
## 10:   CA0    5 -0.987 -1.02389868 -0.0368986803 0.93523868 0.92657405
## 11:   CA1    2 -0.098 -0.06764936  0.0303506397 0.32693517 0.32506928
## 12:   CA1    3 -0.195 -0.14319858  0.0518014156 0.49744311 0.49516062
## 13:   CA1    4 -0.291 -0.03200372  0.2589962751 0.69159966 0.73199929
## 14:   CA1    5 -0.386  0.06679314  0.4527931442 0.89909186 0.99860928
## 15:   CA2    3 -0.077 -0.06706603  0.0099339669 0.36167580 0.35817856
## 16:   CA2    4 -0.153 -0.09797244  0.0550275562 0.73583352 0.73051350
## 17:   CA2    5 -0.228 -0.17905995  0.0489400469 0.96566295 0.95720947
## 18:   CA3    4 -0.060  0.08896500  0.1489650014 0.68090746 0.69032807
## 19:   CA3    5 -0.121  0.21074117  0.3317411708 0.99216169 1.03670118
## 20:   CA4    5 -0.047  0.22104091  0.2680409138 0.78786609 0.82472071
```

**Marginal Survival Curves.** The estimated marginal survival curves for *always treated* (`surv1`) and *never treated* (`surv0`) groups for each simulated dataset $b = 1, \ldots, B$ for each repetition $b$ are given in `result$survivals`

```r
head(result$survivals)
```

```
##    rep_b   WT   pi tau   n time     surv0 surv1
## 1:     1 1-99 0.15   1 300 0.00 1.0000000     1
## 2:     1 1-99 0.15   1 300 0.01 0.9925913     1
## 3:     1 1-99 0.15   1 300 0.02 0.9806131     1
## 4:     1 1-99 0.15   1 300 0.03 0.9689484     1
## 5:     1 1-99 0.15   1 300 0.04 0.9564234     1
## 6:     1 1-99 0.15   1 300 0.05 0.9490984     1
```

Let us display the true survival curve (in orange), the estimated curves for each dataset (in grey), and their mean (in yellow).

```r
avg_curves = result$survivals[, lapply(.SD, mean), by = time, .SDcols = c("surv0", "surv1")]

library(ggplot2)
library(ggpubr)
p.never = ggplot(result$survivals, aes(x=time, y=surv0, group=factor(rep_b))) +
  geom_line(alpha=0.8, linetype='dashed', col='gray30') + theme_light() +
  geom_line(data = true_surv, aes(x=time, y=true_surv0, group=NULL),
            color = "#FF6633", linetype = "dashed", linewidth = 1) +
  geom_line(data = avg_curves, aes(x=time, y=surv0, group=NULL),
            color = "#FFCC33", linetype = "dashed", linewidth = 1) +
  labs(x='Time t', y='Marginal Survival Probability', title = 'Never treated')

p.always = ggplot(result$survivals, aes(x=time, y=surv1, group=factor(rep_b))) +
  geom_line(alpha=0.8, linetype='solid', col='gray30') + theme_light() +
  geom_line(data = true_surv, aes(x=time, y=true_surv1, group=NULL),
            color = "#FF6633", linetype = "solid", linewidth = 1) +
  geom_line(data = avg_curves, aes(x=time, y=surv1, group=NULL),
            color = "#FFCC33", linetype = "solid", linewidth = 1) +
  labs(x='Time t', y='Marginal Survival Probability', title = 'Always treated')

ggarrange(p.never, p.always)
```

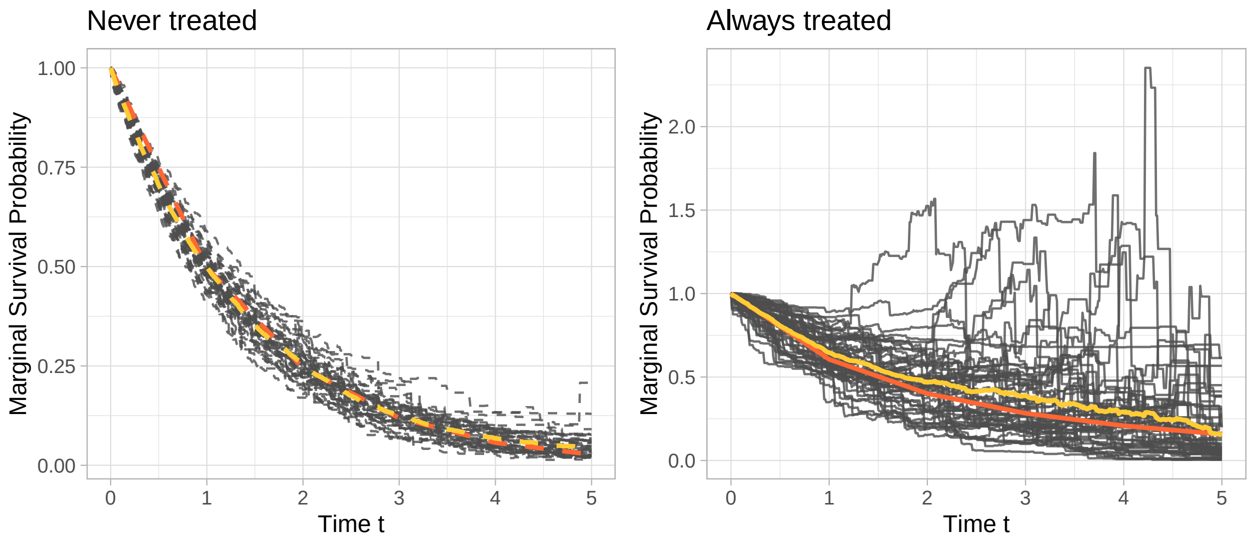


Let us rescale the y-axis for the *always treated*

```
p.always = p.always + coord_cartesian(ylim = c(0, 1))
ggarrange(p.never, p.always)
```

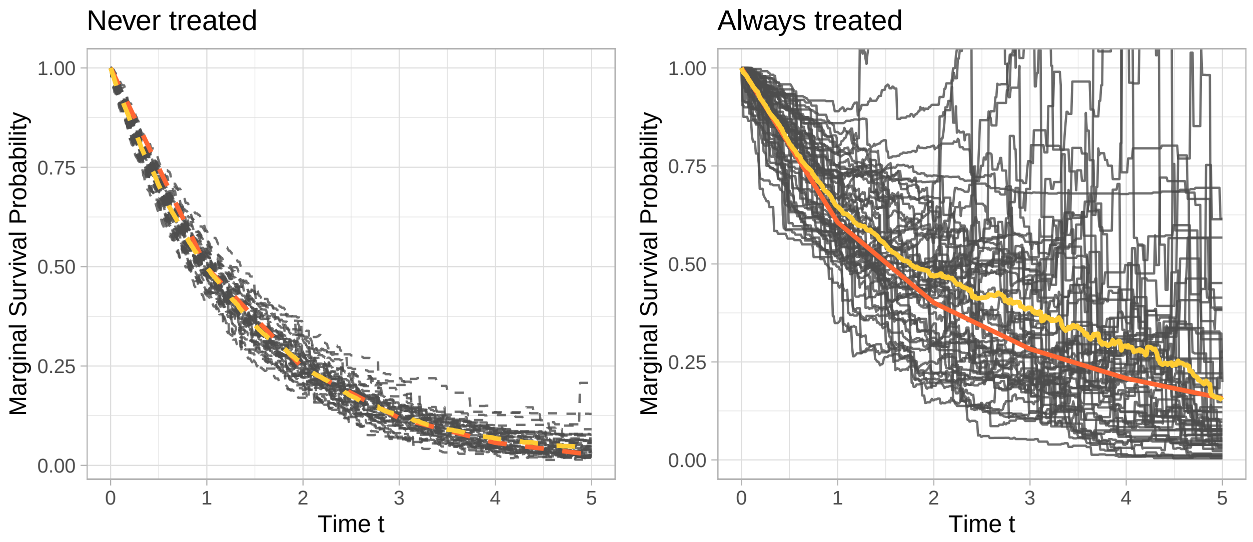



Dr. Marta Spreafico

*Mathematical Institute, Leiden University, Einsteinweg 55, 2333 CC Leiden, The Netherlands*

E-mail: m.spreafico@math.leidenuniv.nl
